# Development of an epigenetic clock to predict visual age progression of human skin

**DOI:** 10.3389/fragi.2023.1258183

**Published:** 2024-01-11

**Authors:** Agata Bienkowska, Günter Raddatz, Jörn Söhle, Boris Kristof, Henry Völzke, Stefan Gallinat, Frank Lyko, Lars Kaderali, Marc Winnefeld, Elke Grönniger, Cassandra Falckenhayn

**Affiliations:** ^1^ Beiersdorf AG, Research and Development, Hamburg, Germany; ^2^ Institute for Bioinformatics, University Medicine Greifswald, Greifswald, Germany; ^3^ Division of Epigenetics, DKFZ-ZMBH Alliance, German Cancer Research Center, Heidelberg, Germany; ^4^ Institute for Community Medicine, SHIP/KEF, University Medicine Greifswald, Greifswald, Germany

**Keywords:** aging, skin aging, DNA methylation, epigenetic age clock, biological age, wrinkles, visual age, age progression

## Abstract

Aging is a complex process characterized by the gradual decline of physiological functions, leading to increased vulnerability to age-related diseases and reduced quality of life. Alterations in DNA methylation (DNAm) patterns have emerged as a fundamental characteristic of aged human skin, closely linked to the development of the well-known skin aging phenotype. These changes have been correlated with dysregulated gene expression and impaired tissue functionality. In particular, the skin, with its visible manifestations of aging, provides a unique model to study the aging process. Despite the importance of epigenetic age clocks in estimating biological age based on the correlation between methylation patterns and chronological age, a second-generation epigenetic age clock, which correlates DNAm patterns with a particular phenotype, specifically tailored to skin tissue is still lacking. In light of this gap, we aimed to develop a novel second-generation epigenetic age clock explicitly designed for skin tissue to facilitate a deeper understanding of the factors contributing to individual variations in age progression. To achieve this, we used methylation patterns from more than 370 female volunteers and developed the first skin-specific second-generation epigenetic age clock that accurately predicts the skin aging phenotype represented by wrinkle grade, visual facial age, and visual age progression, respectively. We then validated the performance of our clocks on independent datasets and demonstrated their broad applicability. In addition, we integrated gene expression and methylation data from independent studies to identify potential pathways contributing to skin age progression. Our results demonstrate that our epigenetic age clock, VisAgeX, specifically predicting visual age progression, not only captures known biological pathways associated with skin aging, but also adds novel pathways associated with skin aging.

## Introduction

Aging is a complex process that involves the gradual decline of physiological functions over time, leading to increased susceptibility to age-related diseases and reduced quality of life. The understanding of aging and its underlying mechanisms has been a major focus of scientific research. However, the complexity and heterogeneity of the aging process present significant challenges in unraveling its intricacies (Flament et al., 2013; Ferrucci et al., 2020). A major challenge in understanding the aging process and developing interventions to promote healthy aging is the lack of reliable biomarkers that can accurately reflect individual differences in the rate of aging and provide insights into the underlying biological processes. (Borras, 2021; Guo et al., 2022; Lopez-Otin et al., 2023).

DNA methylation (DNAm) is an epigenetic modification crucial for regulating gene expression and diverse biological processes, like development and cell function, X-chromosome inactivation or disease pathogenesis. Through the addition of a methyl group to cytosine residues in CpG dinucleotides, DNAm affects gene expression without altering the DNA sequence. DNAm patterns are dynamically regulated during development, cellular differentiation, and in response to environmental cues, contributing to the establishment and maintenance of cell-specific gene expression programs (Jones, 2012; Moore et al., 2013). In addition to global DNAm changes, specific regions known as low methylated regions (LMRs) have emerged as significant regulatory elements. LMRs are characterized by relatively low levels of DNAm and frequently coincide with gene regulatory regions like enhancers and promoters. These evolutionarily conserved LMRs play a role in precise gene expression control and fine-tuning cellular functions (Hu and Dai, 2014; Raddatz et al., 2021). Notably, DNAm patterns undergo alterations during aging, with a common observation of global hypomethylation and site-specific hypermethylation. These age-associated DNAm changes have been implicated in age-related diseases and the decline of various cellular processes (Bollati et al., 2009; Jones et al., 2015; Zampieri et al., 2015). Elucidating the dynamics of DNAm and its modifications during aging holds substantial potential for unraveling the molecular mechanisms underlying aging and developing interventions to promote healthy aging (Marttila et al., 2015).

In response to the need for robust biomarkers of aging, researchers have increasingly focused on the development of epigenetic age clocks. These clocks are molecular instruments designed to estimate an individual’s biological age by examining DNAm patterns. The first generation of epigenetic age clocks, exemplified by the Horvath clock and Hannum clock, were constructed using statistical techniques to identify a collection of DNAm markers that exhibit a correlation with chronological age. These clocks rely on DNAm data obtained from specific genomic regions, which are then utilized to construct a predictive model for age estimation (Hannum et al., 2013; Horvath, 2013). Subsequently, epigenetic age clocks have emerged as invaluable tools within the field of aging research (Armstrong et al., 2017; Horvath and Raj, 2018).

However, first-generation epigenetic age clocks have inherent limitations that hinder their ability to fully capture the intricate nature of the aging process. These clocks primarily rely on a predetermined set of DNAm markers, which may not encompass the entire complexity of aging-related changes. Furthermore, first-generation age clocks often focus solely on estimating chronological age without considering additional influential factors, such as smoking or age-related plasma protein levels (Bell et al., 2019; Levine, 2020). To overcome some of these shortcomings and improve the predictive capacity of age-related phenotype, second-generation epigenetic age clocks have been developed. These clocks integrate DNAm data with supplementary variables such as cognitive or physical performance measures, enabling a more comprehensive evaluation of aging and functional decline (Li et al., 2022). Notably, second-generation age clocks, exemplified by PhenoAge and GrimAge, have shown improved predictive ability for mortality and age-related diseases (Levine et al., 2018; Lu et al., 2019; McCrory et al., 2021). Nevertheless, it is crucial to acknowledge that aging is a multifaceted phenomenon, and although second-generation age clocks outperform their predecessors, they may not fully unravel the underlying mechanisms contributing to the differential rates of aging among individuals (Oblak et al., 2021).

Skin, because of its unique characteristics and the visible manifestations of aging, serves as an exceptional model for studying the aging process. The rate of skin aging exhibits significant variability among individuals, rendering it a valuable tool for capturing diverse age progressions (Lowry, 2020). Notably, the skin is directly exposed to various environmental stressors, including ultraviolet (UV) radiation and pollutants, which contribute to the observable signs of aging, such as wrinkles, age spots, and loss of elasticity (Ansary et al., 2021; Wong and Chew, 2021). These visible and quantifiable aging effects make the skin an ideal model for investigating the pace of aging and assessing the efficacy of interventions. During the aging process, the skin undergoes multifaceted cellular and molecular changes, encompassing epigenetic alterations, that offer valuable insights into the underlying mechanisms of aging (Farage et al., 2009; Raddatz et al., 2013; Krutmann et al., 2017). However, despite the potential advantages of skin as a model system, there is currently a lack of second-generation epigenetic age clocks specifically tailored for skin aging, emphasizing the necessity for further research in this domain.

The recognition of age clocks, which directly capture relevant aging characteristics linked to functional aspects of aging, is increasingly being acknowledged (Field et al., 2018; Salameh et al., 2020). By directing attention towards specific aging markers that closely correlate with age-related deterioration, such as facial visual age progression, the utilization of a skin-specific epigenetic age clock could yield more precise insights into the fundamental molecular processes that underlie interindividual variations in the aging process. This refined approach holds great potential in unraveling the biological mechanisms contributing to age-related decline, thus fostering the development of targeted interventions and personalized strategies aimed at promoting healthy aging.

To follow this notion, we used the methylation patterns of 378 female volunteers as a foundation for developing three second-generation epigenetic age clocks, each targeting the prediction of distinct aging phenotypes including wrinkle grade, visual facial age, and visual age progression. We employed a comprehensive approach that involved testing various training strategies and evaluating the performance of each clock on independent datasets, validating their wide-ranging applicability. Moreover, we specifically tailored our clock, VisAgeX, to effectively predict the visual age progression of skin tissue. Using this newly developed age clock, we integrated gene expression and methylation data from independent studies to identify pathways that may contribute to the rate of skin aging. The results demonstrate that our clock not only captures well-established biological pathways associated with skin aging, but also uncovers novel pathways in the context of skin aging.

## Results

### DNAm-based prediction of the degree of wrinkling

**FIGURE 1:**
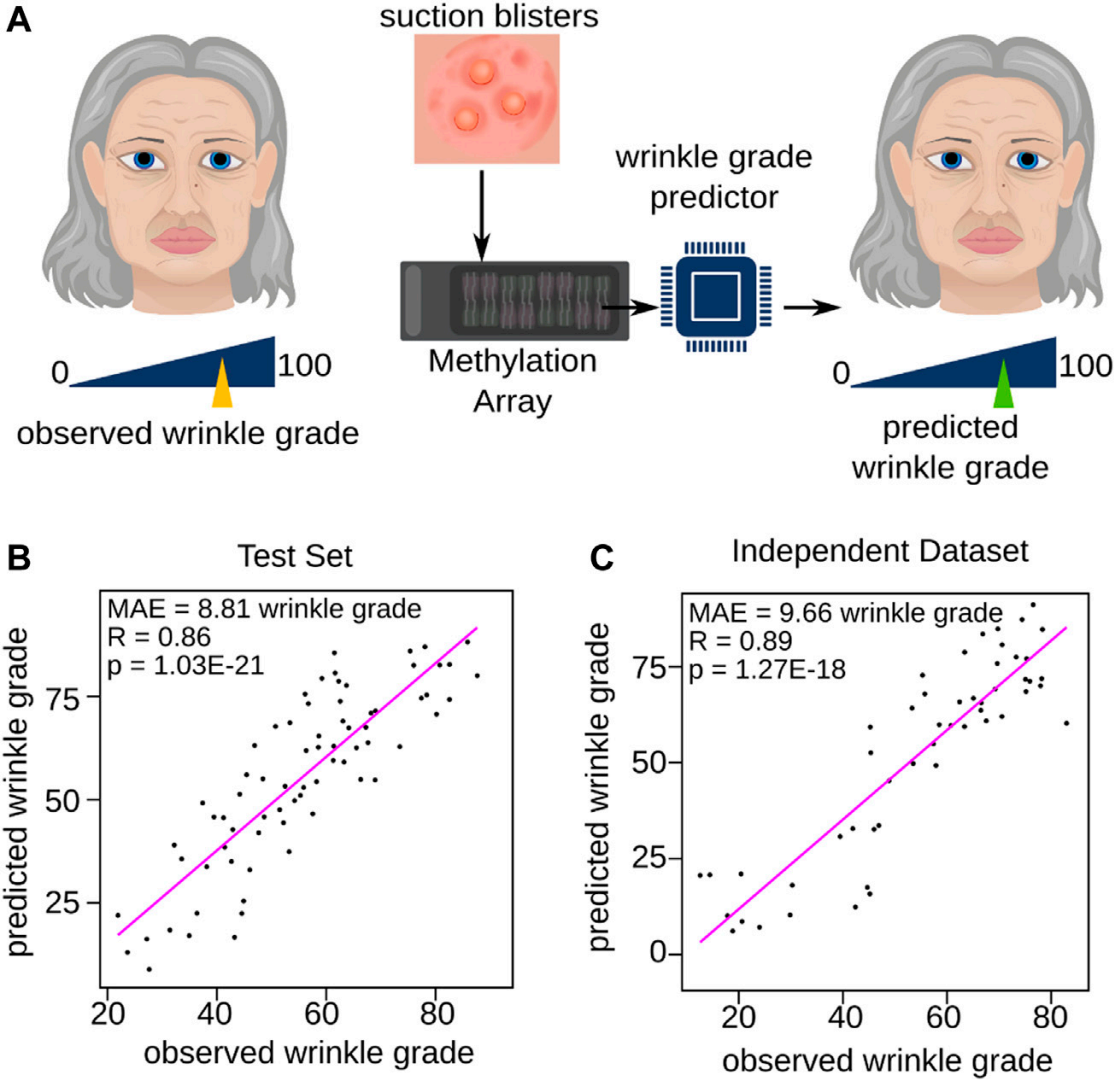
Training of the Wrinkle Predictor. **(A)** Schematic representation of the trained Wrinkle Predictor. For each volunteer, an expert panel judged the grade of wrinkling within a range from 0 to 100. The corresponding DNAm profile of the skin extracted from suction blisters were then used to train a model predicting the wrinkle grade. Validation of the newly trained Wrinkle Predictor on **(B)** the test set and **(C)** the independent dataset (Holzscheck et al., 2020). Each panel reports the Pearson correlation and the mean absolute error.

To test the feasibility of creating a second-generation epigenetic age clock, we first tried to predict the rate of wrinkles as a single skin-aging trait based on DNAm profiles of epidermal samples. We selected wrinkle grade as the primary skin aging trait based on its widespread recognition as a visible sign of aging and the ease of its identification and quantification. Specifically, the degree of facial wrinkle severity was assessed in 378 female volunteers from the Study of Health in Pomerania (SHIP) (Volzke et al., 2022) through the use of a panel of 30 experts who rated the degree of wrinkles on a scale of 1–100 based on portrait photographs of the participants. The wrinkle grade was then determined as the average of the 30 ratings, providing a quantitative measure of skin aging for each participant. This variable was used as outcome variable to train our Wrinkle Predictor based on DNAm data. Initially, the cohort group was divided into two subsets, with 80% allocated for training purposes and 20% reserved for validation. The division ensured that the outcome variable was equally distributed between the training and validation sets. Subsequently, a generalized linear model was trained with 10-fold cross-validation based on the training set (80% of the cohort group, see Materials and Methods and Supplementary Table S1 for details, Figure 1A) leading to a model with a stable mean absolute prediction error among the cross-validations (MAE = 12.66 ± 0.94 wrinkle grade). To validate our model, we applied the remaining 20% of the volunteers which were not used for model training and observed a significant high correlation between predicted and observed wrinkle grade (*R* = 0.86, *p* = 1.03E-21, Pearson correlation), resulting in a low mean absolute error of 8.81 on the wrinkle grade scale (Figure 1B), which was even lower than the median standard deviation of the expert panel (median = 11.85, Supplementary Figure S1). Moreover, we used an independent study (Holzscheck et al., 2020) including 51 healthy females within a similar age range to the SHIP cohort to further validate our Wrinkle Predictor. Again, the predicted wrinkle grade correlated strongly with the wrinkle grade assessed by the experts (MAE = 9.66 wrinkle grade, *R* = 0.89, and *p* = 1.27E-18, Pearson correlation, Figure 1C and Supplementary Table S2). Taken together, we established a DNAm-based Wrinkle Predictor which is applicable for data from multiple sources.

### DNAm-based prediction of visual facial age

**FIGURE 2:**
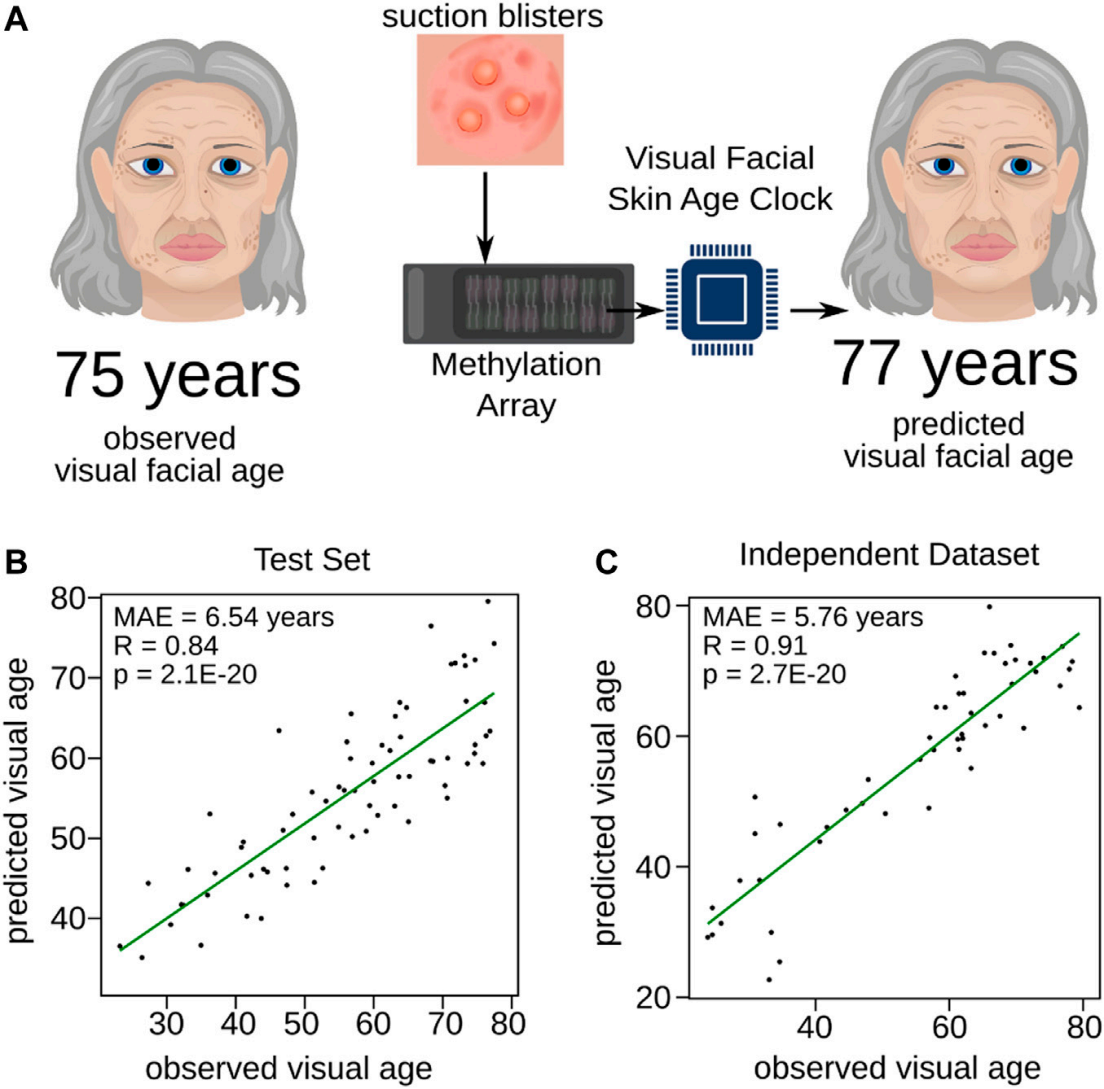
Training of the Visual Facial Skin Age Clock. **(A)** Diagram schematically illustrating the training process for the Visual Facial Skin Age Clock. Expert panels assessed the age of volunteers based on their portrait pictures (observed visual facial age), and the DNAm profiles of skin samples obtained from suction blisters were utilized to train a predictive model for visual facial age (predicted visual facial age). The performance of the newly trained Visual Facial Skin Age Clock was evaluated through validation on **(B)** the test set and **(C)** the independent dataset (Holzscheck et al., 2020). The Pearson correlation and mean absolute error are provided for each panel, indicating the accuracy and precision of the clock’s predictions.

The process of aging exhibits individual variations, leading to diverse manifestation in facial visual appearance. Thus, facial visual age is a reliable measure of age progression as it encompasses the cumulative effects of several factors, such as Sun exposure, lifestyle, and genetic predisposition, that contribute to the aging process (Flament et al., 2013). In comparison, measures such as wrinkle grade provide a limited view of aging by capturing only a single aspect. Facial visual age assessment offers a more holistic perspective on age progression, as it considers various visual aspects of skin aging, including not only wrinkles, but also skin texture, tone, age-related depigmentation, and sagging. To create a tool measuring skin aging more holistically, we trained another DNAm-based model to predict visual facial age (Figure 2A). Similar to the Wrinkle Predictor, we considered the same female participants of which the facial visual age was determined likewise to the wrinkle grade (see Materials and Methods and Supplementary Table S1 for details). The model was trained using 10-fold cross validation on 80% of the dataset again ensuring an equal distribution of the outcome variable, facial visual age.

Again, the mean absolute prediction error of the obtained model was stable among the cross-validations (MAE = 8.20 ± 0.83 years). We then used 20% of the remaining data partition, which was not used to train the model, to validate our Visual Facial Skin Age Clock. We observed a significant correlation (*R* = 0.84, *p* = 2.10E-20, Pearson correlation) between the predicted visual facial age and the assessed age (Figure 2B). In fact, the mean absolute prediction error in the test set (MAE = 6.54 years, Figure 2B) was comparable to the median standard deviation assessed by the expert panel (median = 6.55, Supplementary Figure S2). Furthermore, we validated our Visual Facial Skin Age Clock with the same independent study (Holzscheck et al., 2020) as applied to our Wrinkle Predictor. The predictions from the independent study showed not only a slightly decreased mean absolute prediction error (MAE = 5.76 years), but also an even stronger significant positive correlation (*R* = 0.91, *p* = 2.70E-20, Pearson correlation) compared to the test set (Figure 2C and Supplementary Table S2). In summary, our findings demonstrate that our trained model can predict the visual facial age based on DNAm from different sources.

### Challenges in capturing the rate of skin aging using the visual facial skin aging clock in female volunteers

**FIGURE 3:**
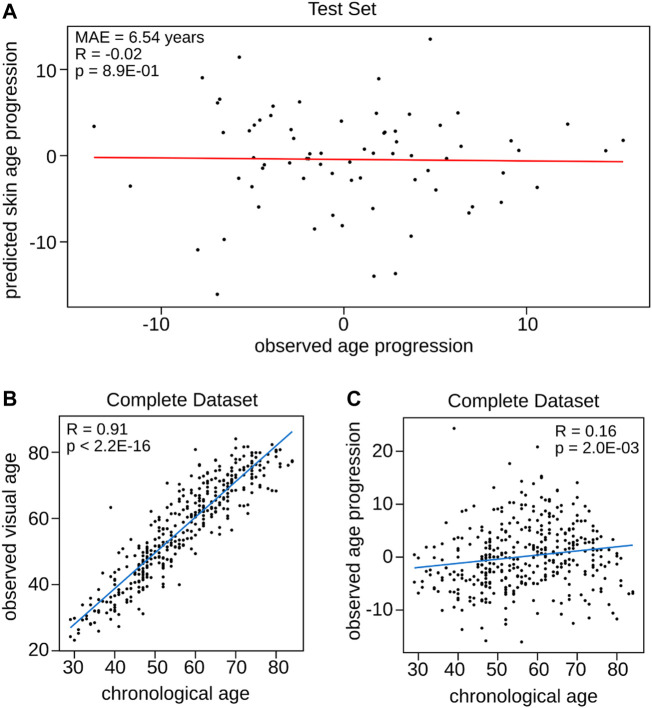
Relationship between observed age progression, predicted skin age progression, chronological age, and observed visual facial age. **(A)** Comparison of the observed age progression, defined as the deviation of visual facial age from chronological age, with the predicted skin age progression obtained computing the difference between the Visual Facial Skin Age Clock’s prediction and actual chronological age. The panel reports the mean absolute error and the Person correlation. **(B)** Relationship between chronological age and observed visual facial age. **(C)** Correlation between chronological age and observed age progression. Each panel includes Pearson correlation.

Two individuals of the same chronological age may differ in visual facial age, indicating that individuals’ skin age varies in rates. To investigate these variations in aging rates and to gain insights in its potential implications for anti-aging interventions we hypothesized that the newly trained Visual Facial Skin Age Clock can be used as tool, as it should capture the difference between visual facial age and chronological age, which we termed skin age progression. For this purpose, we compared the relationship of the observed age progression (the difference between visual facial age assessed by the expert panel and chronological age) and the predicted skin age progression (obtained by calculating the difference between the prediction of the Visual Facial Skin Age Clock and the actual chronological age) (Figure 3A). Here, the observed age progression was defined as the deviation of visual facial age assessed by an expert panel by the chronological age while the predicted skin age progression was calculated by using the Visual Facial Skin Age Clock prediction. Even though the calculated predicted skin age progression obtained by using prediction from the Visual Facial Skin Age Clock resulted in a comparably low mean absolute error of 6.54 years (Figure 2B), the correlation with the observed age progression was not significant and even negative (*R* = −0.02, *p* = 8.90E-01, Pearson correlation, Figure 3A). Moreover, the predicted skin age progression could only explain less than 5% of the variance (|*R*| < 0.05) in the observed age progression. As the Visual Facial Skin Age Clock is not able to capture the association with skin age progression, we conducted separate analyses to understand the general relationship between chronological age and both observed visual facial age (Figure 3B) and observed progression (Figure 3C), respectively.

While chronological age strongly correlated with observed visual facial age (*R* = 0.91, *p* = 2.20E-16, Pearson correlation, Figure 3B), the correlation of chronological age with observed visual skin age progression was much weaker (*R* = 0.16, *p* = 2.00E-02, Pearson correlation, Figure 3C).

### A DNAm clock directly predicting the facial visual age progression

**FIGURE 4:**
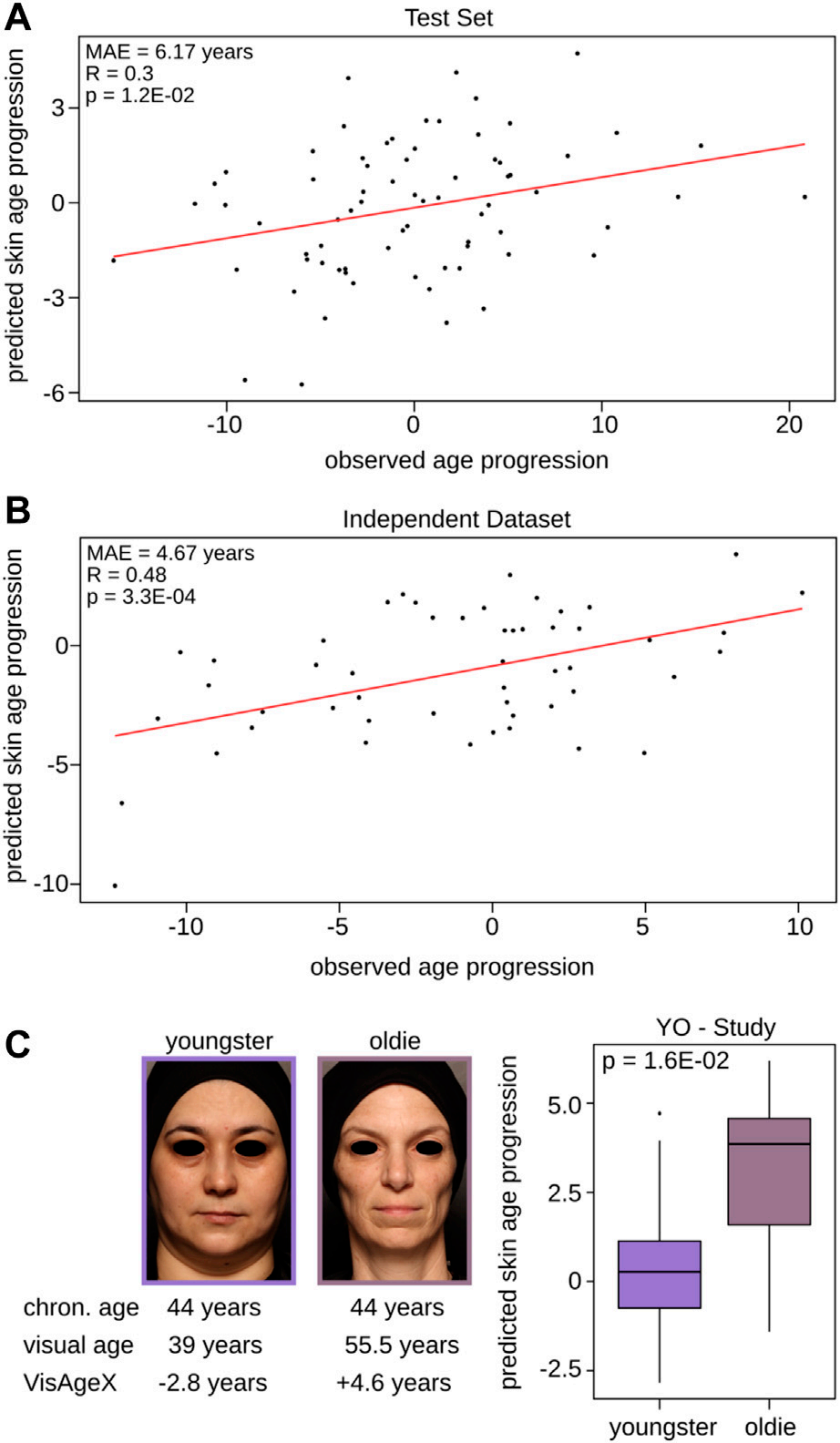
Validation and performance of VisAgeX - the clock trained to predict age progression based on LMRs. Illustration of the comparison between the observed age progression, which represents the deviation of visual facial age from chronological age, and the predicted skin age progression obtained by applying VisAgeX to **(A)** the test set and to **(B)** the independent dataset (Holzscheck et al., 2020). The accuracy and precision of the clock’s predictions are reported in both panels as Pearson correlation and the mean absolute error. **(C)** Discrimination of the age groups Youngsters (age progression <= −5 years) and Oldies (age progression >= +5 years) based on the predicted values of VisAgeX in the Y-O Study. The statistical significance of differentiation was evaluated using the Wilcoxon test. The images and corresponding values below the images on the left serve as demonstrative examples. Chron. age = chronological age.

As the Visual Facial Skin Age Clock could not capture age progression, we trained a DNAm-based model to directly predict the facial visual age progression (VisAgeX clock). Therefore, we only used CpGs located within LMRs for the model input to take advantage of the regulatory potential of these specific regions as they are known for their role in precisely controlling gene expression and fine-tuning cellular functions (see Materials and Methods for details). Following a similar approach to the aforementioned models we split again the SHIP dataset into 80% for the model training and 20% for validation controlling for an equal distribution of the outcome variable, age progression. The obtained model showed a very stable mean absolute prediction error among the cross-validations (MAE = 5.99 ± 0.01 years). Similar to the Wrinkle Predictor and Visual Facial Skin Age Clock, we used the same datasets to validate the performance of VisAgeX (Supplementary Table S2). The prediction obtained by applying VisAgeX to the SHIP test set not only significantly correlated with the observed age progression (*R* = 0.3, *p* = 1.20E-02, Pearson correlation), but also resulted in a low mean absolute prediction error of 6.17 years (Figure 4A). In the case of the independent study (Holzscheck et al., 2020), the mean absolute prediction error decreased even further (MAE = 4.67 years) and displayed a significant correlation (*R* = 0.48, *p* = 3.30E-04, Pearson correlation) of the obtained predictions with the observed age progression values as well (Figure 4B). Additionally, we examined the correlation of VisAgeX predictions with chronological age, which was found to be not significant (*R* = 0.26, *p* = 6.20E-02, Pearson correlation, Supplementary Figure S3). This suggests that the VisAgeX prediction is not primarily focused on DNAm changes associated with chronological age. To further test the performance of VisAgeX in capturing the age progression, we conducted a study, called Youngster-Oldie (Y-O), which was designed for this purpose by recruiting two groups of individuals within a similar chronological age range who exhibited contrasting visual appearances. The first group, referred to as Youngsters, comprised 10 subjects who visually appeared approximately 5 years younger than their chronological age (expert panel assessment), while the second group, referred to as Oldies, consisted of 15 individuals with a similar age to the Youngster group but who appeared approximately 5 years older (expert panel assessment, see Materials and Methods for study details). As this study has been carried out considering two groups of female volunteers with different age progression but within a similar age range, we investigated if the VisAgeX can distinguish both groups. Indeed, a statistically significant differentiation of the youngsters and oldies by the predicted values of VisAgeX was observed (Figure 4C, *p* = 1.6E-02, Wilcoxon test).

Additionally, we also compared the performance of VisAgeX trained based on LMRs compared to VisAgeX trained based on all CpGs of the Infinium MethylationEPIC v1.0 BeadChip (Supplementary Figure S4 and Supplementary Table S2). In contrast to the LMR-based VisAgeX, the mean absolute prediction error for the CpG-based clock was slightly increased in the SHIP test set (MAE = 6.26 years >6.17 years, Supplementary Table S2) and considerably increased in the independent study (MAE = 6.23 years >4.67 years, Supplementary Table S2), accompanied by a decrease in the significance of the correlations (*p* = 2.30E-02 > 1.20E-02, Pearson correlation, and *p* = 2.90E-03 > 3.30E-04, Pearson correlation, respectively, Supplementary Table S2). Furthermore, the two distinct groups of the Y-O study could not be differentiated statistically significant by the predicted values of the CpG-based VisAgeX clock (*p* = 3.40E-01, Wilcoxon test, Supplementary Figure S4C and Supplementary Table S2) in contrast to the values of the LMR-based clock (*p* = 1.6E-02, Wilcoxon test, Figure 4C and Supplementary Table S2). Thus, the CpG-based VisAgeX clock did not pass validation in all three studies.

### Identification of pathways potentially contributing to the speed of skin aging

**FIGURE 5:**
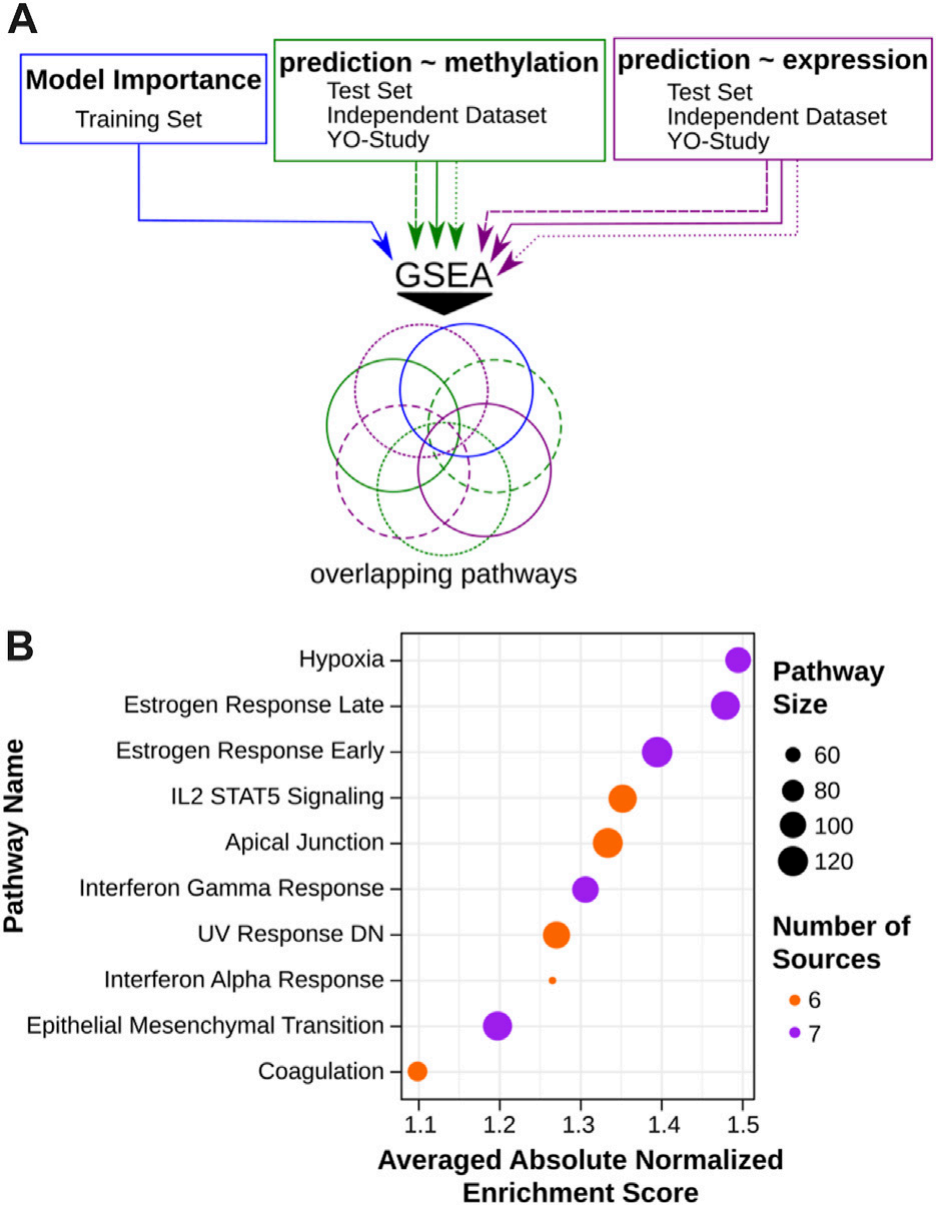
Assessment of biological pathways and predictive features of VisAgeX. **(A)** Schematic representation of the pathway enrichment analyses which were conducted using three different approaches to evaluate the potential applicability of VisAgeX for investigating the biological mechanisms driving the aging process. The first approach ranked genes based on the coefficient values from the model (blue), the second approach ranked genes based on the correlation between CpG methylation level and predicted visual facial age progression determined by VisAgeX (green), and the third approach correlated gene expression data with VisAgeX predictions (purple). These approaches were applied to the test set, the independent study (Holzscheck et al., 2020), and the Y-O study used for VisAgeX validation. The resulting lists of pathways were then analyzed for overlaps to ensure general validity across different studies. **(B)** Representation of the top ten overlapping biological pathways visualized in a dot plot with dot size refereeing to the pathway size, the color to the number of overlapping sources and sorted according to their averaged absolute normalized enrichment score.

In order to evaluate the potential utility of VisAgeX as a tool for investigating the biological mechanisms underlying aging, we performed pathway enrichment analyses using three distinct approaches (Figure 5A). Generally, we used a ranked gene list as input for GSEAPreranked to identify enriched pathways out of the biological Hallmarks, as defined in MSigDB (see Materials and Methods for details) (Mootha et al., 2003; Subramanian et al., 2005; Liberzon et al., 2015). First, we used the coefficient values from the model as importance score to rank the genes associated to skin-specific LMRs (Figure 5A, blue). In the second approach, the genes were ranked based on the correlation between methylation level and predicted skin age progression determined by VisAgeX (Figure 5A, green). Lastly, the available gene expression data were correlated to the VisAgeX prediction to rank the genes for the pathway enrichment analysis (Figure 5A, purple). The latter two approaches were applied to the three datasets SHIP test set, the independent study (Holzscheck et al., 2020) and the Y-O study, as used for the validation of VisAgeX. Thus, the analyses resulted in 7 lists of biological hallmarks, from which the overlapping pathways were considered to ensure a general validity independent of the underlying studies. Finally, a list of 10 pathways was obtained, 5 revealed by all 7 analyses and 5 that were revealed by 6 of the 7 analyses (Figure 5B). In our analysis, we identified various biological pathways that may be potentially involved in the speed of skin aging. These pathways include established skin aging-related processes, such as those associated with early and late estrogen responses and genes downregulated by UV radiation. Additionally, pathways not commonly linked with skin aging, including hypoxia response and epithelial-mesenchymal transition (EMT) in wound healing, fibrosis, and metastasis, were also identified. In summary, our analysis thus revealed ten potential biological pathways that could contribute to the speed of skin aging.

## Discussion

Aging is a multifaceted process characterized by a gradual decline in physiological functions, which increases the susceptibility to age-related diseases (Lopez-Otin et al., 2023). Consequently, there is an increasing demand for precise and reliable aging indicators to enable monitoring and prediction of biological aging rates in individuals. Nonetheless, a comprehensive understanding of the mechanisms of aging and why people age at different rates is still lacking (Kennedy et al., 2014; Levine et al., 2022). Our study demonstrates the successful prediction of the wrinkle grade, visual facial age, and visual age progression, respectively, using DNAm profiles of epidermal samples, indicating the potential of DNAm as a reliable predictor of skin-aging traits. Furthermore, our three second-generation skin-specific epigenetic age clocks were subjected to validation on independent datasets to ensure their reliability and generalizability. In all cases we observed a significant and strong correlation between the predicted and observed values, demonstrating the wide-ranging applicability of our trained models. Notably, the practical application of our wrinkle clock also confirmed the rejuvenating effect of the small compound dihydromyricetin (DHM) on aged human skin (see accompanying paper by Falckenhayn et al.). The findings revealed that DHM not only reduced the wrinkle grade prediction in cultured primary human keratinocytes but also resulted in a reduction in methylation age as validated by two independent epigenetic age clocks. The predicted rejuvenating effects were accompanied by functional anti-aging effects on skin cells. This highlights that our predictor is indeed capable of mathematically modulating the skin aging phenotype.

Although our Visual Facial Age Clock accurately predicts the visual facial age, the model’s ability to capture skin age progression was weak, as the results showed a non-significant correlation with the predicted skin age progression explaining less than 5% of the variance. This discrepancy may be attributed to the weak correlation between chronological age and observed visual skin age progression. The visual judgment of age is typically learned and based on experiences with chronological age, whereas the DNAm changes with chronological aging may not directly align with the observed age progression (Ryan, 2021; Simons et al., 2021). Age progression, being a measure of divergence from chronological age, is influenced by various factors including environmental influences. Therefore, while the visual facial age can be predicted based on DNAm, it does not correlate strongly with the age progression.

We were able to overcome this limitation by training a clock directly using the speed of aging as a proxy. The prediction of the newly developed age clock, VisAgeX, showed a significant positive correlation with observed age progression in all applied datasets, although the correlation was relatively weak. This can be attributed to several technical and biological factors. One possible explanation is that the clock may not capture all the relevant molecular markers associated with age progression, as aging is a complex process influenced by various molecular pathways. To support this hypothesis, studies utilizing multi-omics approaches, such as the GrimAge approach, have shown improved prediction accuracy by incorporating additional molecular features beyond DNAm alone (Lu et al., 2019; Kudryashova et al., 2020; Di Micco et al., 2021). Our results indicate that the developed clock holds promise in predicting age progression and highlights the need for continued exploration of multi-omics approaches and the integration of diverse biological factors to improve the prediction accuracy of skin age progression which could be a promising basis for refining our understanding of aging mechanisms.

Interestingly, our results also indicate that the clock achieved by LMR-based training outperforms the traditional CpG-based approach in predicting visual age progression, providing a more accurate reflection of skin aging. We believe that this superior performance can be attributed to the LMRs’ tissue specificity and their sensitivity to dynamic changes influenced by environmental and biological factors. LMRs provide a more comprehensive representation of the aging process (Hu and Dai, 2014; Raddatz et al., 2021), yielding more accurate predictions of skin age progression. Our findings highlight the importance of considering LMR-based approaches in developing predictive models for aging and suggest the potential of VisAgeX as a valuable tool for assessing the rate of aging. This provides a promising outlook for the application of LMRs in developing more accurate and reliable aging clocks.

Moreover, our analysis of the VisAgeX clock identified diverse biological pathways known for their association with skin aging, including estrogen response, which has been shown to play an important role in maintaining dermal health and anti-aging properties by binding to estrogen receptors in the skin, thereby affecting collagen and elastin profiles during aging and at/post menopause (Ganceviciene et al., 2012; Thornton, 2013; Lephart and Naftolin, 2022). It also highlighted the importance of the response to UV radiation which is known to be a major driver of photoaging, characterized by the generation of reactive oxygen species (ROS), upregulation of matrix metalloproteinases (MMPs), particularly MMP2, and degradation of collagen and other extracellular matrix components, contributing to wrinkle formation and skin aging (Gronniger et al., 2010; Rinnerthaler et al., 2015; Kim et al., 2022). Additionally, our investigation uncovered the involvement of other pathways that may contribute to skin aging, such as hypoxia and epithelial-mesenchymal transition (EMT). In this regard, studies in *Caenorhabditis elegans* have demonstrated that the modulation of hypoxia-inducible factors (HIF) signaling pathways influence the rate of aging and lifespan. For example, the activation of HIF can induce a state of cellular senescence, characterized by irreversible growth arrest and the secretion of pro-inflammatory molecules. Additionally, it has been demonstrated that manipulating HIF signaling pathways can extend lifespan and delay age-related phenotypes in *C. elegans* (Chen et al., 2009; Leiser et al., 2011; Hwang et al., 2014). These studies suggest an intricate relationship between hypoxia and aging, necessitating further research to elucidate its effects. Furthermore, emerging evidence suggests that EMT also has implications for aging and lifespan. EMT is a cellular process characterized by the conversion of epithelial cells into mesenchymal-like cells and plays a critical role in embryonic development, tissue repair, and cancer progression. With advancing age, there is a disruption of EMT, leading to an accumulation of mesenchymal-like cells in various tissues. Abnormal EMT has also been associated with age-related tissue dysfunction, fibrosis, and impaired regenerative capacity. Additionally, EMT-related factors, such as transforming growth factor-beta (TGF-β), have been implicated in the regulation of aging and lifespan in model organisms. Further investigations are required to unravel the precise molecular mechanisms underlying the interaction between EMT and aging, which may offer potential insights into therapeutic strategies for age-related diseases (Santos et al., 2019; Imran et al., 2021).

Although VisAgeX has shown promising results, our study has identified several limitations that require attention in future research. Firstly, the clock was trained exclusively on data from female participants, which raises concerns about its generalizability to males. However, our decision to use only female data was justifiable, as we can observe gender-driven differences in aging process (Hägg and Jylhävä, 2021). Moreover, the correlation between visual facial skin age and inner volar forearm skin age, while promising, may be influenced by external and systemic factors, which cautions against further generalization, especially regarding the effects of Sun exposure. Our choice of forearm samples prioritized donor compliance and convenience, but the resulting data may not fully reflect the epigenetic and gene expression patterns of facial skin, requiring careful consideration when interpreting our findings. Additionally, it is essential to acknowledge that our study only tested the clock’s prediction in a group of Caucasian volunteers, and the generalizability of our findings to other ethnicities is yet to be explored. Future studies in this area should include diverse ethnic groups to improve the general applicability of the clock (Garza et al., 2017). Further investigation of these limitations may lead to a better understanding of the molecular mechanisms underlying skin aging and the development of more effective anti-aging interventions.

In conclusion, we successfully developed a skin-specific epigenetic age clock, VisAgeX, which captured differences in facial visual age progression. By testing various training strategies, we were able to optimize the clock’s performance and establish its correlation with facial visual age progression. Our findings regarding the biological pathways may provide a deeper insight into the molecular mechanisms underlying age progression, and thus, could have implications for future research. Moreover, VisAgeX can be used to assess the impact of internal and external factors, such as UV exposure and pollution, on skin aging, but further analysis will be necessary. The development of VisAgeX represents a significant advancement towards understanding the aging process and may serve as a basis for future investigations into the functional aspects influencing the speed of aging.

## Materials and methods

### Ethical approval

Ethical approval was obtained in consideration of the Declaration of Helsinki and the guideline of the International Conference on Harmonization Good Clinical Practice (ICH GCP) by the International Medical & Dental Ethics Commission in Hamburg (Std. no. 67686) and by the Independent Ethics Committee Freiburg (feki code 08/2610).

### Studies used in this publication

We used portrait images as well as methylation and gene expression data from epidermal samples of three different studies (Supplementary Table S1): the population-based Study of Health in Pomerania (SHIP-TREND-1) (Volzke et al., 2022), the independent study (Holzscheck et al., 2020) and the Y-O study. The first study involved 378 female participants aged 29–84 years and served as data pool for the training of the models. In the second study, we used samples from 51 women aged 23–84 years (Holzscheck et al., 2020). The third study, known as the Y-O study, examined samples from 25 women aged 41–51 years who visually appeared at least 5 years younger (*n* = 10 females) or older (*n* = 15 females) than their chronological age. Both groups of the Y-O study were similar in chronological age (Youngsters = 45.30 ± 2.11 years, Oldies = 46.40 ± 3.00 years), but different in expert-assessed visual facial age (Youngsters = 40.24 ± 2.61 years, Oldies = 58.16 ± 5.39 years). A detailed overview of the inclusion and exclusion criteria for the Y-O study can be found in Supplementary List S1.

### Capturing portrait images and assessment by an expert panel

Portrait photographs of the participants were taken using the Visia CR Skin Analysis Imaging System (Canfield Scientific, Inc.). This system uses visible light imaging technology to capture high-resolution digital images of the skin surface. Participants were carefully positioned according to system guidelines to ensure standardized and consistent positioning across subjects prior to image acquisition. The Visia CR System uses visible light to help visualize various skin conditions, including blemishes, wrinkles, texture variations and pigmentation irregularities.

The wrinkle grade was determined for each volunteer using portrait photos. The datasets used for this assessment consisted of volunteers from two previous studies (Holzscheck et al., 2020; Volzke et al., 2022). In the visual assessment of wrinkles, trained experts performed a visual analysis of an individual’s face and graded the wrinkles on a scale of 1–100, with 1 indicating no wrinkles and 100 indicating a high degree of wrinkles. This method employs pre-defined grading scales as seen in the “Atlas du Vieillissement Cutané - Population Européenne” (Skin Aging Atlas - European Population) (Bazin and Doublet, 2007). To minimize subjectivity, more than 30 experts evaluated the same volunteer and the visual wrinkle grade was the average of their evaluations.

In addition to evaluating the wrinkle grade, the experts also evaluated the visual facial age of the volunteers based on their portrait photos. This comprehensive assessment was conducted not only for volunteers from the previous studies (Holzscheck et al., 2020; Volzke et al., 2022), but also for participants in the Y-O study. Each expert individually rated the visual facial age of every volunteer, and an average visual facial age was derived from these ratings. The visual facial age serves as an estimation, capturing the perceived age of an individual based on their appearance in the portrait photos.

### Tissue sample preparation

In all studies suction blister roofs of 7 mm diameter were collected from the volar forearms by applying a negative pressure of 180 mbar for 30 min followed by 320 mbar until blister formation and subsequent preparation with surgery scissors and tweezers. The suction blister roofs were snap frozen in liquid nitrogen and stored at −80°C until further use.

### Array based methylation profiling and data pre-processing

Genomic DNA was isolated from the suction blister roofs using the QIAamp DNA Investigator Kit (Qiagen, Inc.) following the manufacturer’s instructions. Subsequently, the isolated genomic DNA was processed on EPIC Methylation arrays (Illumina, Inc.) to obtain the DNAm patterns.

We utilized the R Bioconductor minfi package (Aryee et al., 2014; Fortin et al., 2017) to pre-process the raw.idat files from the EPIC arrays. A series of filtering steps were applied to ensure data quality. First, methylation loci (probes) were filtered based on high detection p-values (*p* > 0.01). Then, probes were filtered based on their self-hybridization ability and potential SNP contamination resulting in a total number of 794,441 CpGs. To normalize the methylation data, we performed matrix normalization using quantile normalization with the “preprocessQuantile” function provided by the minfi package (Aryee et al., 2014; Fortin et al., 2017). Quality control checks were performed after each pre-processing step to monitor the integrity and reliability of the data.

### Identification of skin specific LMRs

First, we combined whole-genome bisulfite sequencing (WGBS) methylation data for epidermis of young and old subjects (Raddatz et al., 2013) leading to an average strand-specific CpG coverage of 14x. To identify LMRs we applied MethylSeekR (Burger et al., 2013) using standard parameters to this dataset and selected LMRs, which were overlapping with at least one probe of the EPIC Methylation array. This led to a set of 40,140 LMRs. For further analysis, we calculated the average beta value for each LMR by considering the CpGs located within that specific region.

### DNAm-based second-generation epigenetic age clock training

The dataset, consisting of 378 females (Volzke et al., 2022), was divided into training and testing subsets, with 80% allocated for training and 20% for testing. The “createDataPartition” function from the caret package (Kuhn, 2022) was utilized to control for equal distribution of the outcome variable between both subsets. The Wrinkle Predictor was then trained using the beta values of the training dataset, using a generalised linear model with ridge regression fit (alpha = 0) to ensure that no features (CpGs) were excluded as part of our preprocessing for training. This approach was facilitated by the “cv.glmnet” function (lambda = 1708.46) from the glmnet R package (Friedman et al., 2010) within a 10-fold cross-validation framework with lambda optimalization. Basically, the “cv.glmnet” function, divides the provided training set of 302 females automatically into n-fold training and test sets within each cross-validation. To determine the optimal lambda value, the algorithm initially selects a high lambda and performs a 10-fold cross-validation, calculating the prediction error. It then systematically reduces the lambda value (up to 100 times), conducting 10-fold cross-validation and calculating the prediction error for each lambda. The model’s performance was reported using the mean absolute error with its standard deviation (SD), considering all examined lambda values during the algorithm’s execution. Here, the wrinkle grade, as assessed by the expert panel, served as the outcome variable during training (Figure 1A).

The Visual Facial Skin Age Clock was trained following a methodology similar to that of the Wrinkle Predictor with following parameters: alpha set to 0 and lambda set to 1,402.16, whereas the expert-assessed visual facial age instead of wrinkle grade served as the outcome variable (Figure 2A).

To train the clock predicting age progression directly, a similar approach to that of the Wrinkle Predictor and Visual Facial Skin Age Clock was employed using either methylation levels of 40,140 LMRs or all CpGs. In both training approaches, an alpha value of 0 was utilized, along with a lambda of 19.45 for the LMR-based clock training and a lambda of 295.39 for the traditional CpG-based approach. Notably, during training, we used the observed age progression (defined as the deviation of visual facial age from chronological age) as the outcome variable. The resulting clock was named VisAgeX (LMR-based) and CpG-based Visual Facial Age Progression Clock, respectively.

All trained DNAm-based skin-specific second-generation epigenetic age clocks were subsequently validated using both the test set and an independent dataset (Holzscheck et al., 2020). Subsequently, Pearson correlation was used to validate the clock performance by comparing the observed value with value predicted by the clock (Supplementary Table S2). In addition to the aforementioned studies only VisAgeX was further validated by using the data from the Y-O study. The statistical significance of the differences in predicted age progression between the two groups of the Y-O study (see Studies used in this publication for details) was determined via Wilcoxon test (“wilcoxon.test” function) from the stats R package (R Core Team, 2021).

### RNA-Seq based transcriptome profiling and data pre-processing

Total RNA was extracted from the suction blister roofs using the RNAeasy Fibrous Tissue Mini Kit (Qiagen, Inc.) following the manufacturer’s instructions. Sequencing was performed on Illumina’s HiSeq system in single end mode with a read length of 50 bp to a final sequencing depth of 100 million reads per sample.

Generated raw reads were processed as follows: i) quality control using Fastqc 0.11.7 (Andrews, 2007), ii) trimming of read sequences via Trimmomatic 0.36 (Bolger et al., 2014) and iii) mapping of reads against the GRCh38 build of the human transcriptome using Salmon 0.8.1 (Patro et al., 2017). Subsequently, mapped reads were quantified as transcript per million (TPMs) on gene level and used for downstream analysis.

### Pathway-enrichment analysis

To evaluate the potential utility of VisAgeX as a tool for investigating the biological mechanisms of aging, we conducted the GSEAPreranked (Mootha et al., 2003; Subramanian et al., 2005) analysis using ranked gene lists obtained from three distinct approaches. The first approach involved using coefficient values from the model as importance scores to rank the genes (Figure 5A, blue). In this process, CpGs located in the LMR genes were annotated based on the Infinium MethylationEPIC v1.0 B5 Manifest File (Supplementary Table S3), and the coefficient values were used as ranked genes. To avoid repetition for repeated genes in the list, the rank values were summed up. The reason for summing up the rank values is rooted in the linear model design and aims to prevent duplication of genes in the list.

In the second approach, genes were ranked based on the Pearson correlation between CpG methylation levels and predicted age progression determined by VisAgeX (Figure 5A, green). CpGs with a *p*-value above 0.05 were discarded, and the remaining CpGs were annotated to genes using the Infinium MethylationEPIC v1.0 B5 Manifest File. The correlation values were then used as weights for these genes. For repeated genes, their average weight was calculated.

The third approach involved correlating the available gene expression data with the VisAgeX predictions using Pearson correlation. Genes with a p-value correlation exceeding 0.05 were filtered out, and for repeated genes, their average weight was calculated (Figure 5A, purple). The latter two approaches were applied to the SHIP test set, the independent study (Holzscheck et al., 2020), and the Y-O study, which was used for VisAgeX validation. Consequently, this resulted in seven GSEAPreranked inputs.

Subsequently, the Hallmark Process gene sets downloaded from the Molecular Signature Database (MSigDB) (Liberzon et al., 2015) were utilized for the analysis with GSEAPreranked software (Mootha et al., 2003; Subramanian et al., 2005). The GSEAPreranked was performed with default parameters, including 1,000 permutations of gene sets and weighted enrichment statistics. From the analysis, we selected up to 20 of the most discriminative traits for further study. To provide an overall validation of the pathways that may be relevant for VisAgeX prediction, we considered the overlapping results from all seven inputs (Figure 5A). As a result, we present a list of the top ten pathways (Figure 5B).

## Data Availability

The data presented in the Y-O study are deposited in the Genome Expression Omnibus (GEO) repository, accession number GSE249225.
