# Supplementary material for: Development of an epigenetic clock to predict visual age progression of human skin
Source: Front Aging. 2024 Jan 11;4:1258183. doi: 10.3389/fragi.2023.1258183 (PMC10809641; doi:10.3389/fragi.2023.1258183)
Supplement: Supplementary file 1 [file DataSheet3.pdf]

Supplementary Material

Development of an epigenetic clock to predict visual age progression of human skin

Agata Bienkowska[1,2]*, Günter Raddatz[3], Jörn Söhle[1], Boris Kristof[1], Henry Völzke[4], Stefan Gallinat[1], Frank Lyko[3], Lars Kaderali[2], Marc Winnefeld[1], Elke Grönniger[1]*, and Cassandra Falckenhayn[1]

[1] Beiersdorf AG, Research and Development, Hamburg, Germany

[2] Institute for Bioinformatics, University Medicine Greifswald, Greifswald, Germany

[3] Division of Epigenetics, DKFZ-ZMBH Alliance, German Cancer Research Center, Heidelberg, Germany

[4] Institute for Community Medicine, SHIP/KEF, University Medicine Greifswald, Greifswald, Germany

*** Correspondence:**
Agata Bienkowska
agata.bienkowska@beiersdorf.com

Elke Grönniger, Dr.
elke.groenniger@beiersdorf.com

1 Supplementary Figures and Tables

1.1 Supplementary Figures

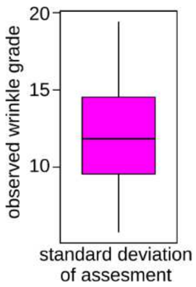

Supplementary Figure S1. Analysis of the wrinkle grade ratings provided by the expert panel. Boxplot representing the standard deviation of the wrinkle grade assessments with a median value of 11.85 wrinkle grade.

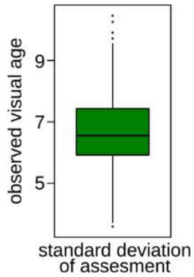

Supplementary Figure S2. Visualization of visual facial age rating variability by the expert panel. Boxplot displaying the standard deviation of the visual facial age ratings with a median of 6.55 years.

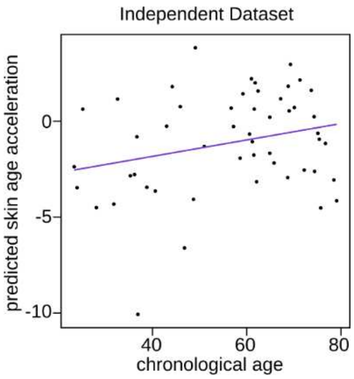

Supplementary Figure S3. Relationship between VisAgeX prediction and chronological age. Scatter plot comparing the chronological age of the subjects with the predicted skin age progression obtained with VisAgeX using the independent data set (Holzscheck et al., 2020). The correlation and its significance of this relationship were measured by the Pearson correlation coefficient (R = 0.26, p = 6.20E-02).

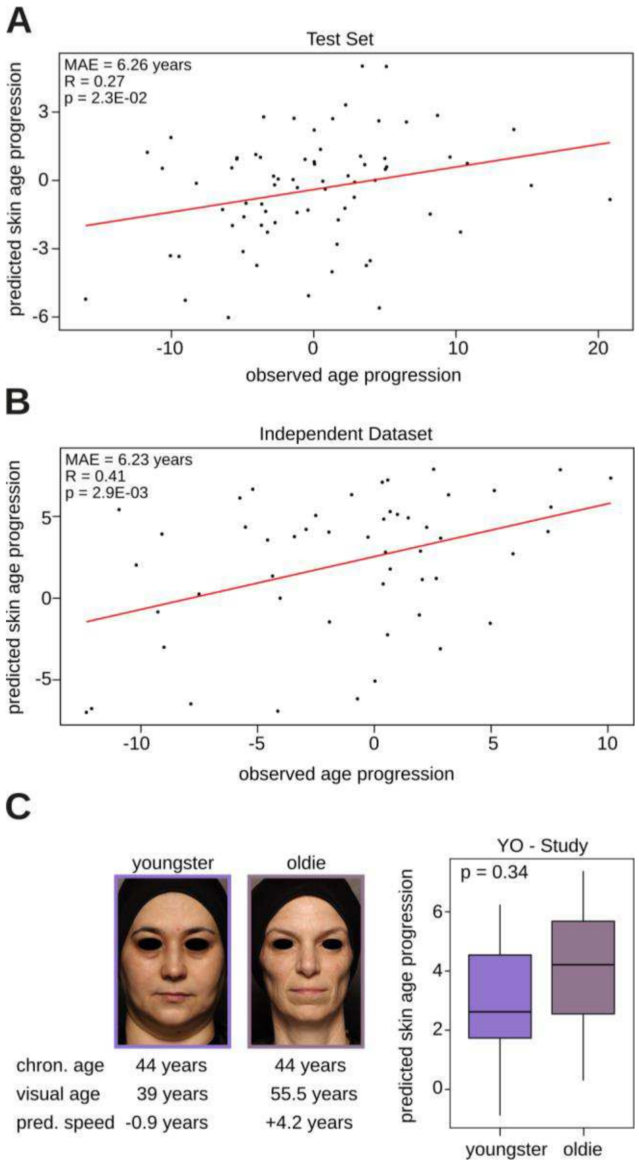

Supplementary Figure S4. Validation of the CpG-based Visual Facial Age Progression Clock. Scatter plot comparing the observed skin age progression, representing the disparity between visual facial age and chronological age, with the predicted skin age progression obtained using the CpG-based Visual Facial Age Progression Clock by applying (**A**) the test set and (**B**) the independent dataset (Holzscheck et al., 2020). The accuracy and precision of the clock's predictions are illustrated in both panels A and B as the Pearson correlation coefficient and mean absolute error. (**C**) Discrimination of the age groups Youngsters (age progression <= -5 years) and Oldies (age progression >= +5 years) based on the predicted values of the CpG-based Visual Facial Age Progression Clock. The differentiation between the age groups was assessed for statistical significance using the Wilcoxon test. The images and corresponding values below the images on the left serve as demonstrative examples. Chron. Age = chronological age.

1.2 Supplementary Lists

Supplementary List S1. Recruitment Appendix - Y-O Study Inclusion and Exclusion Criteria. Detailed inclusion and exclusion criteria for the recruitment process are provided below:

Inclusion criteria:
- Female volunteer in a generally good state of health
- Healthy, intact skin in the test areas
- Ability to understand the study (intellectually/mentally, linguistically)
- Willingness to abide by the protocol and study rules
- Willingness to provide written informed consent

Exclusion criteria:
- Facial scars or acne
- Rosacea/psioriasis/neurodermatitis of the face
- Menopause
- Presence of photosensitive epilepsy
- Heavy smoker (more than 10 cigarettes per day)

General exclusion criteria:
- Concurrent participation in studies at other testing institutes
- Concurrent participation in Beiersdorf studies involving the same test area and/or the same parameter (e.g., IUT)
- Pregnancy and/or breastfeeding
- Insufficient knowledge of German (spoken and written)
- Participation in the study under the influence of or dependence on alcohol and/or drugs
- Obesity with a BMI > 33
- Severe mental illness or mental incapacity to understand the study

Exclusion criteria regarding the test area:
- Presence of a chronic (e.g., atopic dermatitis, psoriasis) or current skin disease in the test area
- within the last 14 days before the start of the study and/or during the study:
  - Use of ointments, creams or similar products for antibacterial, anti-allergic or immunosuppressive treatment of the skin in the test area
  - Use of physical and/or cosmetic treatments in the study area

Underlying disease exclusion criteria:
- Cancer diseases in the past 10 years
- Presence of a severe underlying disease (e.g., severe cardiovascular, liver, kidney, lung disease, severe diabetes mellitus) or chronic infectious disease (e.g., hepatitis, HIV)
- Proven allergy to cosmetic ingredients, plasters, cotton wool or anamnestic intolerance reactions after application of cosmetic products

Exclusion criteria regarding acute diseases:
- within the last 14 days before the start of the study and/or during the study:
  - use of antihistamines and/or
  - use of immunosuppressive drugs (e.g., corticoids, cytostatics) and/or retinoids
- within the last 7 days prior to study entry: illnesses accompanied by fever (38.5°C or higher) for a duration of at least 24 hours

Additional eligibility criteria:
- Phototype from 1 to 3

1.3 Supplementary Tables

Supplementary Table S1. An overview of the studies involved in the training and validation of VisAgeX. It outlines the sample sizes within each cohort used for these processes and details the specific methods used to normalize methylation data and quantify transcriptomic data. In addition, these validation sets played a central role in both validating the performance of the VisAgeX and exploring pathways through pathway-enrichment analysis.

<table>
<tr><th colspan="2">Study Name</th><th colspan="2">SHIP-TREND-1 (Volzke et al., 2022)</th><th>independent study (Holzscheck et al., 2020)</th><th>the Y-O study</th></tr>
<tr><td colspan="2" rowspan="2">Sample Size</td><td colspan="2">378</td><td rowspan="2">51</td><td rowspan="2">25</td></tr>
<tr><td>80%</td><td>20%</td></tr>
<tr><td colspan="2">Data Category</td><td>training set</td><td>validation set</td><td>validation set</td><td>validation set</td></tr>
<tr><td colspan="2">Gender</td><td colspan="2">female</td><td>female</td><td>female</td></tr>
<tr><td rowspan="6">Methylation Data</td><td>Normalisation</td><td colspan="2">quantile</td><td>quantile</td><td>quantile</td></tr>
<tr><td>Wrinkle Predictor</td><td>training</td><td>validation</td><td>validation</td><td>validation</td></tr>
<tr><td>Visual Facial Skin Age Clock</td><td>training</td><td>validation</td><td>validation</td><td>validation</td></tr>
<tr><td>CpG-based Visual Facial Age Progression Clock</td><td>training</td><td>validation</td><td>validation</td><td>validation</td></tr>
<tr><td>VisAgeX</td><td>training</td><td>validation</td><td>validation</td><td>validation</td></tr>
<tr><td>Pathway Enrichment</td><td>yes</td><td>yes</td><td>yes</td><td>yes</td></tr>
<tr><td rowspan="2">Transcriptomic</td><td>Quantification</td><td colspan="2">TPMs</td><td>TPMs</td><td>TPMs</td></tr>
<tr><td>Pathway Enrichment</td><td>no</td><td>yes</td><td>yes</td><td>yes</td></tr>
</table>

Supplementary Table S2. Summary of trained epigenetic age clocks. Listed are information on income and outcome variables as well as performance measures on the test set and independent dataset (Holzscheck et al., 2020). The performance measures were determined by computation of the mean absolute error (MAE) and Pearson correlation with R as correlation coefficient and p as p-value, respectively.

**clock's name**		**Wrinkle Predictor**	**Visual Facial Skin Age Clock**	**CpG-based Visual Facial Age Progression Clock**	**VisAgeX**
**income**		CpG methylation levels	CpG methylation levels	CpG methylation levels	LMR methylation levels
**outcome**		wrinkles (expert panel)	visual age (expert panel)	difference between visual age (expert panel) and chronological age	difference between visual age (expert panel) and chronological age
**Test Set**	**MAE**	8.81 wrinkle grade	6.54 years	6.26 years	6.17 years
	**R**	0.86	0.84	0.27	0.3
	**p**	1.03E-21	2.10E-20	2.30E-02	1.20E-02
**Independent Set**	**MAE**	9.66 wrinkle grade	5.76 years	6.23 years	4.67 years
	**R**	0.89	0.91	0.41	0.48
	**p**	1.27E-18	2.70E-20	2.90E-03	3.30E-04

Supplementary Table S3. Gene List Derived from CpGs in LMR for Pathway-Enrichment Analysis. A comprehensive list of genes obtained by a systematic process in which CpG sites located within the LMR are annotated with genes using the Infinium MethylationEPIC v1.0 B5 Manifest File. The resulting gene list serves as the basis for the subsequent pathway-enrichment analysis.

UCSC_RefGene_Name
A1BG
A1BG-AS1
A1CF
A2BP1
A2LD1
A2M
A2M-AS1
A2ML1
A2MP1
A4GALT
A4GNT
AA06
AAA1
AAAS
AACS
AACSL
AADAC
AADACL2-AS1
AADACL3
AADACL4
AADACP1
AADAT
AAGAB
AAK1
AAMDC
AAMP
AANAT
AARD
AARS
AARS2
AARSD1
AASDH
AASDHPPT
AASS
AATBC
AATF
AATK
AATK-AS1
ABAT
ABCA1
ABCA10
ABCA11P
ABCA12
ABCA13
ABCA17P
ABCA2
ABCA3
ABCA4
ABCA6

ABCA7
ABCA8
ABCA9
ABCB1
ABCB10
ABCB11
ABCB4
ABCB5
ABCB6
ABCB7
ABCB8
ABCB9
ABCC1
ABCC10
ABCC11
ABCC12
ABCC13
ABCC2
ABCC3
ABCC4
ABCC5
ABCC5-AS1
ABCC6
ABCC6P1
ABCC6P2
ABCC8
ABCC9
ABCD1
ABCD2
ABCD3
ABCD4
ABCE1
ABCF1
ABCF2
ABCF3
ABCG1
ABCG2
ABCG4
ABCG5
ABCG8
ABHD1
ABHD10
ABHD12
ABHD12B
ABHD13
ABHD14A
ABHD14B
ABHD15
ABHD16A
ABHD16B

ABHD17B
ABHD2
ABHD3
ABHD4
ABHD5
ABHD6
ABHD8
ABI1
ABI2
ABI3
ABI3BP
ABL1
ABL2
ABLIM1
ABLIM2
ABLIM3
ABO
ABP1
ABR
ABRA
ABRACL
ABT1
ABTB1
ABTB2
ACAA1
ACAA2
ACACA
ACACB
ACAD10
ACAD8
ACAD9
ACADM
ACADS
ACADVL
ACAN
ACAP1
ACAP2
ACAP3
ACAT1
ACAT2
ACBD3
ACBD4
ACBD5
ACBD6
ACBD7
ACCN1
ACCN2
ACCN3
ACCN4
ACCN5

ACCS
ACCSL
ACD
ACE
ACE2
ACER1
ACER2
ACER3
ACHE
ACIN1
ACKR2
ACKR3
ACKR4
ACLY
ACMSD
ACN9
ACO1
ACO2
ACOT1
ACOT11
ACOT12
ACOT13
ACOT2
ACOT4
ACOT6
ACOT7
ACOT8
ACOT9
ACOX1
ACOX2
ACOX3
ACOXL
ACP1
ACP2
ACP5
ACP6
ACPL2
ACPP
ACPT
ACR
ACRBP
ACRC
ACRV1
ACSBG1
ACSBG2
ACSF2
ACSF3
ACSL1
ACSL3
ACSL4

ACSL5
ACSL6
ACSM1
ACSM3
ACSM4
ACSM5
ACSM6
ACSS1
ACSS2
ACSS3
ACTA1
ACTA2
ACTA2-AS1
ACTB
ACTBL2
ACTC1
ACTG1
ACTG1P17
ACTG2
ACTL10
ACTL6A
ACTL6B
ACTL8
ACTL9
ACTN1
ACTN2
ACTN3
ACTN4
ACTR10
ACTR1A
ACTR1B
ACTR2
ACTR3
ACTR3B
ACTR3C
ACTR5
ACTR6
ACTR8
ACTRT1
ACTRT2
ACVR1
ACVR1B
ACVR1C
ACVR2A
ACVR2B
ACVRL1
ACY1
ACY3
ACYP1
ACYP2

ADA
ADAD1
ADAD2
ADAL
ADAM10
ADAM11
ADAM12
ADAM15
ADAM17
ADAM18
ADAM19
ADAM2
ADAM20
ADAM21
ADAM21P1
ADAM22
ADAM23
ADAM28
ADAM29
ADAM30
ADAM32
ADAM33
ADAM5P
ADAM6
ADAM7
ADAM8
ADAM9
ADAMDEC1
ADAMTS1
ADAMTS10
ADAMTS12
ADAMTS13
ADAMTS14
ADAMTS15
ADAMTS16
ADAMTS17
ADAMTS18
ADAMTS19
ADAMTS19-AS1
ADAMTS2
ADAMTS20
ADAMTS3
ADAMTS4
ADAMTS5
ADAMTS6
ADAMTS7
ADAMTS8
ADAMTS9
ADAMTS9-AS1
ADAMTS9-AS2

ADAMTSL1
ADAMTSL2
ADAMTSL3
ADAMTSL4
ADAMTSL5
ADAP1
ADAP2
ADAR
ADARB1
ADARB2
ADAT1
ADAT2
ADAT3
ADC
ADCK1
ADCK2
ADCK3
ADCK4
ADCK5
ADCY1
ADCY10
ADCY10P1
ADCY2
ADCY3
ADCY4
ADCY5
ADCY6
ADCY7
ADCY8
ADCY9
ADCYAP1
ADCYAP1R1
ADD1
ADD2
ADD3
ADD3-AS1
ADGB
ADGRA1
ADGRA2
ADGRA3
ADGRB1
ADGRB2
ADGRB3
ADGRD1
ADGRE1
ADGRE2
ADGRE3
ADGRE5
ADGRF1
ADGRF2

ADGRF3
ADGRF4
ADGRF5
ADGRG1
ADGRG2
ADGRG3
ADGRG4
ADGRG5
ADGRG6
ADGRG7
ADGRL1
ADGRV1
ADH1B
ADH5
ADH7
ADHFE1
ADI1
ADIG
ADIPOQ
ADIPOR1
ADIPOR2
ADK
ADM
ADM2
ADNP
ADNP2
ADO
ADORA1
ADORA2A
ADORA2A-AS1
ADORA2B
ADORA3
ADPGK
ADPGK-AS1
ADPRH
ADPRHL1
ADPRHL2
ADRA1A
ADRA1B
ADRA1D
ADRA2A
ADRA2B
ADRA2C
ADRB1
ADRB2
ADRB3
ADRBK1
ADRBK2
ADRM1
ADSL

ADSS
ADSSL1
ADTRP
AEBP1
AEBP2
AEN
AES
AFAP1
AFAP1L1
AFAP1L2
AFF1
AFF2
AFF3
AFF4
AFG3L1
AFG3L1P
AFG3L2
AFM
AFMID
AFP
AFTPH
AGA
AGAP1
AGAP1-IT1
AGAP11
AGAP2
AGAP3
AGAP4
AGAP5
AGAP6
AGAP7P
AGAP8
AGBL1
AGBL1-AS1
AGBL2
AGBL3
AGBL4
AGBL4-IT1
AGBL5
AGER
AGFG1
AGFG2
AGGF1
AGK
AGL
AGMAT
AGMO
AGO2
AGO3
AGO4

AGPAT1
AGPAT2
AGPAT3
AGPAT4
AGPAT5
AGPAT6
AGPAT9
AGPHD1
AGPS
AGR2
AGRN
AGT
AGTPBP1
AGTR1
AGTRAP
AGXT
AGXT2
AGXT2L1
AGXT2L2
AHCTF1
AHCTF1P1
AHCY
AHCYL1
AHCYL2
AHDC1
AHI1
AHNAK
AHNAK2
AHR
AHRR
AHSA1
AHSA2
AHSG
AICDA
AIF1
AIF1L
AIFM1
AIFM2
AIFM3
AIG1
AIM1
AIM1L
AIM2
AIMP1
AIMP2
AIP
AIPL1
AIRE
AJAP1
AJUBA

AK1
AK2
AK3
AK3L1
AK4
AK5
AK7
AK8
AK9
AKAP1
AKAP10
AKAP11
AKAP12
AKAP13
AKAP14
AKAP2
AKAP3
AKAP4
AKAP5
AKAP6
AKAP7
AKAP8
AKAP8L
AKAP9
AKD1
AKIP1
AKIRIN1
AKNA
AKNAD1
AKR1A1
AKR1B1
AKR1B10
AKR1B15
AKR1C1
AKR1C2
AKR1C3
AKR1C4
AKR1CL1
AKR1D1
AKR1E2
AKR7A2
AKR7A3
AKR7L
AKT1
AKT1S1
AKT2
AKT3
AKTIP
ALAD
ALAS1

ALAS2
ALB
ALCAM
ALDH16A1
ALDH18A1
ALDH1A1
ALDH1A2
ALDH1A3
ALDH1B1
ALDH1L1
ALDH1L1-AS2
ALDH1L2
ALDH2
ALDH3A1
ALDH3A2
ALDH3B1
ALDH3B2
ALDH4A1
ALDH5A1
ALDH6A1
ALDH7A1
ALDH8A1
ALDH9A1
ALDOA
ALDOB
ALDOC
ALG1
ALG10B
ALG11
ALG12
ALG13
ALG14
ALG1L9P
ALG2
ALG3
ALG5
ALG6
ALG8
ALG9
ALK
ALKBH1
ALKBH2
ALKBH3
ALKBH3-AS1
ALKBH4
ALKBH5
ALKBH6
ALKBH7
ALKBH8
ALLC

ALMS1
ALMS1P
ALOX12
ALOX12B
ALOX12P2
ALOX15
ALOX15B
ALOX5
ALOX5AP
ALOXE3
ALPI
ALPK1
ALPK2
ALPK3
ALPL
ALPP
ALPPL2
ALS2
ALS2CL
ALS2CR11
ALS2CR12
ALS2CR4
ALX1
ALX3
ALX4
ALYREF
AMAC1L3
AMACR
AMBN
AMBP
AMBRA1
AMD1
AMDHD1
AMDHD2
AMER2
AMER3
AMFR
AMH
AMHR2
AMICA1
AMIGO2
AMIGO3
AMMECR1
AMMECR1L
AMN
AMN1
AMOT
AMOTL1
AMOTL2
AMPD1

AMPD2
AMPD3
AMPH
AMT
AMTN
AMY2A
AMY2B
AMZ1
AMZ2P1
ANAPC1
ANAPC10
ANAPC11
ANAPC13
ANAPC15
ANAPC16
ANAPC1P1
ANAPC2
ANAPC5
ANAPC7
ANG
ANGEL1
ANGEL2
ANGPT1
ANGPT2
ANGPT4
ANGPTL2
ANGPTL4
ANGPTL5
ANGPTL6
ANGPTL7
ANK1
ANK2
ANK3
ANKAR
ANKDD1A
ANKDD1B
ANKEF1
ANKFN1
ANKFY1
ANKH
ANKHD1
ANKIB1
ANKK1
ANKLE1
ANKLE2
ANKMY1
ANKMY2
ANKRA2
ANKRD10
ANKRD11

ANKRD12
ANKRD13A
ANKRD13B
ANKRD13D
ANKRD16
ANKRD17
ANKRD18A
ANKRD18DP
ANKRD19
ANKRD19P
ANKRD2
ANKRD20A1
ANKRD20A3
ANKRD20A5P
ANKRD20A9P
ANKRD20B
ANKRD22
ANKRD23
ANKRD24
ANKRD26
ANKRD27
ANKRD28
ANKRD29
ANKRD30B
ANKRD30BP3
ANKRD31
ANKRD32
ANKRD33
ANKRD33B
ANKRD34A
ANKRD34B
ANKRD34C
ANKRD35
ANKRD36
ANKRD36C
ANKRD37
ANKRD39
ANKRD40
ANKRD42
ANKRD43
ANKRD44
ANKRD44-IT1
ANKRD45
ANKRD46
ANKRD49
ANKRD5
ANKRD50
ANKRD52
ANKRD53
ANKRD54

ANKRD55
ANKRD56
ANKRD58
ANKRD6
ANKRD60
ANKRD61
ANKRD62
ANKRD63
ANKRD66
ANKRD9
ANKS1A
ANKS1B
ANKS3
ANKS4B
ANKS6
ANKUB1
ANKZF1
ANLN
ANO1
ANO1-AS2
ANO10
ANO2
ANO3
ANO4
ANO5
ANO6
ANO7
ANO8
ANO9
ANOS1
ANP32A
ANP32B
ANP32D
ANP32E
ANPEP
ANTXR1
ANTXR2
ANTXRL
ANTXRLP1
ANUBL1
ANXA1
ANXA10
ANXA11
ANXA13
ANXA2
ANXA2P2
ANXA3
ANXA4
ANXA5
ANXA6

ANXA7
ANXA8L1
ANXA8L2
ANXA9
AOAH
AOC1
AOC2
AOC3
AOX1
AOX2P
AP1AR
AP1B1
AP1B1P1
AP1G1
AP1G2
AP1M1
AP1S1
AP1S2
AP1S3
AP2A1
AP2A2
AP2B1
AP2M1
AP2S1
AP3B1
AP3B2
AP3D1
AP3M1
AP3M2
AP3S1
AP3S2
AP4B1
AP4B1-AS1
AP4E1
AP4M1
AP4S1
AP5B1
AP5Z1
APAF1
APBA1
APBA2
APBA3
APBB1
APBB1IP
APBB2
APBB3
APC
APC2
APCDD1
APCDD1L

APCDD1L-AS1
APCS
APEH
APELA
APEX2
APH1A
APH1B
API5
APIP
APITD1
APITD1-CORT
APLF
APLN
APLP1
APLP2
APMAP
APOA1
APOA1BP
APOA4
APOA5
APOB
APOB48R
APOBEC1
APOBEC2
APOBEC3A
APOBEC3A_B
APOBEC3B
APOBEC3C
APOBEC3D
APOBEC3F
APOBEC3G
APOBEC3H
APOBEC4
APOC1
APOC1P1
APOC2
APOC3
APOC4
APOC4-APOC2
APOD
APOE
APOH
APOL1
APOL2
APOL3
APOL5
APOL6
APOLD1
APOM
APOO

APOOP5
APOPT1
APP
APPBP2
APPL1
APPL2
APRT
APTX
AQP1
AQP10
AQP11
AQP12A
AQP12B
AQP2
AQP4
AQP5
AQP6
AQP7
AQP7P1
AQP8
AQP9
AR
ARAF
ARAP1
ARAP2
ARAP3
ARC
ARCN1
AREG
AREL1
ARF1
ARF3
ARF4
ARF5
ARF6
ARFGAP1
ARFGAP2
ARFGAP3
ARFGEF1
ARFGEF2
ARFGEF3
ARFIP1
ARFIP2
ARFRP1
ARG1
ARG2
ARGFX
ARGLU1
ARHGAP1
ARHGAP10

ARHGAP11A
ARHGAP12
ARHGAP15
ARHGAP17
ARHGAP18
ARHGAP19
ARHGAP20
ARHGAP21
ARHGAP22
ARHGAP23
ARHGAP24
ARHGAP25
ARHGAP26
ARHGAP26-IT1
ARHGAP27
ARHGAP28
ARHGAP29
ARHGAP30
ARHGAP31
ARHGAP32
ARHGAP33
ARHGAP35
ARHGAP36
ARHGAP39
ARHGAP4
ARHGAP40
ARHGAP42
ARHGAP44
ARHGAP6
ARHGAP8
ARHGAP9
ARHGDIA
ARHGDIB
ARHGDIG
ARHGEF1
ARHGEF10
ARHGEF10L
ARHGEF11
ARHGEF12
ARHGEF15
ARHGEF16
ARHGEF17
ARHGEF18
ARHGEF19
ARHGEF2
ARHGEF26
ARHGEF28
ARHGEF3
ARHGEF37
ARHGEF38

ARHGEF4
ARHGEF5L
ARHGEF6
ARHGEF7
ARHGEF9
ARHGEF9-IT1
ARID1A
ARID1B
ARID2
ARID3A
ARID3B
ARID3C
ARID4B
ARID5A
ARID5B
ARIH1
ARIH2
ARL1
ARL10
ARL13A
ARL13B
ARL14
ARL14EP
ARL15
ARL16
ARL17A
ARL2
ARL2BP
ARL3
ARL4A
ARL4C
ARL4D
ARL5A
ARL5B
ARL5C
ARL6
ARL6IP1
ARL6IP4
ARL6IP5
ARL6IP6
ARL8A
ARL8B
ARL9
ARMC1
ARMC10
ARMC12
ARMC2
ARMC3
ARMC4
ARMC5

ARMC6
ARMC7
ARMC8
ARMC9
ARMCX1
ARMCX2
ARMCX3
ARMCX4
ARMCX5
ARMCX5-GPRASP2
ARMCX6
ARNT
ARNT2
ARNTL
ARNTL2
ARNTL2-AS1
ARPC1A
ARPC1B
ARPC2
ARPC3
ARPC4
ARPC4-TTLL3
ARPC5
ARPC5L
ARPIN
ARPM1
ARPP-21
ARPP19
ARPP21
ARRB1
ARRB2
ARRDC1
ARRDC1-AS1
ARRDC2
ARRDC3
ARRDC4
ARRDC5
ARSA
ARSB
ARSD
ARSE
ARSF
ARSG
ARSH
ARSI
ARSJ
ARSK
ART1
ART3
ART4

ART5
ARV1
ARVCF
ARX
AS3MT
ASAH1
ASAH2B
ASAM
ASAP1
ASAP1-IT2
ASAP2
ASAP3
ASB1
ASB10
ASB11
ASB13
ASB14
ASB15
ASB16
ASB18
ASB2
ASB4
ASB5
ASB6
ASB7
ASB8
ASB9
ASCC1
ASCC2
ASCC3
ASCL1
ASCL2
ASCL3
ASCL4
ASCL5
ASF1A
ASF1B
ASFMR1
ASGR1
ASGR2
ASH1L
ASH2L
ASIC1
ASIC2
ASIC3
ASIC4
ASIP
ASL
ASNA1
ASNS

ASNSD1
ASPA
ASPDH
ASPG
ASPH
ASPHD1
ASPHD2
ASPM
ASPRV1
ASPSCR1
ASRGL1
ASS1
ASTE1
ASTL
ASTN1
ASTN2
ASUN
ASXL1
ASXL2
ASXL3
ASZ1
ATAD1
ATAD2
ATAD2B
ATAD3A
ATAD3B
ATAD3C
ATAT1
ATCAY
ATE1
ATF1
ATF3
ATF4
ATF5
ATF6
ATF6B
ATF7
ATF7IP
ATF7IP2
ATG10
ATG101
ATG12
ATG13
ATG14
ATG16L1
ATG16L2
ATG2A
ATG2B
ATG3
ATG4A

ATG4B
ATG4C
ATG4D
ATG5
ATG7
ATG9A
ATG9B
ATHL1
ATIC
ATL1
ATL2
ATL3
ATM
ATMIN
ATN1
ATOH1
ATOH7
ATOH8
ATOX1
ATP10A
ATP10B
ATP10D
ATP11A
ATP11C
ATP12A
ATP13A1
ATP13A2
ATP13A3
ATP13A4
ATP13A4-AS1
ATP13A5
ATP13A5-AS1
ATP1A1
ATP1A1-AS1
ATP1A2
ATP1A3
ATP1A4
ATP1B1
ATP1B2
ATP1B3
ATP1B4
ATP2A1
ATP2A1-AS1
ATP2A2
ATP2A3
ATP2B1
ATP2B2
ATP2B3
ATP2B4
ATP2C1

ATP2C2
ATP4A
ATP4B
ATP5A1
ATP5B
ATP5C1
ATP5D
ATP5E
ATP5F1
ATP5G1
ATP5G2
ATP5G3
ATP5H
ATP5I
ATP5J
ATP5J2
ATP5J2-PTCD1
ATP5L
ATP5O
ATP5S
ATP5SL
ATP6AP1
ATP6AP1L
ATP6AP2
ATP6V0A1
ATP6V0A2
ATP6V0A4
ATP6V0B
ATP6V0C
ATP6V0D1
ATP6V0D2
ATP6V0E1
ATP6V0E2
ATP6V1A
ATP6V1B1
ATP6V1B1-AS1
ATP6V1B2
ATP6V1C1
ATP6V1C2
ATP6V1D
ATP6V1E1
ATP6V1E2
ATP6V1F
ATP6V1G1
ATP6V1G2
ATP6V1G3
ATP6V1H
ATP7A
ATP7B
ATP8A1

ATP8A2
ATP8B1
ATP8B2
ATP8B3
ATP8B4
ATP8B5P
ATP9A
ATP9B
ATPAF1
ATPAF2
ATPBD4
ATPGD1
ATR
ATRIP
ATRN
ATRNL1
ATRX
ATXN1
ATXN10
ATXN1L
ATXN2
ATXN2L
ATXN3
ATXN7
ATXN7L1
ATXN7L2
ATXN7L3
AUH
AUNIP
AUP1
AURKA
AURKAIP1
AURKB
AURKC
AUTS2
AVEN
AVIL
AVL9
AVP
AVPI1
AVPR1A
AVPR1B
AVPR2
AWAT1
AXDND1
AXIN1
AXIN2
AXL
AZGP1
AZI1

AZI2
AZIN1
AZIN1-AS1
AZIN2
AZU1
B2M
B3GALNT1
B3GALNT2
B3GALT1
B3GALT4
B3GALT5
B3GALT5-AS1
B3GALT6
B3GALTL
B3GAT1
B3GAT2
B3GAT3
B3GLCT
B3GNT1
B3GNT2
B3GNT3
B3GNT4
B3GNT6
B3GNT7
B3GNT8
B3GNT9
B3GNTL1
B4GALNT1
B4GALNT2
B4GALNT3
B4GALNT4
B4GALT1
B4GALT1-AS1
B4GALT2
B4GALT3
B4GALT4
B4GALT4-AS1
B4GALT5
B4GALT6
B4GALT7
B9D1
B9D2
BAALC
BAALC-AS2
BABAM1
BACE1
BACE2
BACH1
BACH1-IT2
BACH2

BAD
BAG1
BAG2
BAG3
BAG4
BAG5
BAGE3
BAGE4
BAHCC1
BAHD1
BAI1
BAI2
BAI3
BAIAP2
BAIAP2L1
BAIAP2L2
BAIAP3
BAK1
BAMBI
BANF1
BANF2
BANK1
BANP
BAP1
BARD1
BARHL1
BARHL2
BARX1
BARX2
BASE
BASP1
BAT1
BAT2
BAT2L1
BAT2L2
BAT3
BAT4
BAT5
BATF
BATF2
BATF3
BAX
BAZ1A
BAZ1B
BAZ2A
BAZ2B
BBC3
BBIP1
BBOX1-AS1
BBS1

BBS10
BBS2
BBS4
BBS5
BBS7
BBS9
BBX
BCAM
BCAN
BCAP29
BCAP31
BCAR1
BCAR3
BCAS1
BCAS2
BCAS3
BCAS4
BCAT1
BCAT2
BCCIP
BCDIN3D
BCDIN3D-AS1
BCHE
BCKDHA
BCKDHB
BCKDK
BCL10
BCL11A
BCL11B
BCL2
BCL2L1
BCL2L10
BCL2L11
BCL2L12
BCL2L13
BCL2L14
BCL2L15
BCL2L2
BCL2L2-PABPN1
BCL3
BCL6
BCL6B
BCL7A
BCL7B
BCL7C
BCL9
BCL9L
BCLAF1
BCO1
BCO2

BCOR
BCORL1
BCR
BCRP2
BCRP3
BCS1L
BCYRN1
BDH1
BDH2
BDKRB1
BDKRB2
BDNF
BDNF-AS
BDNFOS
BDP1
BEAN
BEAN1
BEAN1-AS1
BECN2
BEGAIN
BEND2
BEND3
BEND4
BEND5
BEND6
BEND7
BEST1
BEST2
BEST3
BEST4
BET1
BET1L
BET3L
BEX1
BEX2
BEX4
BEX5
BEYLA
BFAR
BFSP1
BFSP2
BGLAP
BGN
BHLHA15
BHLHA9
BHLHB9
BHLHE22
BHLHE23
BHLHE40
BHLHE40-AS1

BHLHE41
BHMG1
BHMT
BHMT2
BICC1
BICD1
BICD2
BID
BIK
BIN1
BIN2
BIN3
BIRC2
BIRC3
BIRC5
BIRC6
BIRC6-AS2
BIRC7
BIRC8
BISPR
BIVM
BIVM-ERCC5
BLACAT1
BLACE
BLCAP
BLID
BLK
BLM
BLMH
BLNK
BLOC1S1
BLOC1S2
BLOC1S3
BLOC1S5-TXNDC5
BLOC1S6
BLVRA
BLVRB
BLZF1
BMF
BMI1
BMP1
BMP10
BMP15
BMP2
BMP2K
BMP3
BMP4
BMP5
BMP6
BMP7

BMP7-AS1
BMP8A
BMP8B
BMPER
BMPR1A
BMPR1B
BMPR1B-AS1
BMPR2
BMS1
BMS1P1
BMS1P20
BMS1P4
BMX
BNC1
BNC2
BNIP1
BNIP2
BNIP3
BNIP3L
BNIPL
BOC
BOD1
BOD1L1
BOD1L2
BOK
BOK-AS1
BOLA1
BOLA2
BOLA3
BOLA3-AS1
BOLL
BOP1
BPESC1
BPGM
BPHL
BPI
BPIFA1
BPIFA2
BPIFA3
BPIFA4P
BPIFB1
BPIFB2
BPIFB3
BPIFB4
BPIFB6
BPIFC
BPIL1
BPIL2
BPIL3
BPNT1

BPTF
BRAF
BRAP
BRAT1
BRCA1
BRCA2
BRCAT107
BRCAT54
BRCC3
BRD1
BRD2
BRD3
BRD4
BRD7
BRD8
BRD9
BRDT
BRE
BREA2
BRF1
BRF2
BRI3
BRICD5
BRINP1
BRINP2
BRINP3
BRIP1
BRK1
BRMS1
BRMS1L
BROX
BRP44
BRP44L
BRPF1
BRPF3
BRSK1
BRSK2
BRUNOL4
BRUNOL5
BRUNOL6
BRWD1
BRWD1-AS1
BRWD1-IT2
BRWD3
BSCL2
BSDC1
BSG
BSN
BSN-AS2
BSPRY

BST1
BST2
BSX
BTAF1
BTBD1
BTBD10
BTBD11
BTBD12
BTBD16
BTBD17
BTBD18
BTBD19
BTBD2
BTBD3
BTBD6
BTBD7
BTBD8
BTBD9
BTD
BTF3
BTF3L4
BTG1
BTG2
BTG3
BTG4
BTLA
BTN1A1
BTN2A1
BTN2A2
BTN3A1
BTN3A2
BTN3A3
BTNL2
BTNL3
BTNL8
BTNL9
BTRC
BUB1
BUB1B
BUB3
BUD13
BUD31
BVES
BVES-AS1
BYSL
BZRAP1
BZRAP1-AS1
BZW1
BZW2
C10orf10

C10orf105
C10orf107
C10orf11
C10orf110
C10orf111
C10orf113
C10orf114
C10orf116
C10orf118
C10orf120
C10orf122
C10orf125
C10orf126
C10orf128
C10orf131
C10orf137
C10orf140
C10orf142
C10orf18
C10orf2
C10orf25
C10orf26
C10orf27
C10orf28
C10orf32
C10orf35
C10orf4
C10orf41
C10orf46
C10orf47
C10orf50
C10orf53
C10orf54
C10orf55
C10orf57
C10orf58
C10orf67
C10orf71
C10orf72
C10orf75
C10orf76
C10orf78
C10orf79
C10orf81
C10orf82
C10orf84
C10orf88
C10orf90
C10orf93
C10orf96

C10orf99
C11orf1
C11orf16
C11orf17
C11orf2
C11orf20
C11orf21
C11orf24
C11orf30
C11orf31
C11orf34
C11orf35
C11orf36
C11orf39
C11orf40
C11orf41
C11orf44
C11orf46
C11orf48
C11orf49
C11orf51
C11orf52
C11orf53
C11orf57
C11orf58
C11orf60
C11orf61
C11orf63
C11orf64
C11orf65
C11orf66
C11orf67
C11orf68
C11orf70
C11orf71
C11orf73
C11orf74
C11orf75
C11orf80
C11orf82
C11orf83
C11orf85
C11orf86
C11orf87
C11orf88
C11orf9
C11orf90
C11orf91
C11orf92
C11orf93

C11orf94
C11orf95
C11orf97
C12orf10
C12orf11
C12orf12
C12orf26
C12orf29
C12orf34
C12orf35
C12orf39
C12orf4
C12orf40
C12orf41
C12orf42
C12orf43
C12orf44
C12orf45
C12orf47
C12orf49
C12orf5
C12orf51
C12orf52
C12orf53
C12orf54
C12orf56
C12orf57
C12orf60
C12orf61
C12orf62
C12orf65
C12orf66
C12orf67
C12orf68
C12orf69
C12orf70
C12orf72
C12orf73
C12orf74
C12orf75
C12orf76
C12orf77
C12orf79
C12orf80
C13orf1
C13orf15
C13orf16
C13orf18
C13orf23
C13orf26

C13orf28
C13orf29
C13orf30
C13orf33
C13orf34
C13orf35
C13orf36
C13orf38
C13orf39
C14orf1
C14orf101
C14orf102
C14orf104
C14orf105
C14orf106
C14orf109
C14orf115
C14orf118
C14orf119
C14orf132
C14orf133
C14orf135
C14orf138
C14orf139
C14orf142
C14orf143
C14orf145
C14orf148
C14orf149
C14orf153
C14orf156
C14orf159
C14orf165
C14orf166B
C14orf167
C14orf169
C14orf178
C14orf179
C14orf180
C14orf181
C14orf182
C14orf183
C14orf184
C14orf19
C14orf2
C14orf23
C14orf28
C14orf33
C14orf37
C14orf38

C14orf39
C14orf4
C14orf43
C14orf45
C14orf48
C14orf49
C14orf50
C14orf64
C14orf68
C14orf70
C14orf73
C14orf79
C14orf80
C14orf86
C14orf93
C15orf17
C15orf2
C15orf23
C15orf24
C15orf26
C15orf27
C15orf28
C15orf29
C15orf32
C15orf37
C15orf38
C15orf39
C15orf40
C15orf41
C15orf42
C15orf43
C15orf44
C15orf48
C15orf50
C15orf52
C15orf54
C15orf55
C15orf56
C15orf57
C15orf58
C15orf59
C15orf60
C15orf61
C15orf62
C15orf65
C16orf11
C16orf13
C16orf3
C16orf35
C16orf42

C16orf45
C16orf46
C16orf47
C16orf5
C16orf52
C16orf53
C16orf54
C16orf55
C16orf57
C16orf58
C16orf59
C16orf62
C16orf63
C16orf65
C16orf68
C16orf7
C16orf70
C16orf71
C16orf72
C16orf73
C16orf74
C16orf78
C16orf80
C16orf81
C16orf82
C16orf86
C16orf87
C16orf88
C16orf89
C16orf91
C16orf92
C16orf95
C16orf96
C16orf97
C17orf100
C17orf101
C17orf102
C17orf103
C17orf104
C17orf105
C17orf106
C17orf108
C17orf112
C17orf28
C17orf37
C17orf42
C17orf44
C17orf47
C17orf48
C17orf50

C17orf51
C17orf53
C17orf54
C17orf55
C17orf56
C17orf57
C17orf59
C17orf62
C17orf63
C17orf64
C17orf65
C17orf67
C17orf68
C17orf69
C17orf70
C17orf73
C17orf75
C17orf76
C17orf77
C17orf81
C17orf82
C17orf85
C17orf88
C17orf90
C17orf91
C17orf93
C17orf95
C17orf96
C17orf97
C17orf98
C17orf99
C18orf1
C18orf10
C18orf18
C18orf19
C18orf2
C18orf20
C18orf21
C18orf22
C18orf25
C18orf32
C18orf34
C18orf42
C18orf45
C18orf54
C18orf61
C18orf62
C18orf63
C18orf65
C18orf8

C19orf10
C19orf12
C19orf18
C19orf2
C19orf20
C19orf21
C19orf22
C19orf23
C19orf24
C19orf25
C19orf26
C19orf28
C19orf29
C19orf34
C19orf35
C19orf36
C19orf38
C19orf39
C19orf40
C19orf41
C19orf42
C19orf43
C19orf44
C19orf45
C19orf46
C19orf47
C19orf48
C19orf50
C19orf51
C19orf52
C19orf53
C19orf54
C19orf55
C19orf56
C19orf57
C19orf59
C19orf6
C19orf60
C19orf61
C19orf62
C19orf63
C19orf66
C19orf67
C19orf68
C19orf69
C19orf75
C19orf77
C19orf80
C19orf81
C19orf84

C1D
C1GALT1
C1GALT1C1
C1orf100
C1orf101
C1orf103
C1orf105
C1orf106
C1orf107
C1orf110
C1orf112
C1orf113
C1orf114
C1orf115
C1orf116
C1orf122
C1orf123
C1orf124
C1orf125
C1orf127
C1orf128
C1orf130
C1orf131
C1orf133
C1orf135
C1orf137
C1orf14
C1orf141
C1orf146
C1orf150
C1orf151
C1orf157
C1orf158
C1orf159
C1orf161
C1orf162
C1orf163
C1orf167
C1orf168
C1orf170
C1orf172
C1orf173
C1orf174
C1orf175
C1orf177
C1orf180
C1orf183
C1orf185
C1orf186
C1orf187

C1orf189
C1orf194
C1orf195
C1orf198
C1orf200
C1orf201
C1orf203
C1orf204
C1orf21
C1orf213
C1orf216
C1orf220
C1orf226
C1orf227
C1orf228
C1orf229
C1orf230
C1orf234
C1orf25
C1orf26
C1orf27
C1orf31
C1orf35
C1orf38
C1orf43
C1orf50
C1orf51
C1orf52
C1orf53
C1orf54
C1orf55
C1orf56
C1orf57
C1orf59
C1orf61
C1orf63
C1orf64
C1orf65
C1orf66
C1orf69
C1orf70
C1orf74
C1orf77
C1orf83
C1orf84
C1orf85
C1orf86
C1orf87
C1orf88
C1orf89

C1orf9
C1orf91
C1orf92
C1orf93
C1orf94
C1orf95
C1orf96
C1orf97
C1QA
C1QB
C1QBP
C1QC
C1QL1
C1QL2
C1QL3
C1QL4
C1QTNF1
C1QTNF2
C1QTNF3
C1QTNF4
C1QTNF5
C1QTNF6
C1QTNF7
C1QTNF8
C1QTNF9
C1QTNF9B
C1QTNF9B-AS1
C1R
C1RL
C1RL-AS1
C2
C20orf103
C20orf108
C20orf111
C20orf112
C20orf117
C20orf118
C20orf12
C20orf123
C20orf132
C20orf135
C20orf141
C20orf151
C20orf152
C20orf160
C20orf166
C20orf173
C20orf177
C20orf185
C20orf186

C20orf194
C20orf195
C20orf196
C20orf197
C20orf199
C20orf202
C20orf203
C20orf24
C20orf26
C20orf27
C20orf29
C20orf30
C20orf4
C20orf43
C20orf46
C20orf54
C20orf56
C20orf62
C20orf7
C20orf71
C20orf72
C20orf78
C20orf79
C20orf85
C20orf95
C20orf96
C21orf119
C21orf121
C21orf130
C21orf2
C21orf29
C21orf33
C21orf34
C21orf45
C21orf54
C21orf56
C21orf57
C21orf58
C21orf59
C21orf62
C21orf62-AS1
C21orf63
C21orf66
C21orf67
C21orf7
C21orf70
C21orf81
C21orf82
C21orf88
C21orf90

C21orf91
C21orf91-OT1
C21orf99
C22orf13
C22orf15
C22orf23
C22orf24
C22orf25
C22orf26
C22orf27
C22orf28
C22orf29
C22orf30
C22orf32
C22orf33
C22orf34
C22orf36
C22orf39
C22orf40
C22orf41
C22orf42
C22orf43
C22orf45
C22orf46
C22orf9
C2CD2
C2CD2L
C2CD3
C2CD4A
C2CD4B
C2CD4C
C2orf15
C2orf16
C2orf18
C2orf24
C2orf27A
C2orf27B
C2orf28
C2orf29
C2orf39
C2orf40
C2orf42
C2orf43
C2orf44
C2orf48
C2orf49
C2orf50
C2orf52
C2orf53
C2orf54

C2orf55
C2orf56
C2orf57
C2orf58
C2orf60
C2orf61
C2orf62
C2orf63
C2orf64
C2orf65
C2orf66
C2orf67
C2orf68
C2orf70
C2orf71
C2orf72
C2orf73
C2orf74
C2orf76
C2orf77
C2orf79
C2orf80
C2orf81
C2orf82
C2orf83
C2orf84
C2orf85
C2orf86
C2orf88
C2orf89
C2orf91
C3
C3AR1
C3orf1
C3orf15
C3orf16
C3orf17
C3orf18
C3orf20
C3orf21
C3orf22
C3orf23
C3orf24
C3orf25
C3orf26
C3orf27
C3orf30
C3orf31
C3orf32
C3orf33

C3orf35
C3orf37
C3orf38
C3orf39
C3orf43
C3orf49
C3orf50
C3orf51
C3orf52
C3orf54
C3orf55
C3orf56
C3orf57
C3orf58
C3orf59
C3orf63
C3orf64
C3orf66
C3orf67
C3orf67-AS1
C3orf70
C3orf71
C3orf72
C3orf75
C3orf77
C3orf79
C3orf84
C3P1
C4A
C4B
C4BPA
C4BPB
C4orf10
C4orf11
C4orf14
C4orf17
C4orf19
C4orf21
C4orf22
C4orf23
C4orf26
C4orf27
C4orf29
C4orf3
C4orf31
C4orf32
C4orf33
C4orf34
C4orf36
C4orf37

C4orf38
C4orf39
C4orf41
C4orf42
C4orf43
C4orf44
C4orf45
C4orf47
C4orf48
C4orf49
C4orf50
C4orf51
C4orf52
C4orf6
C4orf7
C5
C5AR1
C5AR2
C5orf13
C5orf15
C5orf20
C5orf24
C5orf25
C5orf28
C5orf30
C5orf32
C5orf33
C5orf34
C5orf35
C5orf36
C5orf37
C5orf38
C5orf39
C5orf4
C5orf41
C5orf42
C5orf44
C5orf45
C5orf46
C5orf47
C5orf48
C5orf49
C5orf51
C5orf52
C5orf53
C5orf54
C5orf56
C5orf58
C5orf60
C5orf62

C5orf64
C5orf66
C5orf66-AS1
C5orf66-AS2
C5orf67
C6
C6orf1
C6orf10
C6orf103
C6orf105
C6orf106
C6orf108
C6orf115
C6orf118
C6orf120
C6orf122
C6orf123
C6orf124
C6orf125
C6orf127
C6orf129
C6orf132
C6orf134
C6orf136
C6orf138
C6orf142
C6orf145
C6orf146
C6orf147
C6orf15
C6orf150
C6orf153
C6orf154
C6orf155
C6orf162
C6orf163
C6orf164
C6orf165
C6orf167
C6orf168
C6orf170
C6orf174
C6orf176
C6orf182
C6orf186
C6orf191
C6orf192
C6orf195
C6orf201
C6orf203

C6orf204
C6orf208
C6orf211
C6orf217
C6orf221
C6orf222
C6orf223
C6orf225
C6orf227
C6orf229
C6orf25
C6orf26
C6orf27
C6orf35
C6orf47
C6orf48
C6orf52
C6orf57
C6orf58
C6orf59
C6orf62
C6orf64
C6orf81
C6orf89
C6orf94
C6orf97
C6orf99
C7
C7orf10
C7orf13
C7orf16
C7orf20
C7orf23
C7orf25
C7orf26
C7orf27
C7orf28A
C7orf29
C7orf31
C7orf33
C7orf34
C7orf38
C7orf4
C7orf41
C7orf42
C7orf43
C7orf44
C7orf46
C7orf47
C7orf49

C7orf50
C7orf51
C7orf52
C7orf53
C7orf55
C7orf55-LUC7L2
C7orf57
C7orf58
C7orf59
C7orf60
C7orf64
C7orf68
C7orf70
C7orf71
C7orf72
C7orf73
C7orf76
C7orf77
C8A
C8B
C8G
C8orf12
C8orf22
C8orf31
C8orf33
C8orf34
C8orf37
C8orf37-AS1
C8orf38
C8orf39
C8orf4
C8orf40
C8orf41
C8orf42
C8orf44
C8orf44-SGK3
C8orf46
C8orf47
C8orf48
C8orf49
C8orf51
C8orf56
C8orf58
C8orf59
C8orf71
C8orf73
C8orf74
C8orf75
C8orf76
C8orf77

C8orf79
C8orf84
C8orf85
C8orf86
C8orf87
C8orf88
C8ORFK29
C9
C9orf100
C9orf103
C9orf106
C9orf109
C9orf11
C9orf114
C9orf116
C9orf117
C9orf119
C9orf122
C9orf123
C9orf125
C9orf129
C9orf130
C9orf131
C9orf135
C9orf139
C9orf140
C9orf142
C9orf144
C9orf150
C9orf153
C9orf156
C9orf16
C9orf163
C9orf167
C9orf169
C9orf171
C9orf173
C9orf21
C9orf23
C9orf24
C9orf25
C9orf3
C9orf30
C9orf37
C9orf4
C9orf40
C9orf41-AS1
C9orf43
C9orf44
C9orf46

C9orf47
C9orf5
C9orf50
C9orf57
C9orf62
C9orf64
C9orf66
C9orf68
C9orf7
C9orf71
C9orf72
C9orf75
C9orf78
C9orf80
C9orf82
C9orf84
C9orf85
C9orf86
C9orf89
C9orf91
C9orf92
C9orf93
C9orf96
C9orf98
CA1
CA10
CA12
CA13
CA14
CA3
CA3-AS1
CA4
CA5A
CA5B
CA5BP
CA6
CA7
CA8
CA9
CAAP1
CAB39
CAB39L
CABC1
CABIN1
CABLES1
CABLES2
CABP1
CABP2
CABP5
CABP7

CABYR
CACFD1
CACHD1
CACNA1A
CACNA1B
CACNA1C
CACNA1C-AS1
CACNA1C-AS2
CACNA1C-AS4
CACNA1C-IT3
CACNA1D
CACNA1E
CACNA1F
CACNA1G
CACNA1G-AS1
CACNA1H
CACNA1I
CACNA1S
CACNA2D1
CACNA2D2
CACNA2D3
CACNA2D4
CACNB1
CACNB2
CACNB3
CACNB4
CACNG1
CACNG2
CACNG3
CACNG4
CACNG5
CACNG6
CACNG7
CACNG8
CACUL1
CACYBP
CAD
CADM1
CADM2
CADM2-AS2
CADM3
CADM4
CADPS
CADPS2
CAGE1
CALB1
CALB2
CALCA
CALCB
CALCOCO1

CALCOCO2
CALCR
CALCRL
CALD1
CALHM1
CALHM2
CALHM3
CALM1
CALM2
CALM3
CALML3
CALML3-AS1
CALML4
CALML5
CALML6
CALN1
CALR
CALU
CALY
CAMK1
CAMK1D
CAMK1G
CAMK2A
CAMK2B
CAMK2D
CAMK2G
CAMK2N1
CAMK4
CAMKK1
CAMKK2
CAMKMT
CAMKV
CAMP
CAMSAP1
CAMSAP1L1
CAMSAP3
CAMTA1
CAMTA2
CAND1
CAND1.11
CAND2
CANT1
CANX
CAP1
CAP2
CAPG
CAPN1
CAPN10
CAPN10-AS1
CAPN11

CAPN12
CAPN13
CAPN14
CAPN15
CAPN2
CAPN3
CAPN5
CAPN6
CAPN7
CAPN8
CAPN9
CAPNS1
CAPRIN1
CAPRIN2
CAPS2
CAPSL
CAPZA1
CAPZA2
CAPZA3
CAPZB
CARD10
CARD11
CARD14
CARD18
CARD6
CARD8
CARD9
CARF
CARHSP1
CARKD
CARM1
CARNS1
CARS
CARS2
CARTPT
CASC1
CASC15
CASC16
CASC18
CASC2
CASC20
CASC21
CASC4
CASC5
CASC6
CASC8
CASD1
CASK
CASKIN1
CASKIN2

CASP1
CASP10
CASP12
CASP14
CASP2
CASP3
CASP4
CASP5
CASP6
CASP7
CASP8
CASP8AP2
CASP9
CASQ1
CASQ2
CASR
CASS4
CAST
CASZ1
CAT
CATIP
CATIP-AS1
CATSPER1
CATSPER2
CATSPER2P1
CATSPER4
CATSPERB
CATSPERD
CATSPERG
CAV1
CAV2
CAV3
CBARA1
CBFA2T2
CBFA2T3
CBFB
CBL
CBLB
CBLC
CBLL1
CBLN1
CBLN2
CBLN3
CBLN4
CBR1
CBR3
CBR4
CBS
CBWD1
CBWD2

CBWD5
CBX1
CBX2
CBX3
CBX4
CBX5
CBX6
CBX7
CBX8
CBY1
CBY3
CC2D1A
CC2D1B
CC2D2A
CC2D2B
CCAR1
CCAR2
CCAT1
CCBE1
CCBL1
CCBL2
CCBP2
CCDC101
CCDC102A
CCDC102B
CCDC103
CCDC104
CCDC105
CCDC106
CCDC107
CCDC108
CCDC109A
CCDC109B
CCDC11
CCDC110
CCDC111
CCDC112
CCDC113
CCDC114
CCDC115
CCDC116
CCDC117
CCDC12
CCDC120
CCDC121
CCDC122
CCDC124
CCDC125
CCDC126
CCDC127

CCDC129
CCDC13
CCDC13-AS1
CCDC130
CCDC132
CCDC134
CCDC136
CCDC138
CCDC14
CCDC140
CCDC141
CCDC142
CCDC144A
CCDC144B
CCDC144CP
CCDC144NL
CCDC144NL-AS1
CCDC146
CCDC147
CCDC148
CCDC148-AS1
CCDC149
CCDC15
CCDC150
CCDC151
CCDC152
CCDC154
CCDC155
CCDC157
CCDC158
CCDC159
CCDC160
CCDC162
CCDC162P
CCDC163P
CCDC166
CCDC168
CCDC17
CCDC170
CCDC171
CCDC173
CCDC175
CCDC176
CCDC177
CCDC178
CCDC179
CCDC18
CCDC180
CCDC181
CCDC182

CCDC183
CCDC19
CCDC21
CCDC22
CCDC23
CCDC24
CCDC25
CCDC26
CCDC27
CCDC28A
CCDC28B
CCDC3
CCDC30
CCDC33
CCDC34
CCDC36
CCDC37
CCDC37-AS1
CCDC38
CCDC39
CCDC40
CCDC41
CCDC42
CCDC42B
CCDC43
CCDC46
CCDC47
CCDC48
CCDC49
CCDC50
CCDC51
CCDC52
CCDC53
CCDC55
CCDC57
CCDC58
CCDC6
CCDC60
CCDC61
CCDC62
CCDC63
CCDC64
CCDC64B
CCDC66
CCDC67
CCDC68
CCDC69
CCDC7
CCDC70
CCDC71

CCDC74A
CCDC74B
CCDC75
CCDC76
CCDC77
CCDC78
CCDC79
CCDC8
CCDC80
CCDC81
CCDC82
CCDC83
CCDC84
CCDC85A
CCDC85C
CCDC86
CCDC87
CCDC88A
CCDC88B
CCDC88C
CCDC89
CCDC9
CCDC90A
CCDC90B
CCDC91
CCDC92
CCDC93
CCDC94
CCDC96
CCDC97
CCDC99
CCER2
CCHCR1
CCK
CCKAR
CCKBR
CCL1
CCL14
CCL14-CCL15
CCL15
CCL16
CCL17
CCL18
CCL2
CCL20
CCL23
CCL24
CCL25
CCL27
CCL28

CCL3
CCL4
CCL4L1
CCL5
CCL7
CCL8
CCM2
CCM2L
CCNA1
CCNA2
CCNB1
CCNB1IP1
CCNB2
CCNB3
CCNC
CCND1
CCND2
CCND2-AS1
CCND3
CCNDBP1
CCNE1
CCNE2
CCNF
CCNG1
CCNG2
CCNH
CCNI
CCNI2
CCNJ
CCNJL
CCNK
CCNL1
CCNL2
CCNO
CCNT1
CCNT2
CCNT2-AS1
CCNY
CCNYL1
CCPG1
CCR1
CCR10
CCR2
CCR3
CCR4
CCR5
CCR6
CCR7
CCR8
CCR9

CCRL2
CCRN4L
CCSAP
CCSER1
CCSER2
CCT2
CCT3
CCT4
CCT5
CCT6A
CCT6P1
CCT6P3
CCT7
CCT8
CCT8L2
CCZ1
CD101
CD109
CD14
CD151
CD160
CD163
CD163L1
CD164
CD164L2
CD177
CD180
CD19
CD1A
CD1B
CD1C
CD1D
CD1E
CD2
CD200
CD200R1
CD200R1L
CD209
CD22
CD226
CD24
CD244
CD247
CD248
CD27
CD27-AS1
CD274
CD276
CD28
CD2AP

CD2BP2
CD300A
CD300C
CD300E
CD300LB
CD300LD
CD300LF
CD300LG
CD302
CD320
CD34
CD36
CD37
CD38
CD3D
CD3E
CD3EAP
CD3G
CD4
CD40
CD40LG
CD44
CD46
CD47
CD48
CD5
CD52
CD53
CD55
CD58
CD59
CD5L
CD6
CD63
CD68
CD7
CD72
CD74
CD79A
CD79B
CD80
CD81
CD81-AS1
CD82
CD83
CD84
CD86
CD8A
CD8B
CD9

CD93
CD96
CD97
CD99L2
CDA
CDADC1
CDAN1
CDC10L
CDC123
CDC14A
CDC14B
CDC14C
CDC16
CDC20
CDC20B
CDC23
CDC25A
CDC25B
CDC25C
CDC26
CDC27
CDC34
CDC37
CDC37L1
CDC37L1-AS1
CDC40
CDC42
CDC42BPA
CDC42BPB
CDC42BPG
CDC42EP1
CDC42EP2
CDC42EP3
CDC42EP4
CDC42EP5
CDC42SE1
CDC42SE2
CDC45
CDC5L
CDC6
CDC7
CDC73
CDCA2
CDCA4
CDCA5
CDCA7
CDCA7L
CDCA8
CDCP1
CDCP2

CDGAP
CDH1
CDH10
CDH11
CDH12
CDH13
CDH15
CDH16
CDH17
CDH18
CDH2
CDH20
CDH22
CDH23
CDH24
CDH26
CDH3
CDH4
CDH5
CDH6
CDH7
CDH8
CDH9
CDHR1
CDHR2
CDHR3
CDHR4
CDIP1
CDIPT
CDIPT-AS1
CDK1
CDK10
CDK11A
CDK11B
CDK12
CDK13
CDK14
CDK15
CDK16
CDK17
CDK18
CDK19
CDK2
CDK20
CDK2AP1
CDK2AP2
CDK3
CDK4
CDK5
CDK5R1

CDK5R2
CDK5RAP1
CDK5RAP2
CDK5RAP3
CDK6
CDK7
CDK8
CDK9
CDKAL1
CDKL1
CDKL2
CDKL3
CDKL4
CDKL5
CDKN1A
CDKN1B
CDKN1C
CDKN2A
CDKN2A-AS1
CDKN2AIP
CDKN2AIPNL
CDKN2B
CDKN2B-AS1
CDKN2BAS
CDKN2C
CDKN3
CDO1
CDON
CDPF1
CDR1
CDR2
CDR2L
CDRT1
CDRT15P
CDRT4
CDRT7
CDRT8
CDS1
CDS2
CDSN
CDT1
CDV3
CDX1
CDX2
CDX4
CDYL
CDYL2
CEACAM1
CEACAM16
CEACAM18

CEACAM19
CEACAM20
CEACAM21
CEACAM3
CEACAM4
CEACAM5
CEACAM6
CEACAM8
CEBPA
CEBPB
CEBPD
CEBPE
CEBPG
CEBPZ
CEBPZOS
CECR1
CECR2
CECR3
CECR4
CECR5
CECR6
CECR7
CEL
CELA1
CELA2B
CELA3B
CELF1
CELF2
CELF3
CELF4
CELF5
CELF6
CELP
CELSR1
CELSR2
CELSR3
CEMIP
CEMP1
CEND1
CENPB
CENPBD1
CENPBD1P1
CENPC
CENPC1
CENPE
CENPF
CENPH
CENPJ
CENPK
CENPL

CENPM
CENPN
CENPO
CENPP
CENPQ
CENPT
CENPU
CENPV
CEP104
CEP112
CEP120
CEP126
CEP128
CEP131
CEP135
CEP152
CEP164
CEP170
CEP170B
CEP19
CEP192
CEP250
CEP290
CEP295
CEP350
CEP41
CEP44
CEP55
CEP57
CEP63
CEP68
CEP70
CEP72
CEP78
CEP83
CEP85
CEP85L
CEP89
CEP97
CEPT1
CERCAM
CERK
CERKL
CERS2
CERS3
CERS3-AS1
CERS5
CERS6
CES1
CES1P2

CES2
CES3
CES4
CES4A
CES5A
CES5AP1
CES7
CES8
CETN1
CETP
CFAP126
CFAP20
CFAP221
CFAP36
CFAP43
CFAP44
CFAP45
CFAP46
CFAP52
CFAP53
CFAP54
CFAP57
CFAP58
CFAP61
CFAP69
CFAP70
CFAP74
CFAP97
CFAP99
CFB
CFD
CFDP1
CFH
CFHR1
CFHR2
CFHR3
CFHR4
CFHR5
CFI
CFL1
CFL1P1
CFL2
CFLAR
CFP
CFTR
CGB
CGB1
CGB2
CGB5
CGB7

CGB8
CGGBP1
CGN
CGNL1
CGREF1
CGRRF1
CH25H
CHAC1
CHAC2
CHAD
CHADL
CHAF1A
CHAF1B
CHAMP1
CHAT
CHCHD1
CHCHD10
CHCHD2
CHCHD3
CHCHD4
CHCHD5
CHCHD6
CHCHD7
CHCHD8
CHD1
CHD1L
CHD2
CHD3
CHD4
CHD5
CHD6
CHD7
CHD8
CHD9
CHDH
CHEK1
CHEK2
CHERP
CHFR
CHGA
CHGB
CHI3L2
CHIA
CHIC2
CHID1
CHIT1
CHKA
CHKB-AS1
CHKB-CPT1B
CHL1

CHL1-AS1
CHM
CHMP1A
CHMP1B
CHMP2A
CHMP2B
CHMP3
CHMP4A
CHMP4B
CHMP4C
CHMP5
CHMP6
CHMP7
CHN1
CHN2
CHODL
CHORDC1
CHP
CHP1
CHP2
CHPF2
CHPT1
CHRAC1
CHRD
CHRDL1
CHRDL2
CHRM1
CHRM2
CHRM3
CHRM3-AS1
CHRM3-AS2
CHRM4
CHRM5
CHRNA1
CHRNA10
CHRNA2
CHRNA3
CHRNA4
CHRNA5
CHRNA6
CHRNA7
CHRNA9
CHRNB1
CHRNB2
CHRNB3
CHRNB4
CHRND
CHRNE
CHRNG
CHST1

CHST10
CHST11
CHST12
CHST13
CHST14
CHST15
CHST2
CHST3
CHST4
CHST5
CHST6
CHST7
CHST8
CHST9
CHSY1
CHSY3
CHTF18
CHTF8
CHTOP
CHUK
CHURC1
CIAO1
CIAPIN1
CIART
CIB1
CIB2
CIB3
CIB4
CIC
CIDEA
CIDEC
CIDECP
CIITA
CILP
CILP2
CINP
CIR1
CIRBP
CIRH1A
CISD1
CISD2
CISD3
CISH
CISTR
CIT
CITED1
CITED2
CITED4
CIZ1
CKAP2

CKAP2L
CKAP4
CKAP5
CKB
CKLF
CKLF-CMTM1
CKM
CKMT2
CKMT2-AS1
CKS2
CLASP1
CLASP2
CLASRP
CLC
CLCA1
CLCA2
CLCA3P
CLCA4
CLCC1
CLCF1
CLCN1
CLCN2
CLCN3
CLCN4
CLCN5
CLCN6
CLCN7
CLCNKA
CLCNKB
CLDN1
CLDN10
CLDN10-AS1
CLDN11
CLDN12
CLDN14
CLDN15
CLDN16
CLDN17
CLDN18
CLDN19
CLDN2
CLDN20
CLDN23
CLDN24
CLDN25
CLDN3
CLDN34
CLDN5
CLDN6
CLDN7

CLDN9
CLDND1
CLDND2
CLEC10A
CLEC11A
CLEC12A
CLEC14A
CLEC16A
CLEC17A
CLEC18A
CLEC18B
CLEC18C
CLEC1A
CLEC1B
CLEC2A
CLEC2B
CLEC2D
CLEC2L
CLEC3A
CLEC3B
CLEC4A
CLEC4C
CLEC4D
CLEC4E
CLEC4F
CLEC4G
CLEC4GP1
CLEC5A
CLEC6A
CLEC7A
CLEC9A
CLECL1
CLGN
CLIC1
CLIC2
CLIC3
CLIC4
CLIC5
CLIC6
CLINT1
CLIP1
CLIP2
CLIP3
CLIP4
CLK1
CLK2
CLK2P
CLK3
CLK4
CLMN

CLMP
CLN3
CLN5
CLN6
CLN8
CLNK
CLNS1A
CLOCK
CLP1
CLPB
CLPS
CLPSL1
CLPSL2
CLPTM1
CLPTM1L
CLPX
CLRN1
CLRN1-AS1
CLRN1OS
CLRN2
CLRN3
CLSPN
CLSTN1
CLSTN2
CLSTN3
CLTA
CLTB
CLTC
CLTCL1
CLU
CLUAP1
CLUH
CLUL1
CLVS1
CLVS2
CLYBL
CLYBL-AS2
CMAH
CMAHP
CMAS
CMBL
CMC1
CMC2
CMIP
CMKLR1
CMPK1
CMPK2
CMSS1
CMTM1
CMTM2

CMTM3
CMTM4
CMTM5
CMTM6
CMTM7
CMTM8
CMTR1
CMYA5
CN5H6.4
CNBD1
CNBD2
CNBP
CNDP1
CNDP2
CNEP1R1
CNFN
CNGA1
CNGA2
CNGA3
CNGA4
CNGB1
CNGB3
CNIH
CNIH1
CNIH2
CNIH3
CNIH4
CNKSR2
CNKSR3
CNN1
CNN2
CNN3
CNNM1
CNNM2
CNNM3
CNNM4
CNO
CNOT1
CNOT10
CNOT11
CNOT2
CNOT3
CNOT4
CNOT6
CNOT6L
CNOT7
CNOT8
CNP
CNPY1
CNPY2

CNPY3
CNPY4
CNR1
CNR2
CNRIP1
CNST
CNTD1
CNTD2
CNTF
CNTFR
CNTFR-AS1
CNTLN
CNTN1
CNTN2
CNTN3
CNTN4
CNTN5
CNTN6
CNTNAP1
CNTNAP2
CNTNAP3
CNTNAP3P2
CNTNAP4
CNTNAP5
CNTRL
CNTROB
COA1
COA4
COA5
COA6
COA7
COASY
COBL
COBLL1
COBRA1
COCH
COG1
COG2
COG3
COG4
COG5
COG6
COG7
COG8
COIL
COL10A1
COL11A1
COL11A2
COL12A1
COL13A1

COL14A1
COL15A1
COL16A1
COL17A1
COL18A1
COL19A1
COL1A1
COL1A2
COL20A1
COL21A1
COL22A1
COL23A1
COL24A1
COL25A1
COL26A1
COL27A1
COL28A1
COL29A1
COL2A1
COL3A1
COL4A1
COL4A2
COL4A2-AS1
COL4A3
COL4A3BP
COL4A4
COL4A5
COL4A6
COL5A1
COL5A2
COL5A3
COL6A1
COL6A2
COL6A3
COL6A4P1
COL6A4P2
COL6A5
COL7A1
COL8A1
COL8A2
COL9A1
COL9A2
COL9A3
COLCA1
COLEC10
COLEC11
COLEC12
COLGALT1
COLGALT2
COLQ

COMMD1
COMMD10
COMMD2
COMMD3
COMMD4
COMMD5
COMMD6
COMMD7
COMMD8
COMMD9
COMP
COMT
COMTD1
COPA
COPB1
COPB2
COPE
COPG
COPG1
COPG2
COPS2
COPS3
COPS4
COPS5
COPS6
COPS7A
COPS7B
COPS8
COPZ1
COPZ2
COQ10A
COQ10B
COQ2
COQ3
COQ4
COQ5
COQ6
COQ7
COQ9
CORIN
CORO1A
CORO1B
CORO1C
CORO2A
CORO2B
CORO6
CORO7
CORO7-PAM16
CORT
COTL1

COX10
COX10-AS1
COX11
COX15
COX16
COX17
COX19
COX20
COX4NB
COX5A
COX5B
COX6A1
COX6A2
COX6B1
COX6B2
COX6C
COX7A1
COX7A2
COX7B
COX7B2
COX7C
COX8C
CP
CPA1
CPA2
CPA3
CPA4
CPA5
CPA6
CPAMD8
CPB1
CPB2
CPD
CPE
CPEB1
CPEB1-AS1
CPEB2
CPEB2-AS1
CPEB3
CPEB4
CPED1
CPLX1
CPLX2
CPLX3
CPLX4
CPM
CPN1
CPN2
CPNE1
CPNE2

CPNE4
CPNE5
CPNE6
CPNE7
CPNE8
CPNE9
CPO
CPOX
CPPED1
CPQ
CPS1
CPSF1
CPSF2
CPSF3L
CPSF4
CPSF4L
CPSF6
CPSF7
CPT1A
CPT1B
CPT1C
CPT2
CPVL
CPXCR1
CPXM1
CPXM2
CPZ
CR1
CR1L
CR2
CRABP1
CRABP2
CRACR2A
CRADD
CRAMP1L
CRAT
CRAT37
CRAT8
CRB1
CRB2
CRB3
CRBN
CRCP
CRCT1
CREB1
CREB3
CREB3L1
CREB3L2
CREB3L3
CREB3L4

CREB5
CREBBP
CREBL2
CREBRF
CREBZF
CREG1
CREG2
CRELD1
CRELD2
CREM
CRH
CRHBP
CRHR1
CRHR2
CRIM1
CRIP1
CRIP2
CRIP3
CRIPAK
CRIPT
CRISP1
CRISP2
CRISP3
CRISPLD1
CRISPLD2
CRK
CRKL
CRLF1
CRLF3
CRLS1
CRMP1
CRNDE
CRNKL1
CRNN
CROCC
CROCCL1
CROCCL2
CROT
CRP
CRTAC1
CRTAM
CRTAP
CRTC1
CRTC2
CRTC3
CRTC3-AS1
CRX
CRY1
CRY2
CRYAA

CRYAB
CRYBA2
CRYBA4
CRYBB1
CRYBB2
CRYBB2P1
CRYBG3
CRYGA
CRYGC
CRYGD
CRYGN
CRYGS
CRYL1
CRYM
CRYM-AS1
CRYZ
CRYZL1
CS
CSAD
CSAG1
CSDA
CSDAP1
CSDC2
CSDE1
CSE1L
CSF1
CSF1R
CSF2
CSF2RB
CSF3
CSF3R
CSGALNACT1
CSGALNACT2
CSH1
CSHL1
CSK
CSMD1
CSMD2
CSMD2-AS1
CSMD3
CSN1S2AP
CSN3
CSNK1A1
CSNK1A1L
CSNK1A1P
CSNK1A1P1
CSNK1D
CSNK1E
CSNK1G1
CSNK1G2

CSNK1G3
CSNK2A1
CSNK2A2
CSNK2B
CSPG4
CSPG5
CSPP1
CSRNP1
CSRNP2
CSRNP3
CSRP1
CSRP2
CSRP2BP
CSRP3
CST1
CST11
CST13P
CST2
CST3
CST4
CST6
CST7
CST8
CST9
CST9L
CSTA
CSTF1
CSTF2
CSTF3
CSTF3-AS1
CSTL1
CT45A1
CT47B1
CT55
CT62
CT83
CTAGE1
CTAGE5
CTAGE7P
CTB-12O2.1
CTB-178M22.2
CTBP1
CTBP2
CTBS
CTC-436P18.1
CTC1
CTCF
CTCFL
CTD-2194D22.4
CTD-2201E9.1

CTD-2201I18.1
CTD-2270F17.1
CTD-2350J17.1
CTDNEP1
CTDP1
CTDSP1
CTDSP2
CTDSPL
CTDSPL2
CTGF
CTH
CTHRC1
CTIF
CTLA4
CTNNA1
CTNNA2
CTNNA3
CTNNAL1
CTNNB1
CTNNBIP1
CTNNBL1
CTNND1
CTNND2
CTNS
CTPS
CTPS1
CTPS2
CTR9
CTRB1
CTRB2
CTRC
CTSA
CTSB
CTSC
CTSD
CTSE
CTSF
CTSG
CTSH
CTSK
CTSL3P
CTSO
CTSS
CTSZ
CTTN
CTTNBP2
CTTNBP2NL
CTU1
CTU2
CTXN1

CTXN2
CTXN3
CUBN
CUEDC1
CUEDC2
CUGBP1
CUGBP2
CUL1
CUL2
CUL3
CUL4A
CUL4B
CUL5
CUL7
CUL9
CUTA
CUTC
CUX1
CUX2
CUZD1
CWC27
CWF19L1
CWF19L2
CWH43
CX3CL1
CX3CR1
CXADR
CXCL1
CXCL10
CXCL11
CXCL12
CXCL13
CXCL14
CXCL2
CXCL3
CXCL9
CXCR1
CXCR2
CXCR2P1
CXCR4
CXCR5
CXCR6
CXorf1
CXorf15
CXorf21
CXorf22
CXorf23
CXorf26
CXorf27
CXorf30

CXorf36
CXorf38
CXorf39
CXorf40B
CXorf41
CXorf42
CXorf48
CXorf56
CXorf57
CXorf58
CXorf61
CXorf65
CXorf67
CXXC1
CXXC1P1
CXXC5
CYB561
CYB561A3
CYB561D1
CYB561D2
CYB5A
CYB5B
CYB5D2
CYB5R1
CYB5R2
CYB5R3
CYB5R4
CYB5RL
CYBA
CYBASC3
CYBRD1
CYC1
CYFIP1
CYFIP2
CYGB
CYHR1
CYLC2
CYLD
CYMP
CYP11A1
CYP11B1
CYP11B2
CYP17A1
CYP19A1
CYP1A1
CYP1A2
CYP1B1
CYP1B1-AS1
CYP20A1
CYP21A2

CYP24A1
CYP26A1
CYP26B1
CYP26C1
CYP27A1
CYP27B1
CYP27C1
CYP2A13
CYP2A7
CYP2B6
CYP2B7P
CYP2C18
CYP2C19
CYP2C8
CYP2C9
CYP2D6
CYP2D7P1
CYP2E1
CYP2F1
CYP2G1P
CYP2J2
CYP2R1
CYP2S1
CYP2U1
CYP2W1
CYP39A1
CYP3A4
CYP3A43
CYP3A5
CYP3A7
CYP3A7-CYP3A51P
CYP46A1
CYP4A22
CYP4B1
CYP4F11
CYP4F12
CYP4F2
CYP4F22
CYP4F3
CYP4F8
CYP4V2
CYP4X1
CYP4Z1
CYP4Z2P
CYP51A1
CYP51A1-AS1
CYP7A1
CYP7B1
CYP8B1
CYR61

CYS1
CYSLTR1
CYSLTR2
CYSRT1
CYSTM1
CYTH1
CYTH2
CYTH3
CYTH4
CYTIP
CYTL1
CYTSA
CYTSB
CYYR1
D21S2088E
D2HGDH
D4S234E
DAAM1
DAAM2
DAB1
DAB1-AS1
DAB2
DAB2IP
DACH1
DACH2
DACT1
DACT2
DACT3
DAD1
DAG1
DAGLA
DAGLB
DALRD3
DAND5
DAO
DAOA
DAOA-AS1
DAP
DAP3
DAPK1
DAPK2
DAPK3
DAPL1
DAPP1
DARC
DARS
DARS-AS1
DAW1
DAXX
DAZAP1

DAZAP2
DAZL
DBC1
DBF4
DBF4B
DBH
DBI
DBIL5P
DBN1
DBNDD1
DBNDD2
DBNL
DBP
DBR1
DBT
DBX1
DBX2
DCAF10
DCAF11
DCAF12
DCAF12L1
DCAF12L2
DCAF13
DCAF15
DCAF17
DCAF4
DCAF4L1
DCAF4L2
DCAF5
DCAF6
DCAF7
DCAF8
DCAF8L1
DCAF8L2
DCAKD
DCANP1
DCBLD1
DCBLD2
DCC
DCD
DCDC1
DCDC2
DCDC2B
DCDC2C
DCHS1
DCHS2
DCI
DCK
DCLK1
DCLK2

DCLK3
DCLRE1A
DCLRE1B
DCLRE1C
DCN
DCP1A
DCP1B
DCP2
DCPS
DCST1
DCSTAMP
DCT
DCTD
DCTN1
DCTN1-AS1
DCTN2
DCTN3
DCTN4
DCTN5
DCTPP1
DCUN1D1
DCUN1D2
DCUN1D3
DCUN1D4
DCUN1D5
DCXR
DDA1
DDAH1
DDAH2
DDB1
DDB2
DDC
DDC-AS1
DDHD1
DDHD2
DDI1
DDI2
DDIT3
DDIT4L
DDN
DDO
DDOST
DDR1
DDR2
DDRGK1
DDTL
DDX1
DDX10
DDX11
DDX17

DDX18
DDX19A
DDX19B
DDX20
DDX21
DDX23
DDX24
DDX25
DDX26B
DDX27
DDX28
DDX31
DDX39
DDX39A
DDX39B
DDX3X
DDX4
DDX41
DDX42
DDX43
DDX46
DDX47
DDX49
DDX5
DDX50
DDX51
DDX52
DDX54
DDX56
DDX58
DDX59
DDX6
DDX60
DDX60L
DEAF1
1-Dec
DECR1
DECR2
DEDD
DEDD2
DEF6
DEF8
DEFA1
DEFA4
DEFA5
DEFB104A
DEFB104B
DEFB107A
DEFB108B
DEFB113

DEFB115
DEFB118
DEFB119
DEFB121
DEFB122
DEFB123
DEFB124
DEFB126
DEFB127
DEFB128
DEFB129
DEFB131
DEFB132
DEFB133
DEFB134
DEFB135
DEFB136
DEGS1
DEGS2
DEK
DEM1
DENND1A
DENND1B
DENND1C
DENND2A
DENND2C
DENND2D
DENND3
DENND4A
DENND4B
DENND4C
DENND5A
DENND5B
DENND5B-AS1
DENND6B
DENR
DEPDC1
DEPDC1B
DEPDC4
DEPDC5
DEPDC6
DEPDC7
DEPTOR
DERA
DERL1
DERL2
DERL3
DES
DESI1
DESI2

DET1
DEXI
DFFB
DFNA5
DFNB31
DFNB59
DGAT1
DGAT2
DGAT2L6
DGCR14
DGCR2
DGCR5
DGCR6
DGCR6L
DGCR8
DGCR9
DGKA
DGKB
DGKD
DGKE
DGKG
DGKH
DGKI
DGKK
DGKQ
DGKZ
DGUOK
DGUOK-AS1
DHCR24
DHCR7
DHDDS
DHDH
DHDPSL
DHFR
DHFRL1
DHH
DHODH
DHRS1
DHRS11
DHRS12
DHRS13
DHRS2
DHRS3
DHRS4
DHRS4L1
DHRS4L2
DHRS7
DHRS7B
DHRS7C
DHRS9

DHTKD1
DHX15
DHX16
DHX29
DHX30
DHX32
DHX33
DHX34
DHX35
DHX36
DHX37
DHX38
DHX40
DHX40P
DHX57
DHX58
DHX8
DHX9
DIABLO
DIAPH1
DIAPH2
DIAPH2-AS1
DIAPH3
DIAPH3-AS2
DICER1
DIDO1
DIEXF
DIMT1L
DIO1
DIO2
DIO2-AS1
DIO3
DIO3OS
DIP2A
DIP2B
DIP2C
DIRAS1
DIRAS2
DIRAS3
DIRC1
DIRC2
DIRC3
DIS3L
DIS3L2
DISC1
DISC1-IT1
DISC1FP1
DISP1
DISP2
DIXDC1

DKC1
DKFZP434H168
DKFZp434J0226
DKFZP434L187
DKFZp434L192
DKFZp451B082
DKFZP686I15217
DKFZp686K1684
DKFZp686O24166
DKFZp761E198
DKFZp779M0652
DKK1
DKK2
DKK3
DKK4
DKKL1
DLAT
DLC1
DLD
DLEC1
DLEU1
DLEU1-AS1
DLEU2
DLEU7
DLEU7-AS1
DLG1
DLG1-AS1
DLG2
DLG3
DLG4
DLG5
DLGAP1
DLGAP1-AS1
DLGAP1-AS2
DLGAP1-AS4
DLGAP2
DLGAP2-AS1
DLGAP3
DLGAP4
DLGAP4-AS1
DLGAP5
DLK1
DLK2
DLL1
DLL3
DLL4
DLST
DLX1
DLX2
DLX2-AS1

DLX3
DLX4
DLX5
DLX6
DLX6-AS1
DLX6AS
DMAP1
DMBT1
DMBT1P1
DMBX1
DMC1
DMD
DMGDH
DMKN
DMP1
DMPK
DMRT1
DMRT2
DMRT3
DMRTA1
DMRTA2
DMRTB1
DMRTC2
DMTF1
DMTN
DMWD
DMXL1
DMXL2
DNA2
DNAAF1
DNAAF3
DNAAF5
DNAH1
DNAH10
DNAH11
DNAH12
DNAH14
DNAH17
DNAH2
DNAH3
DNAH5
DNAH6
DNAH7
DNAH8
DNAH9
DNAI1
DNAI2
DNAJA1
DNAJA2
DNAJA3

DNAJA4
DNAJB1
DNAJB11
DNAJB12
DNAJB13
DNAJB14
DNAJB2
DNAJB5
DNAJB5-AS1
DNAJB6
DNAJB8
DNAJC1
DNAJC10
DNAJC11
DNAJC12
DNAJC13
DNAJC14
DNAJC15
DNAJC16
DNAJC17
DNAJC18
DNAJC19
DNAJC2
DNAJC21
DNAJC22
DNAJC24
DNAJC27-AS1
DNAJC28
DNAJC3
DNAJC30
DNAJC4
DNAJC5
DNAJC5B
DNAJC5G
DNAJC6
DNAJC7
DNAJC8
DNAJC9
DNAJC9-AS1
DNAL1
DNAL4
DNALI1
DNASE1
DNASE1L1
DNASE1L2
DNASE1L3
DNASE2
DNASE2B
DND1
DNER

DNHD1
DNM1
DNM1L
DNM1P35
DNM2
DNM3
DNM3-IT1
DNMBP
DNMT1
DNMT3A
DNMT3B
DNMT3L
DNPEP
DNPH1
DNTT
DNTTIP1
DNTTIP2
DOC2A
DOC2B
DOCK1
DOCK10
DOCK11
DOCK2
DOCK3
DOCK4
DOCK5
DOCK6
DOCK7
DOCK8
DOCK9
DOCK9-AS1
DOHH
DOK1
DOK2
DOK3
DOK4
DOK5
DOK6
DOK7
DOLK
DOLPP1
DONSON
DOPEY1
DOPEY2
DOT1L
DPAGT1
DPCD
DPCR1
DPEP1
DPEP2

DPEP3
DPF1
DPF2
DPF3
DPH1
DPH2
DPH5
DPH6
DPH6-AS1
DPH7
DPM1
DPM2
DPM3
DPP10
DPP10-AS3
DPP3
DPP4
DPP6
DPP7
DPP8
DPP9
DPP9-AS1
DPPA2
DPPA3
DPPA4
DPPA5
DPRX
DPT
DPY19L1
DPY19L1P1
DPY19L2
DPY19L2P1
DPY19L2P2
DPY19L2P4
DPY19L3
DPY30
DPYD
DPYD-AS1
DPYS
DPYSL2
DPYSL3
DPYSL4
DPYSL5
DQX1
DR1
DRAIC
DRAM1
DRAM2
DRAP1
DRAXIN

DRC1
DRC7
DRD1
DRD2
DRD3
DRD4
DRD5
DRG1
DRG2
DRGX
DROSHA
DSC2
DSC3
DSCAM
DSCAM-AS1
DSCAM-IT1
DSCAML1
DSCC1
DSCR10
DSCR3
DSCR4
DSCR6
DSCR8
DSCR9
DSE
DSEL
DSG1
DSG1-AS1
DSG2
DSG4
DSN1
DSP
DSPP
DST
DSTN
DSTYK
DTD1
DTHD1
DTL
DTNA
DTNB
DTNBP1
DTWD1
DTWD2
DTX1
DTX2P1-UPK3BP1-PMS2P11
DTX3
DTX4
DTYMK
DULLARD

DUOX1
DUOX2
DUOXA1
DUOXA2
DUPD1
DUS1L
DUS2
DUS2L
DUS3L
DUS4L
DUSP1
DUSP10
DUSP11
DUSP12
DUSP13
DUSP14
DUSP15
DUSP16
DUSP18
DUSP19
DUSP2
DUSP21
DUSP22
DUSP23
DUSP26
DUSP27
DUSP28
DUSP3
DUSP4
DUSP5
DUSP5P
DUSP5P1
DUSP6
DUSP7
DUSP8
DUSP9
DUT
DUXA
DVL1
DVL2
DVL3
DVWA
DYDC1
DYDC2
DYM
DYNAP
DYNC1H1
DYNC1I1
DYNC1I2
DYNC1LI1

DYNC1LI2
DYNC2H1
DYNC2LI1
DYNLL1
DYNLL2
DYNLRB1
DYNLRB2
DYNLT1
DYNLT3
DYRK1A
DYRK1B
DYRK2
DYRK3
DYRK4
DYSF
DYSFIP1
DYTN
DYX1C1
DYX1C1-CCPG1
DZANK1
DZIP1
DZIP1L
DZIP3
E2F1
E2F2
E2F3
E2F4
E2F5
E2F6
E2F7
E2F8
E4F1
EAF1
EAPP
EARS2
EBAG9
EBF1
EBF2
EBF3
EBF4
EBI3
EBLN1
EBLN3
EBNA1BP2
EBPL
ECD
ECE1
ECE2
ECEL1
ECH1

ECHDC1
ECHDC2
ECHDC3
ECHS1
ECI1
ECI2
ECM1
ECRP
ECSIT
ECT2
ECT2L
EDA
EDA2R
EDAR
EDARADD
EDC3
EDC4
EDDM3A
EDEM1
EDEM2
EDEM3
EDF1
EDIL3
EDN1
EDN3
EDNRA
EDNRB
EDNRB-AS1
EDRF1
EEA1
EED
EEF1A1
EEF1A2
EEF1B2
EEF1D
EEF1DP3
EEF1E1-BLOC1S5
EEF1G
EEF2
EEF2K
EEFSEC
EEPD1
EFCAB10
EFCAB11
EFCAB12
EFCAB13
EFCAB14
EFCAB14-AS1
EFCAB2
EFCAB3

EFCAB4A
EFCAB4B
EFCAB5
EFCAB6
EFCAB6-AS1
EFCAB8
EFCAB9
EFCC1
EFEMP1
EFEMP2
EFHA1
EFHA2
EFHB
EFHC1
EFHC2
EFHD1
EFHD2
EFNA1
EFNA2
EFNA3
EFNA4
EFNA5
EFNB1
EFNB2
EFNB3
EFR3A
EFR3B
EFS
EFTUD1
EFTUD1P1
EFTUD2
EGF
EGFEM1P
EGFL6
EGFL7
EGFL8
EGFLAM
EGFLAM-AS2
EGFLAM-AS4
EGFR
EGFR-AS1
EGLN1
EGLN2
EGLN3
EGR1
EGR2
EGR3
EGR4
EHBP1
EHBP1L1

EHD1
EHD2
EHD3
EHD4
EHD4-AS1
EHF
EHHADH
EHHADH-AS1
EHMT1
EHMT2
EI24
EID1
EID2
EID2B
EIF1
EIF1AD
EIF1AX
EIF1AX-AS1
EIF1AY
EIF1B
EIF1B-AS1
EIF2A
EIF2AK1
EIF2AK2
EIF2AK3
EIF2AK4
EIF2B1
EIF2B2
EIF2B3
EIF2B4
EIF2B5
EIF2B5-AS1
EIF2C1
EIF2C2
EIF2C3
EIF2C4
EIF2D
EIF2S1
EIF2S2
EIF2S3
EIF3A
EIF3B
EIF3D
EIF3E
EIF3F
EIF3G
EIF3H
EIF3I
EIF3J
EIF3J-AS1

EIF3L
EIF3M
EIF4A1
EIF4A2
EIF4A3
EIF4B
EIF4E
EIF4E1B
EIF4E2
EIF4E3
EIF4EBP1
EIF4EBP2
EIF4EBP3
EIF4ENIF1
EIF4G1
EIF4G2
EIF4G3
EIF4H
EIF5
EIF5A
EIF5A2
EIF5B
EIF6
ELAC1
ELAC2
ELANE
ELAVL1
ELAVL2
ELAVL3
ELAVL4
ELF1
ELF2
ELF3
ELF4
ELF5
ELFN1
ELFN1-AS1
ELFN2
ELK1
ELK3
ELK4
ELL
ELL2
ELL3
ELMO1
ELMO1-AS1
ELMO2
ELMO3
ELMOD1
ELMOD2

ELMOD3
ELMSAN1
ELN
ELOF1
ELOVL1
ELOVL2
ELOVL2-AS1
ELOVL3
ELOVL4
ELOVL5
ELOVL6
ELOVL7
ELP2
ELP3
ELP4
ELP6
ELSPBP1
ELTD1
EMB
EMBP1
EMC1
EMC10
EMC2
EMC3
EMC4
EMC7
EMC9
EMCN
EME1
EME2
EMG1
EMID1
EMID2
EMILIN1
EMILIN2
EMILIN3
EML1
EML2
EML2-AS1
EML3
EML4
EML5
EML6
EMP1
EMP2
EMP3
EMR1
EMR2
EMR3
EMX1

EMX2
EMX2OS
EN1
EN2
ENAH
ENAM
ENC1
ENDOD1
ENDOG
ENDOU
ENDOV
ENG
ENGASE
ENKD1
ENKUR
ENO1
ENO2
ENO3
ENO4
ENOPH1
ENOSF1
ENOX1
ENOX2
ENPEP
ENPP1
ENPP2
ENPP3
ENPP4
ENPP5
ENPP6
ENPP7
ENSA
ENTHD1
ENTHD2
ENTPD1
ENTPD1-AS1
ENTPD2
ENTPD3
ENTPD4
ENTPD5
ENTPD6
ENTPD7
ENTPD8
ENY2
EOGT
EOMES
EP300
EP300-AS1
EP400
EP400NL

EPAS1
EPB41
EPB41L1
EPB41L2
EPB41L3
EPB41L4A
EPB41L4B
EPB41L5
EPB42
EPB49
EPC1
EPC2
EPCAM
EPDR1
EPG5
EPGN
EPHA1
EPHA10
EPHA2
EPHA3
EPHA4
EPHA5
EPHA6
EPHA7
EPHA8
EPHB1
EPHB2
EPHB3
EPHB4
EPHB6
EPHX1
EPHX2
EPHX3
EPHX4
EPM2A
EPM2AIP1
EPN1
EPN2
EPN2-AS1
EPN3
EPO
EPOR
EPPK1
EPR1
EPRS
EPS15
EPS15L1
EPS8
EPS8L1
EPS8L2

EPSTI1
EPT1
EPX
EPYC
EQTN
ERAL1
ERAP1
ERAP2
ERAS
ERBB2
ERBB2IP
ERBB3
ERBB4
ERC1
ERC2
ERCC1
ERCC2
ERCC3
ERCC4
ERCC5
ERCC6
ERCC6L
ERCC6L2
ERCC8
EREG
ERF
ERG
ERGIC1
ERGIC2
ERGIC3
ERH
ERI1
ERI2
ERI3
ERI3-IT1
ERICH1
ERICH1-AS1
ERICH2
ERICH3
ERICH3-AS1
ERICH4
ERICH5
ERICH6
ERICH6-AS1
ERICH6B
ERLIN1
ERMAP
ERMARD
ERMN
ERMP1

ERN1
ERN2
ERO1A
ERO1LB
ERP27
ERP29
ERP44
ERRFI1
ERV3-1
ERVFRD-1
ERVH48-1
ERVK13-1
ERVMER34-1
ERVV-1
ESAM
ESCO1
ESCO2
ESD
ESF1
ESM1
ESPL1
ESPN
ESPNL
ESPNP
ESR1
ESR2
ESRP1
ESRP2
ESRRA
ESRRB
ESRRG
ESX1
ESYT1
ESYT2
ESYT3
ETF1
ETFA
ETFB
ETFDH
ETHE1
ETNK1
ETNK2
ETNPPL
ETS1
ETS2
ETV1
ETV2
ETV3
ETV3L
ETV4

ETV5
ETV6
ETV7
EVA1A
EVA1C
EVADR
EVC
EVC2
EVI2A
EVI2B
EVI5
EVI5L
EVL
EVPL
EVPLL
EVX1
EVX1-AS
EVX2
EWSAT1
EWSR1
EXD1
EXD2
EXD3
EXO1
EXOC1
EXOC2
EXOC3
EXOC3L
EXOC3L1
EXOC3L2
EXOC3L4
EXOC4
EXOC5
EXOC6
EXOC6B
EXOC7
EXOC8
EXOG
EXOSC1
EXOSC10
EXOSC2
EXOSC3
EXOSC4
EXOSC5
EXOSC6
EXOSC7
EXOSC9
EXPH5
EXT1
EXT2

EXTL1
EXTL2
EXTL3
EXTL3-AS1
EYA1
EYA2
EYA3
EYA4
EYS
EZH1
EZH2
EZR
F10
F11
F11-AS1
F11R
F12
F13A1
F13B
F2
F2R
F2RL1
F2RL2
F2RL3
F3
F5
F7
F8
F9
FA2H
FAAH
FAAH2
FAAHP1
FAAP100
FAAP20
FAAP24
FABP1
FABP2
FABP4
FABP5
FABP5L3
FABP6
FADD
FADS1
FADS2
FADS3
FADS6
FAF1
FAF2
FAH

FAHD1
FAHD2A
FAHD2B
FAIM
FAIM2
FAIM3
FALEC
FAM100A
FAM100B
FAM101A
FAM101B
FAM102A
FAM102B
FAM104A
FAM104B
FAM105A
FAM105B
FAM107A
FAM107B
FAM108A1
FAM108C1
FAM109A
FAM109B
FAM10A4
FAM110A
FAM110B
FAM110C
FAM110D
FAM111A
FAM111B
FAM113A
FAM113B
FAM114A1
FAM115A
FAM115C
FAM117A
FAM118A
FAM118B
FAM119B
FAM120A
FAM120AOS
FAM120B
FAM120C
FAM122A
FAM122B
FAM122C
FAM123A
FAM123B
FAM123C
FAM124A

FAM124B
FAM125A
FAM125B
FAM126A
FAM126B
FAM127B
FAM127C
FAM128A
FAM129A
FAM129B
FAM129C
FAM12A
FAM12B
FAM131A
FAM131B
FAM131C
FAM132A
FAM132B
FAM133A
FAM133B
FAM134A
FAM134B
FAM134C
FAM135A
FAM135B
FAM136A
FAM138D
FAM13A
FAM13A-AS1
FAM13B
FAM13C
FAM149A
FAM149B1
FAM150A
FAM150B
FAM151B
FAM153A
FAM153B
FAM153C
FAM154A
FAM155A
FAM155A-IT1
FAM156A
FAM157A
FAM157B
FAM157C
FAM158A
FAM159A
FAM159B
FAM160A1

FAM160A2
FAM160B1
FAM160B2
FAM161A
FAM161B
FAM162A
FAM162B
FAM163A
FAM163B
FAM164A
FAM164C
FAM165B
FAM166A
FAM166B
FAM167A
FAM167A-AS1
FAM167B
FAM168A
FAM168B
FAM169A
FAM169B
FAM170B
FAM171A1
FAM171A2
FAM171B
FAM172A
FAM172BP
FAM173A
FAM173B
FAM174A
FAM174B
FAM175A
FAM175B
FAM176A
FAM176B
FAM177A1
FAM177B
FAM178A
FAM178B
FAM179A
FAM179B
FAM180A
FAM180B
FAM181A
FAM181A-AS1
FAM181B
FAM183A
FAM183B
FAM184A
FAM184B

FAM185A
FAM186B
FAM187B
FAM188A
FAM188B
FAM189A1
FAM189A2
FAM189B
FAM18A
FAM18B
FAM18B2
FAM190A
FAM190B
FAM192A
FAM193A
FAM193B
FAM194A
FAM194B
FAM195A
FAM195B
FAM196A
FAM196B
FAM197Y2
FAM198A
FAM198B
FAM199X
FAM19A1
FAM19A2
FAM19A3
FAM19A4
FAM19A5
FAM200A
FAM200B
FAM205BP
FAM207A
FAM208A
FAM209B
FAM20A
FAM20B
FAM20C
FAM210A
FAM210B
FAM212B
FAM213A
FAM214A
FAM216A
FAM216B
FAM217A
FAM217B
FAM219A

FAM219B
FAM21B
FAM21C
FAM21EP
FAM220A
FAM221A
FAM221B
FAM222A
FAM222A-AS1
FAM222B
FAM227A
FAM227B
FAM228A
FAM228B
FAM229B
FAM24A
FAM24B
FAM24B-CUZD1
FAM25A
FAM25G
FAM26D
FAM26E
FAM27L
FAM32A
FAM35B
FAM35BP
FAM36A
FAM38A
FAM38B
FAM3A
FAM3B
FAM3C
FAM3D
FAM40A
FAM40B
FAM41C
FAM43A
FAM43B
FAM45A
FAM45B
FAM46A
FAM46B
FAM46C
FAM47A
FAM47B
FAM47C
FAM47E
FAM47E-STBD1
FAM48B1
FAM48B2

FAM49A
FAM49B
FAM50A
FAM50B
FAM53A
FAM53B
FAM53C
FAM54A
FAM54B
FAM55B
FAM55C
FAM57A
FAM57B
FAM58A
FAM58B
FAM59A
FAM59B
FAM5B
FAM60A
FAM63A
FAM63B
FAM64A
FAM65A
FAM65B
FAM65C
FAM66A
FAM66C
FAM66D
FAM66E
FAM69A
FAM69B
FAM69C
FAM70A
FAM71B
FAM71C
FAM71D
FAM71E1
FAM71E2
FAM71F1
FAM71F2
FAM72A
FAM72B
FAM72D
FAM73A
FAM73B
FAM75A3
FAM75C1
FAM76A
FAM76B
FAM78A

FAM78B
FAM7A3
FAM81A
FAM81B
FAM82A1
FAM82A2
FAM82B
FAM83A
FAM83A-AS1
FAM83B
FAM83C
FAM83D
FAM83E
FAM83F
FAM83G
FAM83H
FAM83H-AS1
FAM84A
FAM84B
FAM86B2
FAM86B3P
FAM86C
FAM86EP
FAM86FP
FAM87A
FAM87B
FAM89A
FAM89B
FAM8A1
FAM90A14
FAM90A25P
FAM90A7
FAM90A8
FAM92A1
FAM92A1P2
FAM92A3
FAM92B
FAM96B
FAM98A
FAM98B
FAM98C
FAM9A
FAM9B
FAM9C
FAN1
FANCA
FANCB
FANCC
FANCD2
FANCD2OS

FANCE
FANCF
FANCG
FANCI
FANCL
FANCM
FANK1
FANK1-AS1
FAP
FAR1
FAR2
FARP1
FARP2
FARS2
FARSA
FARSB
FAS
FASLG
FASN
FASTK
FASTKD1
FASTKD2
FASTKD5
FAT1
FAT2
FAT3
FAT4
FATE1
FAXC
FAXDC2
FBF1
FBL
FBLIM1
FBLL1
FBLN1
FBLN2
FBLN5
FBLN7
FBN1
FBN2
FBN3
FBP1
FBP2
FBRS
FBRSL1
FBXL12
FBXL13
FBXL14
FBXL15
FBXL16

FBXL17
FBXL18
FBXL19
FBXL2
FBXL20
FBXL21
FBXL22
FBXL3
FBXL4
FBXL5
FBXL6
FBXL7
FBXL8
FBXO10
FBXO11
FBXO15
FBXO16
FBXO17
FBXO18
FBXO2
FBXO21
FBXO22
FBXO22OS
FBXO24
FBXO25
FBXO27
FBXO28
FBXO3
FBXO3-AS1
FBXO30
FBXO31
FBXO32
FBXO33
FBXO34
FBXO36
FBXO38
FBXO39
FBXO4
FBXO40
FBXO41
FBXO42
FBXO43
FBXO44
FBXO46
FBXO5
FBXO6
FBXO7
FBXO8
FBXO9
FBXW10

FBXW11
FBXW12
FBXW2
FBXW4
FBXW5
FBXW7
FBXW8
FBXW9
FCAMR
FCAR
FCER1A
FCER1G
FCER2
FCF1
FCGBP
FCGR1B
FCGR2A
FCGR2B
FCGR2C
FCGR3A
FCGRT
FCHO1
FCHO2
FCHSD1
FCHSD2
FCMR
FCN1
FCN2
FCN3
FCRL1
FCRL3
FCRL4
FCRL5
FCRL6
FCRLA
FCRLB
FDFT1
FDPS
FDX1
FDX1L
FDXACB1
FDXR
FECH
FEM1A
FEM1B
FEM1C
FEN1
FER
FER1L4
FER1L5

FER1L6
FER1L6-AS2
FERD3L
FERMT1
FERMT2
FERMT3
FES
FEV
FEZ1
FEZ2
FEZF1
FEZF1-AS1
FEZF2
FFAR1
FFAR2
FFAR3
FFAR4
FGA
FGB
FGD1
FGD2
FGD3
FGD4
FGD5
FGD6
FGF1
FGF10
FGF11
FGF12
FGF12-AS1
FGF13
FGF13-AS1
FGF14
FGF14-IT1
FGF17
FGF18
FGF19
FGF2
FGF20
FGF21
FGF22
FGF3
FGF4
FGF5
FGF6
FGF7
FGF8
FGF9
FGFBP1
FGFBP2

FGFBP3
FGFR1
FGFR1OP
FGFR2
FGFR3
FGFR4
FGFRL1
FGG
FGGY
FGL1
FGR
FH
FHAD1
FHDC1
FHIT
FHL1
FHL2
FHL3
FHL5
FHOD1
FHOD3
FIBCD1
FIBIN
FICD
FIG4
FIGF
FIGLA
FIGN
FIGNL1
FIGNL2
FILIP1
FILIP1L
FIP1L1
FIRRE
FIS1
FITM1
FITM2
FIZ1
FKBP10
FKBP11
FKBP14
FKBP15
FKBP1A
FKBP1A-SDCBP2
FKBP1B
FKBP2
FKBP4
FKBP5
FKBP6
FKBP7

FKBP8
FKBP9
FKBP9L
FKBPL
FKRP
FKSG83
FKTN
FLAD1
FLCN
FLG
FLG-AS1
FLG2
FLI1
FLII
FLJ10357
FLJ11235
FLJ12825
FLJ13197
FLJ16171
FLJ16779
FLJ20021
FLJ20184
FLJ21408
FLJ22447
FLJ22536
FLJ23834
FLJ23867
FLJ25006
FLJ25328
FLJ26245
FLJ26850
FLJ30058
FLJ31306
FLJ31356
FLJ32063
FLJ32065
FLJ32810
FLJ33360
FLJ33534
FLJ33581
FLJ33630
FLJ34503
FLJ35024
FLJ35220
FLJ35390
FLJ35776
FLJ36000
FLJ36031
FLJ36777
FLJ37307

FLJ37453
FLJ37505
FLJ37543
FLJ39582
FLJ39609
FLJ39653
FLJ39739
FLJ40125
FLJ40288
FLJ40330
FLJ40434
FLJ40504
FLJ40852
FLJ41278
FLJ41350
FLJ41562
FLJ41603
FLJ42102
FLJ42289
FLJ42627
FLJ42709
FLJ42875
FLJ42969
FLJ43390
FLJ43663
FLJ43860
FLJ43879
FLJ43950
FLJ44606
FLJ44635
FLJ44817
FLJ45079
FLJ45244
FLJ45513
FLJ45983
FLJ46321
FLJ46906
FLJ90757
FLNA
FLNB
FLNC
FLOT1
FLOT2
FLRT1
FLRT2
FLT1
FLT3
FLT3LG
FLT4
FLVCR1

FLVCR1-AS1
FLVCR2
FLYWCH1
FLYWCH2
FMN1
FMN2
FMNL1
FMNL2
FMNL3
FMO1
FMO2
FMO3
FMO4
FMO6P
FMO9P
FMOD
FMR1
FN1
FN3K
FN3KRP
FNBP1
FNBP1L
FNBP4
FNDC1
FNDC3A
FNDC3B
FNDC4
FNDC5
FNDC7
FNDC8
FNDC9
FNIP1
FNIP2
FNTA
FNTB
FOCAD
FOCAD-AS1
FOLH1
FOLR1
FOLR2
FOLR3
FOLR4
FOPNL
FOS
FOSB
FOSL1
FOSL2
FOXA1
FOXA2
FOXA3

FOXB1
FOXB2
FOXC1
FOXC2
FOXD1
FOXD2
FOXD3
FOXD3-AS1
FOXD4
FOXD4L1
FOXD4L3
FOXE1
FOXE3
FOXF1
FOXF2
FOXG1
FOXG1-AS1
FOXH1
FOXI1
FOXI3
FOXJ1
FOXJ2
FOXJ3
FOXK1
FOXK2
FOXL1
FOXL2
FOXM1
FOXN1
FOXN2
FOXN3
FOXN4
FOXO1
FOXO3
FOXO4
FOXP1
FOXP1-AS1
FOXP2
FOXP3
FOXP4
FOXR1
FOXR2
FOXRED1
FOXRED2
FOXS1
FPGS
FPGT-TNNI3K
FPR1
FPR2
FPR3

FRA10AC1
FRAS1
FRAT1
FRAT2
FREM1
FREM2
FREM3
FREQ
FRG1
FRG1B
FRK
FRMD1
FRMD3
FRMD4A
FRMD4B
FRMD5
FRMD6
FRMD6-AS2
FRMD7
FRMD8
FRMPD1
FRMPD2
FRMPD3
FRMPD3-AS1
FRMPD4
FRRS1
FRRS1L
FRS2
FRS3
FRY
FRY-AS1
FRYL
FRZB
FSBP
FSCB
FSCN1
FSCN2
FSCN3
FSD1
FSD1L
FSD2
FSHB
FSHR
FSIP1
FSIP2
FSTL1
FSTL3
FSTL4
FSTL5
FTCD

FTCDNL1
FTH1
FTH1P18
FTHL17
FTL
FTO
FTO-IT1
FTSJ1
FTSJ2
FTSJ3
FTSJD1
FTSJD2
FTX
FUBP1
FUBP3
FUCA1
FUK
FUNDC1
FUNDC2
FUOM
FURIN
FUS
FUT1
FUT10
FUT11
FUT2
FUT3
FUT4
FUT5
FUT6
FUT8
FUT9
FUZ
FXC1
FXN
FXR1
FXR2
FXYD1
FXYD2
FXYD3
FXYD4
FXYD5
FXYD6
FXYD6-FXYD2
FXYD7
FYB
FYCO1
FYN
FYTTD1
FZD1

FZD10
FZD2
FZD3
FZD4
FZD5
FZD6
FZD7
FZD8
FZD9
FZR1
G0S2
G2E3
G3BP1
G3BP2
G6PC
G6PC2
G6PC3
GAA
GAB1
GAB2
GAB3
GABARAP
GABARAPL1
GABARAPL2
GABBR1
GABBR2
GABPB1
GABPB2
GABRA1
GABRA2
GABRA3
GABRA4
GABRA5
GABRA6
GABRB1
GABRB2
GABRB3
GABRD
GABRE
GABRG2
GABRG3
GABRP
GABRQ
GABRR1
GABRR2
GABRR3
GACAT1
GACAT2
GACAT3
GAD1

GAD2
GADD45A
GADD45B
GADD45G
GADD45GIP1
GADL1
GAGE1
GAGE10
GAGE2A
GAK
GAL
GAL3ST1
GAL3ST2
GAL3ST3
GALC
GALE
GALK1
GALK2
GALM
GALNS
GALNT1
GALNT10
GALNT11
GALNT12
GALNT13
GALNT14
GALNT15
GALNT16
GALNT18
GALNT2
GALNT5
GALNT6
GALNT7
GALNT8
GALNT9
GALNTL1
GALNTL2
GALNTL4
GALNTL5
GALNTL6
GALP
GALR1
GALR2
GALR3
GALT
GAMT
GAN
GANAB
GANC
GAP43

GAPDH
GAPDHS
GAPLINC
GAPT
GAPVD1
GAR1
GAREM
GAREML
GARNL3
GARS
GART
GAS1
GAS2
GAS2L1
GAS2L2
GAS2L3
GAS5
GAS5-AS1
GAS7
GAS8
GAS8-AS1
GAST
GATA1
GATA2
GATA3
GATA3-AS1
GATA4
GATA5
GATA6
GATAD1
GATAD2A
GATAD2B
GATB
GATC
GATS
GATSL1
GATSL3
GBA
GBA2
GBA3
GBAP1
GBAS
GBAT2
GBE1
GBF1
GBGT1
GBP1
GBP2
GBP3
GBP4

GBP5
GBP6
GBP7
GBX1
GBX2
GC
GCA
GCAT
GCC1
GCC2
GCDH
GCET2
GCFC2
GCH1
GCHFR
GCK
GCKR
GCLC
GCLM
GCM1
GCM2
GCN1
GCN1L1
GCNT1
GCNT2
GCNT3
GCNT7
GCOM1
GCSAM
GCSAML
GCSH
GDA
GDAP1
GDAP1L1
GDAP2
GDE1
GDEP
GDF1
GDF10
GDF11
GDF15
GDF2
GDF5
GDF6
GDF7
GDF9
GDI1
GDI2
GDNF
GDPD1

GDPD2
GDPD3
GDPD4
GDPD5
GEFT
GEM
GEMIN4
GEMIN5
GEMIN6
GEMIN7
GEMIN8
GEMIN8P4
GEN1
GFAP
GFER
GFI1
GFI1B
GFM1
GFM2
GFOD1
GFOD2
GFPT1
GFPT2
GFRA1
GFRA2
GFRA3
GFRA4
GFRAL
GFY
GGA1
GGA2
GGA3
GGACT
GGCT
GGCX
GGH
GGN
GGNBP2
GGT1
GGT5
GGT6
GGT7
GGTA1
GGTA1P
GGTLC2
GH1
GH2
GHDC
GHITM
GHR

GHRHR
GHRLOS
GHSR
GID4
GIF
GIGYF1
GIGYF2
GIMAP1
GIMAP2
GIMAP4
GIMAP6
GIMAP7
GIMAP8
GIMD1
GINM1
GINS1
GINS2
GINS3
GINS4
GIP
GIPC1
GIPC2
GIPC3
GIPR
GIT1
GIT2
GIYD1
GJA1
GJA10
GJA3
GJA4
GJA5
GJA8
GJB1
GJB2
GJB3
GJB6
GJB7
GJC1
GJC2
GJC3
GJD2
GJD3
GJD4
GK
GK2
GK5
GKN1
GLA
GLB1

GLB1L
GLB1L2
GLB1L3
GLCCI1
GLCE
GLDC
GLDN
GLE1
GLG1
GLI1
GLI2
GLI3
GLI4
GLIPR1
GLIPR1L2
GLIPR2
GLIS1
GLIS2
GLIS2-AS1
GLIS3
GLMN
GLMP
GLO1
GLOD4
GLOD5
GLP1R
GLP2R
GLRA1
GLRA2
GLRA3
GLRA4
GLRB
GLRX
GLRX2
GLRX3
GLRX5
GLS
GLS2
GLT1D1
GLT25D1
GLT25D2
GLT8D1
GLTPD1
GLTSCR1
GLTSCR2
GLUD1
GLUD1P7
GLUD2
GLUL
GLYAT

GLYATL1
GLYATL2
GLYCAM1
GLYCTK
GLYR1
GM2A
GMCL1
GMCL1P1
GMDS
GMDS-AS1
GMEB1
GMEB2
GMFB
GMFG
GMIP
GML
GMNC
GMNN
GMPPA
GMPPB
GMPR
GMPR2
GMPS
GNA11
GNA12
GNA13
GNA14
GNA15
GNAI1
GNAI2
GNAI3
GNAL
GNAO1
GNAQ
GNAS
GNASAS
GNAT1
GNAT2
GNAT3
GNAZ
GNB1
GNB1L
GNB2
GNB2L1
GNB3
GNB4
GNB5
GNE
GNG10
GNG12

GNG12-AS1
GNG13
GNG2
GNG3
GNG4
GNG5
GNG7
GNG8
GNGT2
GNL1
GNL2
GNL3
GNL3L
GNLY
GNMT
GNPAT
GNPDA1
GNPNAT1
GNPTAB
GNPTG
GNRH1
GNRH2
GNRHR
GNRHR2
GNS
GOLGA1
GOLGA2P6
GOLGA2P9
GOLGA3
GOLGA4
GOLGA5
GOLGA6A
GOLGA6B
GOLGA6D
GOLGA6L1
GOLGA6L2
GOLGA6L7P
GOLGA7
GOLGA8A
GOLGA8B
GOLGA8C
GOLGA8E
GOLGA8F
GOLGA8M
GOLGA8R
GOLGB1
GOLIM4
GOLM1
GOLPH3
GOLPH3L

GOLSYN
GOLT1A
GOLT1B
GON4L
GOPC
GORAB
GORASP1
GORASP2
GOSR1
GOSR2
GOT1
GOT1L1
GOT2
GP1BA
GP2
GP5
GP6
GP9
GPA33
GPAA1
GPAM
GPAT2
GPATCH1
GPATCH11
GPATCH2
GPATCH2L
GPATCH3
GPATCH4
GPATCH8
GPBAR1
GPBP1
GPBP1L1
GPC1
GPC2
GPC3
GPC4
GPC5
GPC5-AS1
GPC6
GPC6-AS1
GPCPD1
GPD1
GPD1L
GPD2
GPER
GPER1
GPHA2
GPHB5
GPHN
GPI

GPIHBP1
GPKOW
GPLD1
GPM6A
GPM6B
GPN1
GPN2
GPN3
GPNMB
GPR1
GPR1-AS
GPR107
GPR108
GPR109A
GPR110
GPR111
GPR112
GPR113
GPR114
GPR115
GPR116
GPR119
GPR12
GPR120
GPR123
GPR124
GPR125
GPR126
GPR128
GPR132
GPR133
GPR135
GPR137
GPR137B
GPR137C
GPR139
GPR141
GPR142
GPR143
GPR144
GPR146
GPR148
GPR149
GPR15
GPR150
GPR152
GPR153
GPR155
GPR156
GPR157

GPR158
GPR160
GPR161
GPR162
GPR17
GPR172B
GPR173
GPR176
GPR177
GPR179
GPR18
GPR180
GPR182
GPR183
GPR19
GPR20
GPR21
GPR25
GPR26
GPR27
GPR3
GPR31
GPR33
GPR34
GPR35
GPR37
GPR37L1
GPR39
GPR4
GPR44
GPR45
GPR50
GPR52
GPR55
GPR56
GPR6
GPR61
GPR62
GPR63
GPR64
GPR65
GPR68
GPR75
GPR75-ASB3
GPR77
GPR78
GPR81
GPR83
GPR84
GPR85

GPR87
GPR88
GPR89A
GPR89B
GPR98
GPRASP1
GPRASP2
GPRC5A
GPRC5B
GPRC5C
GPRC5D
GPRC6A
GPRIN1
GPRIN2
GPRIN3
GPS1
GPS2
GPSM1
GPSM2
GPSM3
GPT
GPT2
GPX1
GPX2
GPX3
GPX4
GPX5
GPX6
GPX7
GPX8
GRAMD1A
GRAMD1B
GRAMD1C
GRAMD2
GRAMD3
GRAMD4
GRAP2
GRASP
GRB10
GRB14
GRB2
GRB7
GREB1
GREB1L
GREM1
GREM2
GRHL1
GRHL2
GRHL3
GRIA1

GRIA2
GRIA3
GRIA4
GRID1
GRID1-AS1
GRID2
GRID2IP
GRIFIN
GRIK1
GRIK1-AS1
GRIK1-AS2
GRIK2
GRIK3
GRIK4
GRIK5
GRIN1
GRIN2A
GRIN2B
GRIN2C
GRIN2D
GRIN3A
GRIN3B
GRINA
GRIP1
GRIP2
GRIPAP1
GRK1
GRK4
GRK5
GRK6
GRK7
GRLF1
GRM1
GRM2
GRM3
GRM4
GRM5
GRM5-AS1
GRM6
GRM7
GRM7-AS1
GRM7-AS2
GRM7-AS3
GRM8
GRN
GRP
GRPEL1
GRPEL2
GRPR
GRRP1

GRSF1
GRTP1
GRTP1-AS1
GRWD1
GRXCR1
GS1-124K5.11
GS1-259H13.2
GS1-279B7.1
GSAP
GSC
GSC2
GSDMA
GSDMB
GSDMD
GSG1
GSG1L
GSG1L2
GSK3A
GSK3B
GSN
GSPT1
GSPT2
GSR
GSS
GSTA1
GSTA3
GSTA4
GSTA5
GSTCD
GSTM2
GSTM4
GSTM5
GSTO1
GSTO2
GSTP1
GSTT1
GSTTP2
GSTZ1
GSX1
GSX2
GTDC1
GTF2A1
GTF2A1L
GTF2A2
GTF2B
GTF2E1
GTF2E2
GTF2F1
GTF2F2
GTF2H1

GTF2H2C
GTF2H2C_2
GTF2H3
GTF2H4
GTF2H5
GTF2I
GTF2IRD1
GTF2IRD1P1
GTF2IRD2P
GTF3A
GTF3C1
GTF3C2
GTF3C2-AS1
GTF3C3
GTF3C4
GTF3C5
GTF3C6
GTPBP1
GTPBP10
GTPBP2
GTPBP3
GTPBP4
GTPBP5
GTPBP8
GTSE1-AS1
GTSF1
GTSF1L
GUCA1A
GUCA1C
GUCA2A
GUCA2B
GUCY1A2
GUCY1A3
GUCY1B2
GUCY1B3
GUCY2C
GUCY2D
GUCY2E
GUCY2EP
GUCY2F
GUCY2GP
GUF1
GUK1
GULP1
GUSB
GUSBL1
GUSBL2
GUSBP1
GUSBP11
GXYLT1

GXYLT2
GYG1
GYG2
GYG2P1
GYLTL1B
GYPA
GYPB
GYPC
GYPE
GYS1
GYS2
GZF1
GZMA
GZMB
GZMH
GZMK
GZMM
H19
H1F0
H1FOO
H1FX
H1FX-AS1
H2AFJ
H2AFV
H2AFX
H2AFY
H2AFY2
H2AFZ
H2BFM
H2BFWT
H3F3A
H3F3B
H6PD
HAAO
HABP2
HABP4
HACD1
HACD3
HACD4
HACE1
HACL1
HADH
HADHA
HADHB
HAGH
HAGHL
HAGLR
HAL
HAMP
HAND1

HAND2
HAND2-AS1
HAO1
HAP1
HAPLN1
HAPLN2
HAPLN3
HAPLN4
HAR1A
HARBI1
HARS
HARS2
HAS1
HAS2
HAS2AS
HAS3
HAT1
HAUS1
HAUS2
HAUS3
HAUS4
HAUS6
HAUS7
HAUS8
HAVCR1
HAVCR2
HAX1
HBA1
HBB
HBBP1
HBD
HBE1
HBEGF
HBG1
HBG2
HBM
HBP1
HBQ1
HBS1L
HBXIP
HCAR2
HCCA2
HCCS
HCFC1
HCFC1R1
HCG11
HCG17
HCG18
HCG2040054
HCG22

HCG23
HCG26
HCG27
HCG4
HCG4P6
HCG9
HCK
HCLS1
HCN1
HCN2
HCN3
HCN4
HCP5
HCRT
HCRTR1
HCRTR2
HCST
HDAC1
HDAC10
HDAC11
HDAC11-AS1
HDAC2
HDAC3
HDAC4
HDAC5
HDAC6
HDAC7
HDAC8
HDAC9
HDC
HDDC2
HDDC3
HDGF
HDGF2
HDGFL1
HDGFRP3
HDHD1
HDHD1A
HDHD2
HDHD3
HDLBP
HDX
HEATR1
HEATR2
HEATR3
HEATR4
HEATR5A
HEATR5B
HEATR6
HEATR7A

HEBP1
HEBP2
HECA
HECTD1
HECTD2
HECTD2-AS1
HECTD3
HECTD4
HECW1
HECW2
HEG1
HELB
HELLS
HELQ
HELT
HELZ
HELZ2
HEMGN
HEMK1
HENMT1
HEPACAM
HEPACAM2
HEPH
HEPHL1
HERC1
HERC2
HERC2P2
HERC3
HERC4
HERC5
HERC6
HERPUD1
HERPUD2
HES1
HES2
HES3
HES4
HES5
HES6
HES7
HESX1
HEXA
HEXA-AS1
HEXB
HEXDC
HEXIM1
HEXIM2
HEY1
HEY2
HEYL

HFE
HFE2
HFM1
HGC6.3
HGD
HGFAC
HGS
HGSNAT
HHAT
HHATL
HHEX
HHIP
HHIPL1
HHIPL2
HHLA1
HHLA2
HHLA3
HIAT1
HIATL1
HIATL2
HIBADH
HIBCH
HIC1
HIC2
HID1
HID1-AS1
HIF1A
HIF1A-AS1
HIF1AN
HIF3A
HIGD1A
HIGD1B
HINFP
HINT1
HINT2
HINT3
HIP1
HIP1R
HIPK1
HIPK2
HIPK3
HIPK4
HIRA
HIRIP3
HISPPD2A
HIST1H1A
HIST1H1B
HIST1H1C
HIST1H1D
HIST1H1E

HIST1H2AA
HIST1H2AC
HIST1H2AD
HIST1H2AE
HIST1H2AG
HIST1H2AI
HIST1H2AJ
HIST1H2AM
HIST1H2BD
HIST1H2BE
HIST1H2BH
HIST1H2BK
HIST1H2BL
HIST1H2BN
HIST1H3B
HIST1H3D
HIST1H3E
HIST1H3I
HIST1H3J
HIST1H4A
HIST1H4B
HIST1H4E
HIST1H4F
HIST1H4G
HIST1H4H
HIST1H4J
HIST1H4K
HIST1H4L
HIST2H2AB
HIST2H2AC
HIST2H2BE
HIST2H2BF
HIST3H2A
HIST3H2BB
HIST3H3
HIST4H4
HIVEP1
HIVEP2
HIVEP3
HJURP
HK1
HK2
HK3
HKDC1
HKR1
HLA-A
HLA-B
HLA-C
HLA-DMA
HLA-DMB

HLA-DOA
HLA-DOB
HLA-DPA1
HLA-DPB1
HLA-DPB2
HLA-DQA1
HLA-DQA2
HLA-DQB1
HLA-DQB2
HLA-DRA
HLA-DRB1
HLA-DRB5
HLA-DRB6
HLA-E
HLA-F
HLA-G
HLA-H
HLA-J
HLA-L
HLCS
HLF
HLTF
HLX
HLX-AS1
HM13
HM13-AS1
HMBOX1
HMBS
HMCES
HMCN1
HMCN2
HMG20A
HMG20B
HMGA1
HMGA2
HMGB1
HMGB1L1
HMGB2
HMGB3
HMGB3L1
HMGB4
HMGCL
HMGCLL1
HMGCR
HMGCS1
HMGN1
HMGN2
HMGN2P46
HMGN3
HMGN3-AS1

HMGN4
HMGN5
HMGXB3
HMGXB4
HMHA1
HMHB1
HMMR
HMMR-AS1
HMOX1
HMOX2
HMP19
HMSD
HMX2
HMX3
HN1
HN1L
HNCAT21
HNF1A
HNF1B
HNF4A
HNF4A-AS1
HNF4G
HNMT
HNRNPA0
HNRNPA1
HNRNPA1L2
HNRNPA2B1
HNRNPA3
HNRNPA3P1
HNRNPAB
HNRNPC
HNRNPCL2
HNRNPD
HNRNPF
HNRNPH1
HNRNPH2
HNRNPH3
HNRNPK
HNRNPL
HNRNPLL
HNRNPM
HNRNPR
HNRNPU
HNRNPUL1
HNRNPUL2
HNRNPUL2-BSCL2
HNRPA1L-2
HNRPLL
HOGA1
HOMER1

HOMER2
HOMER3
HOMEZ
HOOK1
HOOK2
HOOK3
HOPX
HORMAD1
HORMAD2
HORMAD2-AS1
HOTAIR
HOTTIP
HOXA-AS3
HOXA1
HOXA10
HOXA10-AS
HOXA10-HOXA9
HOXA11
HOXA11-AS
HOXA11AS
HOXA13
HOXA2
HOXA3
HOXA4
HOXA5
HOXA6
HOXA7
HOXA9
HOXB-AS1
HOXB1
HOXB13
HOXB2
HOXB3
HOXB4
HOXB5
HOXB6
HOXB7
HOXB8
HOXB9
HOXC10
HOXC11
HOXC12
HOXC13
HOXC4
HOXC6
HOXC8
HOXC9
HOXD-AS2
HOXD1
HOXD10

HOXD11
HOXD12
HOXD13
HOXD3
HOXD4
HOXD8
HOXD9
HP09025
HP09053
HP1BP3
HPCA
HPCAL1
HPCAL4
HPD
HPDL
HPGD
HPGDS
HPN
HPN-AS1
HPR
HPRT1
HPS1
HPS3
HPS4
HPS5
HPS6
HPSE
HPSE2
HPVC1
HPX
HPYR1
HR
HRAS
HRASLS
HRASLS2
HRASLS5
HRAT17
HRAT5
HRAT56
HRAT92
HRC
HRCT1
HRG
HRH1
HRH2
HRH3
HRH4
HRK
HRNBP3
HRNR

HS1BP3
HS2ST1
HS3ST1
HS3ST2
HS3ST3A1
HS3ST3B1
HS3ST4
HS3ST5
HS3ST6
HS6ST1
HS6ST2
HS6ST3
HSBP1
HSBP1L1
HSCB
HSD11B1
HSD11B1L
HSD11B2
HSD17B1
HSD17B11
HSD17B12
HSD17B13
HSD17B14
HSD17B2
HSD17B3
HSD17B4
HSD17B7
HSD17B8
HSD3B1
HSD3B2
HSD3B7
HSD3BP4
HSDL1
HSDL2
HSF1
HSF2
HSF2BP
HSF4
HSF5
HSH2D
HSP90AA1
HSP90AB1
HSP90AB2P
HSP90B1
HSPA12A
HSPA12B
HSPA13
HSPA14
HSPA1A
HSPA1B

HSPA1L
HSPA2
HSPA4
HSPA4L
HSPA5
HSPA6
HSPA7
HSPA8
HSPA9
HSPB1
HSPB11
HSPB2
HSPB3
HSPB6
HSPB8
HSPB9
HSPBAP1
HSPBP1
HSPC072
HSPC157
HSPC159
HSPD1
HSPE1
HSPG2
HSPH1
HTA
HTATIP2
HTATSF1
HTATSF1P2
HTN3
HTR1A
HTR1B
HTR1D
HTR1E
HTR2A
HTR2B
HTR2C
HTR3A
HTR3B
HTR3C
HTR3D
HTR3E
HTR4
HTR5A
HTR5A-AS1
HTR6
HTR7
HTR7P
HTR7P1
HTRA1

HTRA2
HTRA3
HTRA4
HTT
HULC
HUNK
HUS1
HUS1B
HUWE1
HVCN1
HYAL1
HYAL2
HYAL3
HYAL4
HYALP1
HYDIN
HYI
HYKK
HYLS1
HYMAI
HYOU1
HYPK
IAH1
IAPP
IARS
IARS2
IBSP
IBTK
ICA1
ICA1L
ICAM1
ICAM2
ICAM3
ICAM4
ICAM5
ICE1
ICE2
ICK
ICMT
ICOS
ICOSLG
ICT1
ID1
ID2
ID2-AS1
ID2B
ID3
ID4
IDE
IDH1

IDH2
IDH3A
IDH3B
IDH3G
IDI1
IDI2
IDI2-AS1
IDNK
IDO1
IDO2
IDS
IDUA
IER2
IER3
IER3IP1
IER5
IER5L
IFFO1
IFFO2
IFI16
IFI27
IFI27L2
IFI30
IFI35
IFI44
IFI44L
IFI6
IFIH1
IFIT1
IFIT1L
IFIT2
IFIT3
IFIT5
IFITM1
IFITM10
IFITM2
IFITM5
IFLTD1
IFNA2
IFNA7
IFNAR2
IFNG
IFNG-AS1
IFNGR1
IFNGR2
IFNL4
IFNLR1
IFNW1
IFRD1
IFRD2

IFT122
IFT140
IFT172
IFT20
IFT27
IFT43
IFT52
IFT57
IFT74
IFT80
IFT81
IFT88
IGBP1
IGDCC3
IGDCC4
IGF1
IGF1R
IGF2
IGF2AS
IGF2BP1
IGF2BP2
IGF2BP3
IGF2R
IGFALS
IGFBP1
IGFBP2
IGFBP3
IGFBP4
IGFBP5
IGFBP6
IGFBP7
IGFBP7-AS1
IGFBPL1
IGFL1
IGFL2
IGFL3
IGFL4
IGFLR1
IGFN1
IGHMBP2
IGLL5
IGLON5
IGSF1
IGSF10
IGSF11
IGSF21
IGSF22
IGSF3
IGSF5
IGSF6

IGSF8
IGSF9
IGSF9B
IHH
IK
IKBKAP
IKBKB
IKBKE
IKBKG
IKZF1
IKZF2
IKZF3
IKZF4
IKZF5
IL10
IL10RA
IL10RB
IL11
IL12A
IL12A-AS1
IL12B
IL12RB1
IL12RB2
IL13
IL13RA1
IL13RA2
IL15
IL15RA
IL16
IL17C
IL17D
IL17F
IL17RA
IL17RB
IL17RC
IL17RD
IL17RE
IL17REL
IL18
IL18BP
IL18R1
IL18RAP
IL19
IL1A
IL1B
IL1F10
IL1F5
IL1F7
IL1F8
IL1F9

IL1R1
IL1R2
IL1RAP
IL1RAPL1
IL1RAPL2
IL1RL1
IL1RL2
IL1RN
IL20
IL20RA
IL20RB
IL21
IL21-AS1
IL21R
IL22
IL22RA1
IL22RA2
IL23A
IL23R
IL24
IL26
IL27
IL27RA
IL28B
IL29
IL2RA
IL2RB
IL2RG
IL3
IL31RA
IL32
IL33
IL34
IL36A
IL36B
IL36RN
IL4
IL4I1
IL4R
IL5
IL5RA
IL6R
IL6ST
IL7
IL7R
IL9
ILDR1
ILDR2
ILF2
ILF3

ILK
ILKAP
ILVBL
IMMP1L
IMMP2L
IMMT
IMP3
IMP4
IMPA1
IMPA2
IMPACT
IMPAD1
IMPDH1
IMPDH2
IMPG1
IMPG2
INA
INADL
INCA1
INCENP
INF2
ING1
ING2
ING3
ING4
ING5
INGX
INHBA
INHBB
INHBE
INO80
INO80B
INO80C
INO80E
INPP1
INPP4A
INPP4B
INPP5A
INPP5B
INPP5D
INPP5E
INPP5F
INPP5J
INPP5K
INPPL1
INS
INS-IGF2
INSC
INSIG1
INSIG2

INSL3
INSL5
INSL6
INSM1
INSM2
INSR
INSRR
INTS1
INTS10
INTS12
INTS2
INTS3
INTS4
INTS4L1
INTS5
INTS6
INTS7
INTS8
INTS9
INTU
INVS
IP6K1
IP6K2
IP6K3
IPCEF1
IPMK
IPO11
IPO13
IPO4
IPO5
IPO5P1
IPO7
IPO8
IPO9
IPP
IPPK
IQCA1
IQCA1L
IQCB1
IQCC
IQCD
IQCE
IQCF1
IQCF2
IQCF3
IQCF4
IQCF5
IQCF5-AS1
IQCF6
IQCG

IQCH
IQCH-AS1
IQCJ
IQCJ-SCHIP1
IQCK
IQGAP1
IQGAP2
IQGAP3
IQSEC1
IQSEC2
IQSEC3
IQUB
IRAK1
IRAK2
IRAK3
IRAK4
IRF1
IRF2
IRF2BP1
IRF2BP2
IRF2BPL
IRF3
IRF4
IRF5
IRF6
IRF7
IRF8
IRF9
IRGC
IRGM
IRS1
IRS2
IRS4
IRX1
IRX2
IRX3
IRX4
IRX5
IRX6
ISCA1
ISCU
ISG15
ISG20
ISG20L2
ISL1
ISL2
ISLR
ISLR2
ISM1
ISM2

ISOC1
ISOC2
ISPD
ISPD-AS1
IST1
ISX
ISY1
ISYNA1
ITCH
ITFG1
ITFG2
ITFG3
ITGA1
ITGA10
ITGA11
ITGA2
ITGA2B
ITGA3
ITGA4
ITGA5
ITGA6
ITGA7
ITGA8
ITGA9
ITGA9-AS1
ITGAD
ITGAE
ITGAL
ITGAM
ITGAV
ITGAX
ITGB1
ITGB1BP1
ITGB1BP2
ITGB1BP3
ITGB2
ITGB3
ITGB4
ITGB5
ITGB6
ITGB7
ITGB8
ITGBL1
ITIH1
ITIH2
ITIH3
ITIH4
ITIH4-AS1
ITIH5
ITIH5L

ITIH6
ITK
ITLN1
ITLN2
ITM2A
ITM2B
ITM2C
ITPA
ITPK1
ITPKA
ITPKB
ITPKB-IT1
ITPKC
ITPR1
ITPR2
ITPR3
ITPRIP
ITPRIPL1
ITPRIPL2
ITSN1
ITSN2
IVD
IVL
IVNS1ABP
IWS1
IYD
IZUMO1
IZUMO1R
IZUMO2
JADE1
JADE2
JADE3
JAG1
JAG2
JAGN1
JAK1
JAK2
JAK3
JAKMIP1
JAKMIP2
JAKMIP2-AS1
JAKMIP3
JAM2
JAM3
JARID2
JAZF1
JAZF1-AS1
JCHAIN
JDP2
JHDM1D

JKAMP
JMJD1C
JMJD1C-AS1
JMJD4
JMJD5
JMJD6
JMJD7
JMJD7-PLA2G4B
JMJD8
JMY
JOSD1
JOSD2
JPH1
JPH2
JPH3
JPH4
JPX
JRK
JRKL
JRKL-AS1
JSRP1
JTB
JUB
JUN
JUNB
JUND
JUP
KAAG1
KAL1
KALRN
KANK1
KANK2
KANK3
KANK4
KANSL1
KANSL1-AS1
KANSL1L
KANSL2
KANSL3
KANTR
KARS
KAT2A
KAT2B
KAT5
KAT6A
KAT6B
KAT7
KAT8
KATNA1
KATNAL1

KATNAL2
KATNB1
KAZALD1
KAZN
KBTBD10
KBTBD11
KBTBD11-OT1
KBTBD12
KBTBD2
KBTBD3
KBTBD4
KBTBD5
KBTBD6
KBTBD7
KC6
KCCAT198
KCCAT211
KCCAT333
KCMF1
KCNA1
KCNA10
KCNA2
KCNA3
KCNA4
KCNA5
KCNA6
KCNA7
KCNAB1
KCNAB1-AS2
KCNAB2
KCNAB3
KCNB1
KCNB2
KCNC1
KCNC2
KCNC3
KCNC4
KCNC4-AS1
KCND2
KCND3
KCND3-AS1
KCND3-IT1
KCNE1
KCNE3
KCNE4
KCNE5
KCNF1
KCNG1
KCNG2
KCNG3

KCNG4
KCNH1
KCNH2
KCNH3
KCNH4
KCNH5
KCNH6
KCNH7
KCNH8
KCNIP1
KCNIP2
KCNIP3
KCNIP4
KCNJ1
KCNJ10
KCNJ11
KCNJ12
KCNJ13
KCNJ14
KCNJ15
KCNJ16
KCNJ2
KCNJ3
KCNJ4
KCNJ5
KCNJ6
KCNJ8
KCNJ9
KCNK1
KCNK10
KCNK12
KCNK13
KCNK15
KCNK16
KCNK17
KCNK18
KCNK2
KCNK3
KCNK4
KCNK5
KCNK6
KCNK7
KCNK9
KCNMA1
KCNMA1-AS1
KCNMB1
KCNMB2
KCNMB3
KCNMB4
KCNN1

KCNN2
KCNN3
KCNN4
KCNQ1
KCNQ1DN
KCNQ1OT1
KCNQ2
KCNQ3
KCNQ4
KCNQ5
KCNQ5-AS1
KCNQ5-IT1
KCNRG
KCNS1
KCNS2
KCNS3
KCNT1
KCNT2
KCNU1
KCNV1
KCNV2
KCP
KCTD1
KCTD10
KCTD11
KCTD12
KCTD13
KCTD14
KCTD15
KCTD16
KCTD17
KCTD18
KCTD19
KCTD2
KCTD20
KCTD21
KCTD21-AS1
KCTD3
KCTD4
KCTD5
KCTD6
KCTD7
KCTD8
KCTD9
KDELR1
KDELR2
KDELR3
KDF1
KDM1A
KDM1B

KDM2A
KDM2B
KDM3A
KDM3B
KDM4A
KDM4B
KDM4C
KDM4D
KDM4DL
KDM5A
KDM5B
KDM5C
KDM6A
KDM6B
KDM7A
KDM8
KDR
KDSR
KEAP1
KEL
KERA
KHDC1
KHDC1L
KHDC3L
KHDRBS1
KHDRBS2
KHDRBS3
KHK
KHNYN
KHSRP
KIAA0020
KIAA0040
KIAA0087
KIAA0090
KIAA0100
KIAA0101
KIAA0125
KIAA0141
KIAA0146
KIAA0174
KIAA0182
KIAA0195
KIAA0196
KIAA0226
KIAA0226L
KIAA0232
KIAA0247
KIAA0284
KIAA0317
KIAA0319

KIAA0319L
KIAA0355
KIAA0368
KIAA0391
KIAA0406
KIAA0408
KIAA0415
KIAA0427
KIAA0430
KIAA0467
KIAA0494
KIAA0495
KIAA0513
KIAA0528
KIAA0556
KIAA0562
KIAA0564
KIAA0586
KIAA0649
KIAA0652
KIAA0664
KIAA0748
KIAA0753
KIAA0754
KIAA0776
KIAA0802
KIAA0825
KIAA0831
KIAA0892
KIAA0895
KIAA0895L
KIAA0907
KIAA0913
KIAA0922
KIAA0930
KIAA0947
KIAA1009
KIAA1012
KIAA1024
KIAA1024L
KIAA1026
KIAA1033
KIAA1045
KIAA1143
KIAA1147
KIAA1161
KIAA1191
KIAA1199
KIAA1210
KIAA1211

KIAA1211L
KIAA1217
KIAA1239
KIAA1244
KIAA1257
KIAA1267
KIAA1274
KIAA1279
KIAA1310
KIAA1324
KIAA1324L
KIAA1328
KIAA1383
KIAA1407
KIAA1409
KIAA1429
KIAA1430
KIAA1432
KIAA1456
KIAA1462
KIAA1467
KIAA1468
KIAA1486
KIAA1522
KIAA1524
KIAA1529
KIAA1530
KIAA1539
KIAA1543
KIAA1549
KIAA1549L
KIAA1551
KIAA1586
KIAA1609
KIAA1614
KIAA1632
KIAA1644
KIAA1671
KIAA1683
KIAA1688
KIAA1712
KIAA1715
KIAA1731
KIAA1751
KIAA1755
KIAA1797
KIAA1804
KIAA1826
KIAA1841
KIAA1875

KIAA1908
KIAA1919
KIAA1949
KIAA1958
KIAA1967
KIAA1984
KIAA2012
KIAA2018
KIAA2026
KIDINS220
KIF11
KIF12
KIF13A
KIF13B
KIF14
KIF15
KIF16B
KIF17
KIF18A
KIF18B
KIF19
KIF1A
KIF1B
KIF1C
KIF20B
KIF21A
KIF21B
KIF22
KIF23
KIF24
KIF25
KIF26A
KIF26B
KIF27
KIF2B
KIF2C
KIF3A
KIF3B
KIF3C
KIF4A
KIF4B
KIF5A
KIF5B
KIF5C
KIF6
KIF7
KIF9
KIF9-AS1
KIFAP3
KIFC1

KIFC2
KIFC3
KIN
KIR2DL3
KIR2DL4
KIR3DL2
KIR3DL3
KIR3DP1
KIR3DX1
KIRREL
KIRREL2
KIRREL3
KIRREL3-AS2
KIRREL3-AS3
KISS1
KISS1R
KIT
KITLG
KIZ
KL
KLB
KLC1
KLC2
KLC3
KLC4
KLF1
KLF10
KLF11
KLF12
KLF13
KLF14
KLF15
KLF16
KLF17
KLF2
KLF3
KLF5
KLF6
KLF7
KLF8
KLF9
KLHDC1
KLHDC10
KLHDC2
KLHDC3
KLHDC4
KLHDC5
KLHDC7A
KLHDC7B
KLHDC8A

KLHDC8B
KLHDC9
KLHL1
KLHL10
KLHL11
KLHL12
KLHL13
KLHL14
KLHL15
KLHL17
KLHL18
KLHL2
KLHL21
KLHL22
KLHL23
KLHL24
KLHL25
KLHL26
KLHL28
KLHL29
KLHL3
KLHL30
KLHL31
KLHL32
KLHL33
KLHL34
KLHL35
KLHL36
KLHL38
KLHL4
KLHL41
KLHL42
KLHL5
KLHL6
KLHL6-AS1
KLHL7
KLHL7-AS1
KLHL8
KLHL9
KLK1
KLK10
KLK11
KLK12
KLK13
KLK14
KLK15
KLK2
KLK3
KLK4
KLK5

KLK6
KLK7
KLKB1
KLKBL4
KLKP1
KLRA1
KLRAQ1
KLRC1
KLRC2
KLRD1
KLRF2
KLRG1
KLRG2
KLRK1
KMO
KMT2A
KMT2B
KMT2C
KMT2D
KMT2E
KMT2E-AS1
KNCN
KNDC1
KNOP1
KNSTRN
KNTC1
KPNA1
KPNA2
KPNA3
KPNA4
KPNA5
KPNA6
KPNA7
KPNB1
KPRP
KPTN
KRAS
KRBA1
KRBA2
KRBOX1
KRBOX4
KRCC1
KREMEN1
KREMEN2
KRI1
KRIT1
KRR1
KRT1
KRT12
KRT13

KRT14
KRT16
KRT17
KRT18
KRT18P55
KRT19
KRT20
KRT222
KRT25
KRT26
KRT27
KRT28
KRT31
KRT32
KRT33A
KRT33B
KRT34
KRT36
KRT37
KRT38
KRT39
KRT4
KRT40
KRT42P
KRT6A
KRT7
KRT71
KRT72
KRT74
KRT76
KRT77
KRT78
KRT79
KRT8
KRT80
KRT81
KRT82
KRT83
KRT84
KRT85
KRT86
KRT9
KRTAP1-1
KRTAP1-4
KRTAP1-5
KRTAP10-1
KRTAP10-11
KRTAP10-12
KRTAP10-2
KRTAP10-3

KRTAP10-4
KRTAP10-5
KRTAP10-6
KRTAP10-9
KRTAP11-1
KRTAP12-2
KRTAP12-3
KRTAP13-2
KRTAP13-3
KRTAP13-4
KRTAP17-1
KRTAP19-2
KRTAP19-3
KRTAP19-5
KRTAP19-8
KRTAP2-1
KRTAP2-2
KRTAP20-4
KRTAP21-2
KRTAP21-3
KRTAP23-1
KRTAP24-1
KRTAP25-1
KRTAP27-1
KRTAP29-1
KRTAP3-1
KRTAP3-3
KRTAP4-12
KRTAP4-3
KRTAP4-5
KRTAP4-6
KRTAP4-7
KRTAP4-9
KRTAP5-10
KRTAP5-2
KRTAP5-3
KRTAP5-8
KRTAP6-2
KRTAP6-3
KRTAP7-1
KRTAP9-1
KRTAP9-2
KRTAP9-4
KRTAP9-9
KRTCAP3
KRTDAP
KSR1
KSR2
KTELC1
KTI12

KTN1
KTN1-AS1
KU-MEL-3
KXD1
KY
KYNU
L1CAM
L1TD1
L2HGDH
L3MBTL
L3MBTL1
L3MBTL2
L3MBTL3
L3MBTL4
LACAT8
LACC1
LACE1
LACRT
LACTB
LACTB2
LACTB2-AS1
LAD1
LAG3
LAGE3
LAIR1
LAIR2
LAMA1
LAMA2
LAMA3
LAMA4
LAMA5
LAMB1
LAMB2
LAMB3
LAMB4
LAMC1
LAMC2
LAMC3
LAMP1
LAMP2
LAMP3
LAMP5
LAMP5-AS1
LAMTOR1
LAMTOR3
LAMTOR5-AS1
LANCL1
LANCL1-AS1
LANCL2
LANCL3

LAP3
LAPTM4A
LAPTM4B
LAPTM5
LARGE
LARP1
LARP1B
LARP4
LARP4B
LARP6
LARP7
LARS2
LARS2-AS1
LAS1L
LASP1
LASS2
LASS3
LASS4
LASS5
LASS6
LAT
LAT2
LATS1
LATS2
LAX1
LAYN
LBH
LBHD1
LBP
LBR
LBX1
LBX1-AS1
LBX2
LBX2-AS1
LBXCOR1
LCA5
LCA5L
LCE1A
LCE1B
LCE1E
LCE1F
LCE2B
LCE2D
LCE3A
LCE3E
LCE4A
LCE5A
LCK
LCLAT1
LCMT1

LCMT1-AS1
LCMT1-AS2
LCMT2
LCN1
LCN10
LCN12
LCN15
LCN6
LCN8
LCN9
LCNL1
LCOR
LCORL
LCP1
LCP2
LCT
LCTL
LDAH
LDB1
LDB2
LDB3
LDHA
LDHAL6A
LDHAL6B
LDHB
LDHC
LDHD
LDLR
LDLRAD1
LDLRAD2
LDLRAD3
LDLRAD4
LDLRAP1
LDOC1
LDOC1L
LEAP2
LECT1
LECT2
LEF1
LEF1-AS1
LEFTY1
LEFTY2
LEKR1
LELP1
LEMD1
LEMD2
LEMD3
LENG1
LENG8
LENG8-AS1

LENG9
LEO1
LEP
LEPR
LEPRE1
LEPREL1
LEPREL2
LEPROT
LEPROTL1
LETM1
LETM2
LETMD1
LEUTX
LFNG
LGALS1
LGALS12
LGALS13
LGALS14
LGALS16
LGALS17A
LGALS3
LGALS3BP
LGALS4
LGALS7
LGALS8
LGALS9
LGI1
LGI2
LGI3
LGI4
LGMN
LGR4
LGR5
LGR6
LGSN
LHB
LHCGR
LHFP
LHFPL1
LHFPL2
LHFPL3
LHFPL3-AS2
LHFPL4
LHFPL5
LHPP
LHX1
LHX2
LHX3
LHX4
LHX4-AS1

LHX5
LHX5-AS1
LHX6
LHX8
LHX9
LIAS
LIF
LIFR
LIFR-AS1
LIG1
LIG3
LIG4
LILRA1
LILRA2
LILRA3
LILRA4
LILRB1
LILRB2
LILRB3
LILRB4
LILRB5
LIM2
LIMA1
LIMCH1
LIMD1
LIMD2
LIME1
LIMK1
LIMK2
LIMS1
LIMS2
LIMS3-LOC440895
LIN28
LIN28B
LIN37
LIN52
LIN54
LIN7A
LIN7B
LIN7C
LIN9
LINC-PINT
LINC00029
LINC00051
LINC00092
LINC00094
LINC00113
LINC00152
LINC00158
LINC00160

LINC00161
LINC00174
LINC00176
LINC00184
LINC00189
LINC00200
LINC00202-1
LINC00207
LINC00210
LINC00211
LINC00222
LINC00229
LINC00238
LINC00239
LINC00242
LINC00243
LINC00251
LINC00261
LINC00269
LINC00271
LINC00273
LINC00276
LINC00282
LINC00284
LINC00298
LINC00299
LINC00301
LINC00305
LINC00310
LINC00313
LINC00323
LINC00327
LINC00331
LINC00332
LINC00333
LINC00341
LINC00343
LINC00350
LINC00351
LINC00354
LINC00359
LINC00365
LINC00366
LINC00368
LINC00371
LINC00375
LINC00376
LINC00379
LINC00380
LINC00381

LINC00382
LINC00392
LINC00395
LINC00396
LINC00398
LINC00400
LINC00403
LINC00410
LINC00411
LINC00423
LINC00424
LINC00426
LINC00431
LINC00437
LINC00440
LINC00442
LINC00443
LINC00445
LINC00446
LINC00448
LINC00456
LINC00457
LINC00460
LINC00461
LINC00462
LINC00466
LINC00469
LINC00470
LINC00472
LINC00473
LINC00474
LINC00475
LINC00476
LINC00485
LINC00486
LINC00487
LINC00488
LINC00489
LINC00494
LINC00499
LINC00501
LINC00502
LINC00504
LINC00507
LINC00511
LINC00518
LINC00521
LINC00523
LINC00524
LINC00525

LINC00534
LINC00535
LINC00536
LINC00544
LINC00545
LINC00554
LINC00557
LINC00558
LINC00559
LINC00562
LINC00563
LINC00564
LINC00570
LINC00577
LINC00578
LINC00581
LINC00582
LINC00589
LINC00592
LINC00593
LINC00595
LINC00598
LINC00605
LINC00606
LINC00607
LINC00609
LINC00613
LINC00615
LINC00616
LINC00618
LINC00619
LINC00620
LINC00623
LINC00624
LINC00629
LINC00632
LINC00635
LINC00638
LINC00639
LINC00642
LINC00643
LINC00644
LINC00648
LINC00649
LINC00658
LINC00661
LINC00662
LINC00665
LINC00669
LINC00670

LINC00671
LINC00673
LINC00674
LINC00681
LINC00683
LINC00687
LINC00689
LINC00692
LINC00693
LINC00700
LINC00701
LINC00702
LINC00703
LINC00704
LINC00705
LINC00707
LINC00709
LINC00710
LINC00824
LINC00836
LINC00840
LINC00841
LINC00842
LINC00844
LINC00845
LINC00850
LINC00851
LINC00853
LINC00857
LINC00861
LINC00862
LINC00866
LINC00870
LINC00871
LINC00877
LINC00880
LINC00883
LINC00886
LINC00887
LINC00888
LINC00892
LINC00896
LINC00898
LINC00901
LINC00907
LINC00908
LINC00910
LINC00911
LINC00917
LINC00920

LINC00922
LINC00923
LINC00924
LINC00925
LINC00926
LINC00927
LINC00929
LINC00930
LINC00935
LINC00937
LINC00939
LINC00940
LINC00942
LINC00944
LINC00945
LINC00950
LINC00951
LINC00955
LINC00958
LINC00959
LINC00961
LINC00963
LINC00964
LINC00967
LINC00968
LINC00969
LINC00970
LINC00974
LINC00977
LINC00992
LINC00993
LINC00994
LINC00998
LINC01001
LINC01004
LINC01005
LINC01006
LINC01007
LINC01010
LINC01013
LINC01014
LINC01015
LINC01016
LINC01017
LINC01018
LINC01019
LINC01020
LINC01021
LINC01023
LINC01024

LINC01029
LINC01031
LINC01033
LINC01036
LINC01046
LINC01050
LINC01053
LINC01057
LINC01058
LINC01060
LINC01061
LINC01063
LINC01065
LINC01069
LINC01075
LINC01081
LINC01085
LINC01087
LINC01088
LINC01089
LINC01090
LINC01091
LINC01094
LINC01095
LINC01096
LINC01098
LINC01099
LINC01100
LINC01102
LINC01103
LINC01104
LINC01105
LINC01107
LINC01108
LINC01111
LINC01114
LINC01115
LINC01116
LINC01117
LINC01119
LINC01121
LINC01122
LINC01125
LINC01126
LINC01128
LINC01133
LINC01134
LINC01135
LINC01140
LINC01141

LINC01146
LINC01151
LINC01152
LINC01159
LINC01160
LINC01163
LINC01164
LINC01167
LINC01169
LINC01170
LINC01176
LINC01177
LINC01179
LINC01182
LINC01184
LINC01185
LINC01187
LINC01191
LINC01192
LINC01193
LINC01194
LINC01195
LINC01197
LINC01198
LINC01201
LINC01202
LINC01204
LINC01205
LINC01207
LINC01208
LINC01210
LINC01213
LINC01214
LINC01215
LINC01216
LINC01221
LINC01225
LINC01226
LINC01227
LINC01229
LINC01230
LINC01231
LINC01232
LINC01237
LINC01239
LINC01241
LINC01246
LINC01248
LINC01249
LINC01250

LINC01252
LINC01255
LINC01256
LINC01257
LINC01258
LINC01262
LINC01264
LINC01269
LINC01270
LINC01271
LINC01272
LINC01273
LINC01277
LINC01278
LINC01281
LINC01285
LINC01288
LINC01289
LINC01291
LINC01296
LINC01298
LINC01304
LINC01305
LINC01307
LINC01309
LINC01310
LINC01314
LINC01317
LINC01320
LINC01322
LINC01324
LINC01327
LINC01331
LINC01335
LINC01337
LINC01338
LINC01339
LINC01340
LINC01341
LINC01342
LINC01343
LINC01344
LINC01346
LINC01349
LINC01350
LINC01352
LINC01353
LINC01356
LINC01358
LINC01360

LINC01375
LINC01377
LINC01378
LINC01384
LINC01387
LINC01389
LINC01391
LINC01392
LINC01393
LINC01397
LINC01398
LINC01399
LINC01410
LINC01411
LINC01412
LINC01413
LINC01416
LINC01420
LINC01422
LINC01426
LINC01427
LINC01428
LINC01429
LINC01430
LINC01435
LINC01441
LINC01443
LINC01446
LINC01447
LINC01448
LINC01449
LINC01455
LINC01461
LINC01467
LINC01470
LINC01471
LINC01475
LINC01476
LINC01477
LINC01478
LINC01479
LINC01480
LINC01483
LINC01484
LINC01485
LINC01487
LINC01490
LINC01491
LINC01492
LINC01493

LINC01494
LINC01495
LINC01496
LINC01499
LINC01500
LINC01501
LINC01502
LINC01504
LINC01505
LINC01506
LINC01507
LINC01508
LINC01509
LINC01510
LINC01511
LINC01512
LINC01515
LINC01517
LINC01518
LINC01522
LINC01524
LINC01526
LINC01529
LINC01530
LINC01531
LINC01532
LINC01535
LINC01538
LINC01539
LINC01541
LINC01544
LINC01546
LINC01547
LINC01549
LINC01551
LINC01552
LINC01554
LINC01555
LINC01556
LINC01558
LINC01563
LINC01565
LINC01567
LINC01570
LINC01571
LINC01572
LINC01573
LINC01578
LINC01580
LINC01581

LINC01582
LINC01583
LINC01584
LINC01586
LINC01587
LINC01592
LINC01599
LINC01603
LINC01604
LINC01605
LINC01607
LINC01609
LINCR-0001
LINCR-0002
LINGO1
LINGO1-AS1
LINGO1-AS2
LINGO2
LINGO3
LINGO4
LINS
LIPA
LIPC
LIPE
LIPE-AS1
LIPF
LIPG
LIPH
LIPI
LIPK
LIPN
LIPT1
LIPT2
LITAF
LIX1
LIX1L
LKAAEAR1
LL22NC03-75H12.2
LLGL1
LLGL2
LLPH
LMAN1
LMAN1L
LMAN2
LMAN2L
LMBR1
LMBR1L
LMBRD1
LMBRD2
LMCD1

LMCD1-AS1
LMF1
LMF1-AS1
LMF2
LMLN
LMNA
LMNB1
LMNB2
LMNTD1
LMNTD2
LMO1
LMO2
LMO3
LMO4
LMO7
LMO7-AS1
LMO7DN
LMO7DN-IT1
LMOD1
LMOD2
LMOD3
LMTK2
LMTK3
LMX1A
LMX1B
LNP1
LNPEP
LNX1
LNX2
LOC100101121
LOC100101938
LOC100124692
LOC100125556
LOC100126784
LOC100127888
LOC100128006
LOC100128023
LOC100128071
LOC100128076
LOC100128164
LOC100128233
LOC100128239
LOC100128288
LOC100128292
LOC100128317
LOC100128398
LOC100128531
LOC100128542
LOC100128554
LOC100128568

LOC100128593
LOC100128640
LOC100128675
LOC100128714
LOC100128731
LOC100128770
LOC100128788
LOC100128811
LOC100128822
LOC100128977
LOC100128993
LOC100129027
LOC100129034
LOC100129046
LOC100129066
LOC100129138
LOC100129148
LOC100129175
LOC100129203
LOC100129216
LOC100129354
LOC100129461
LOC100129550
LOC100129603
LOC100129617
LOC100129637
LOC100129697
LOC100129716
LOC100129924
LOC100129935
LOC100129940
LOC100130015
LOC100130017
LOC100130093
LOC100130238
LOC100130264
LOC100130274
LOC100130331
LOC100130357
LOC100130370
LOC100130417
LOC100130451
LOC100130476
LOC100130522
LOC100130581
LOC100130691
LOC100130700
LOC100130776
LOC100130872
LOC100130872-SPON2

LOC100130880
LOC100130899
LOC100130933
LOC100130964
LOC100130987
LOC100131047
LOC100131289
LOC100131303
LOC100131347
LOC100131434
LOC100131551
LOC100131564
LOC100131655
LOC100132111
LOC100132146
LOC100132163
LOC100132174
LOC100132215
LOC100132354
LOC100132356
LOC100132735
LOC100132781
LOC100132831
LOC100133091
LOC100133286
LOC100133308
LOC100133461
LOC100133469
LOC100133545
LOC100133612
LOC100133669
LOC100133893
LOC100133957
LOC100133991
LOC100134229
LOC100134259
LOC100134317
LOC100134368
LOC100134713
LOC100134868
LOC100144595
LOC100144603
LOC100144604
LOC100169752
LOC100188947
LOC100189589
LOC100190938
LOC100190939
LOC100190940
LOC100192378

LOC100192379
LOC100192426
LOC100216001
LOC100240735
LOC100270710
LOC100270746
LOC100271715
LOC100271836
LOC100272146
LOC100272217
LOC100272228
LOC100286844
LOC100286922
LOC100286948
LOC100287015
LOC100287036
LOC100287216
LOC100287225
LOC100287592
LOC100287718
LOC100287792
LOC100287944
LOC100288152
LOC100288181
LOC100288637
LOC100288797
LOC100288798
LOC100288866
LOC100289333
LOC100289341
LOC100289473
LOC100289580
LOC100289650
LOC100289673
LOC100292680
LOC100294362
LOC100302401
LOC100302640
LOC100302652
LOC100303728
LOC100306951
LOC100329108
LOC100419583
LOC100420587
LOC100422737
LOC100499194
LOC100499484
LOC100499484-C9ORF174
LOC100505478
LOC100505530

LOC100505549
LOC100505622
LOC100505625
LOC100505658
LOC100505795
LOC100505817
LOC100505841
LOC100505918
LOC100505920
LOC100505938
LOC100505942
LOC100505978
LOC100506022
LOC100506023
LOC100506071
LOC100506082
LOC100506083
LOC100506122
LOC100506124
LOC100506127
LOC100506142
LOC100506175
LOC100506178
LOC100506207
LOC100506258
LOC100506271
LOC100506272
LOC100506274
LOC100506281
LOC100506314
LOC100506368
LOC100506384
LOC100506393
LOC100506403
LOC100506406
LOC100506422
LOC100506457
LOC100506470
LOC100506474
LOC100506476
LOC100506497
LOC100506526
LOC100506551
LOC100506606
LOC100506639
LOC100506682
LOC100506688
LOC100506700
LOC100506725
LOC100506790

LOC100506801
LOC100506834
LOC100506844
LOC100506860
LOC100506869
LOC100506895
LOC100506985
LOC100507002
LOC100507065
LOC100507073
LOC100507140
LOC100507156
LOC100507194
LOC100507195
LOC100507205
LOC100507250
LOC100507291
LOC100507351
LOC100507373
LOC100507377
LOC100507387
LOC100507389
LOC100507391
LOC100507406
LOC100507424
LOC100507431
LOC100507462
LOC100507477
LOC100507487
LOC100507537
LOC100507557
LOC100507577
LOC100507639
LOC100507642
LOC100631378
LOC100652768
LOC100996291
LOC100996324
LOC100996338
LOC100996342
LOC100996455
LOC100996579
LOC100996583
LOC100996634
LOC100996635
LOC100996654
LOC100996671
LOC100996693
LOC100996694
LOC101060019

LOC101060091
LOC101060321
LOC101060385
LOC101060498
LOC101448202
LOC101593348
LOC101805491
LOC101926892
LOC101926908
LOC101926913
LOC101926941
LOC101926942
LOC101926944
LOC101926955
LOC101926963
LOC101926964
LOC101926966
LOC101927020
LOC101927021
LOC101927023
LOC101927027
LOC101927040
LOC101927043
LOC101927045
LOC101927053
LOC101927056
LOC101927058
LOC101927066
LOC101927069
LOC101927078
LOC101927079
LOC101927123
LOC101927132
LOC101927143
LOC101927153
LOC101927156
LOC101927157
LOC101927159
LOC101927164
LOC101927179
LOC101927181
LOC101927189
LOC101927211
LOC101927229
LOC101927230
LOC101927237
LOC101927239
LOC101927244
LOC101927267
LOC101927282

LOC101927284
LOC101927285
LOC101927286
LOC101927292
LOC101927310
LOC101927314
LOC101927322
LOC101927334
LOC101927342
LOC101927354
LOC101927358
LOC101927359
LOC101927378
LOC101927379
LOC101927394
LOC101927410
LOC101927412
LOC101927415
LOC101927416
LOC101927418
LOC101927419
LOC101927421
LOC101927434
LOC101927437
LOC101927438
LOC101927450
LOC101927460
LOC101927464
LOC101927482
LOC101927488
LOC101927495
LOC101927497
LOC101927501
LOC101927502
LOC101927523
LOC101927526
LOC101927543
LOC101927550
LOC101927560
LOC101927571
LOC101927580
LOC101927583
LOC101927587
LOC101927588
LOC101927592
LOC101927604
LOC101927606
LOC101927616
LOC101927620
LOC101927630

LOC101927637
LOC101927641
LOC101927650
LOC101927653
LOC101927657
LOC101927661
LOC101927668
LOC101927691
LOC101927692
LOC101927694
LOC101927708
LOC101927730
LOC101927735
LOC101927746
LOC101927762
LOC101927765
LOC101927766
LOC101927768
LOC101927769
LOC101927780
LOC101927787
LOC101927797
LOC101927798
LOC101927811
LOC101927814
LOC101927815
LOC101927817
LOC101927822
LOC101927835
LOC101927843
LOC101927844
LOC101927845
LOC101927847
LOC101927849
LOC101927854
LOC101927865
LOC101927869
LOC101927870
LOC101927876
LOC101927901
LOC101927911
LOC101927914
LOC101927934
LOC101927950
LOC101927954
LOC101927964
LOC101927967
LOC101927969
LOC101927973
LOC101927987

LOC101928008
LOC101928009
LOC101928020
LOC101928068
LOC101928093
LOC101928100
LOC101928103
LOC101928107
LOC101928134
LOC101928135
LOC101928137
LOC101928161
LOC101928162
LOC101928167
LOC101928174
LOC101928191
LOC101928203
LOC101928211
LOC101928223
LOC101928227
LOC101928231
LOC101928241
LOC101928253
LOC101928254
LOC101928259
LOC101928273
LOC101928279
LOC101928298
LOC101928303
LOC101928306
LOC101928314
LOC101928324
LOC101928371
LOC101928372
LOC101928401
LOC101928402
LOC101928404
LOC101928414
LOC101928417
LOC101928418
LOC101928437
LOC101928438
LOC101928441
LOC101928443
LOC101928445
LOC101928449
LOC101928453
LOC101928460
LOC101928461
LOC101928489

LOC101928509
LOC101928516
LOC101928517
LOC101928535
LOC101928539
LOC101928565
LOC101928569
LOC101928580
LOC101928590
LOC101928605
LOC101928618
LOC101928626
LOC101928650
LOC101928651
LOC101928673
LOC101928682
LOC101928694
LOC101928697
LOC101928700
LOC101928708
LOC101928710
LOC101928731
LOC101928737
LOC101928739
LOC101928748
LOC101928767
LOC101928769
LOC101928778
LOC101928782
LOC101928790
LOC101928791
LOC101928794
LOC101928812
LOC101928841
LOC101928851
LOC101928861
LOC101928880
LOC101928882
LOC101928885
LOC101928886
LOC101928891
LOC101928894
LOC101928896
LOC101928911
LOC101928936
LOC101928937
LOC101928940
LOC101928942
LOC101928944
LOC101928961

LOC101928977
LOC101928978
LOC101928989
LOC101928992
LOC101928994
LOC101929019
LOC101929023
LOC101929057
LOC101929058
LOC101929073
LOC101929089
LOC101929095
LOC101929106
LOC101929128
LOC101929140
LOC101929147
LOC101929153
LOC101929154
LOC101929162
LOC101929164
LOC101929181
LOC101929194
LOC101929199
LOC101929217
LOC101929224
LOC101929229
LOC101929231
LOC101929237
LOC101929241
LOC101929260
LOC101929268
LOC101929279
LOC101929284
LOC101929294
LOC101929295
LOC101929297
LOC101929312
LOC101929315
LOC101929319
LOC101929331
LOC101929337
LOC101929372
LOC101929378
LOC101929380
LOC101929412
LOC101929420
LOC101929427
LOC101929439
LOC101929448
LOC101929450

LOC101929452
LOC101929454
LOC101929468
LOC101929470
LOC101929486
LOC101929488
LOC101929505
LOC101929517
LOC101929524
LOC101929532
LOC101929541
LOC101929549
LOC101929550
LOC101929551
LOC101929555
LOC101929563
LOC101929565
LOC101929567
LOC101929570
LOC101929572
LOC101929584
LOC101929586
LOC101929595
LOC101929596
LOC101929608
LOC101929622
LOC101929625
LOC101929626
LOC101929631
LOC101929653
LOC101929660
LOC101929662
LOC101929680
LOC101929681
LOC101929694
LOC101929698
LOC101929705
LOC101929709
LOC101929710
LOC101929717
LOC101929718
LOC101929721
LOC101929723
LOC101929753
LOC101929754
LOC101929762
LOC101929897
LOC101930010
LOC101930452
LOC102467079

LOC102467080
LOC102467081
LOC102467147
LOC102467213
LOC102467216
LOC102467217
LOC102467222
LOC102467224
LOC102467225
LOC102467655
LOC102477328
LOC102503427
LOC102546226
LOC102546229
LOC102546294
LOC102546299
LOC102577426
LOC102723330
LOC102723344
LOC102723354
LOC102723362
LOC102723376
LOC102723385
LOC102723427
LOC102723439
LOC102723505
LOC102723582
LOC102723649
LOC102723701
LOC102723709
LOC102723729
LOC102723824
LOC102723828
LOC102723831
LOC102723833
LOC102723854
LOC102723895
LOC102724000
LOC102724020
LOC102724050
LOC102724058
LOC102724084
LOC102724094
LOC102724153
LOC102724163
LOC102724188
LOC102724190
LOC102724201
LOC102724297
LOC102724421

LOC102724467
LOC102724511
LOC102724552
LOC102724555
LOC102724589
LOC102724596
LOC102724601
LOC102724612
LOC102724623
LOC102724651
LOC102724663
LOC102724691
LOC102724699
LOC102724710
LOC102724784
LOC102724804
LOC102724849
LOC102724890
LOC102724919
LOC102724933
LOC102724957
LOC102800447
LOC103312105
LOC103344931
LOC103611081
LOC104054148
LOC104968399
LOC105274304
LOC105375734
LOC105376360
LOC105376671
LOC105378732
LOC105447648
LOC105616981
LOC105667213
LOC105747689
LOC113230
LOC115110
LOC121838
LOC134466
LOC139201
LOC143666
LOC144571
LOC144776
LOC145783
LOC145814
LOC145820
LOC145845
LOC146336
LOC146513

LOC146880
LOC147804
LOC148145
LOC148189
LOC148696
LOC148709
LOC149134
LOC149373
LOC149837
LOC149950
LOC150185
LOC150197
LOC150381
LOC150527
LOC150568
LOC150622
LOC150786
LOC150935
LOC151174
LOC151484
LOC151534
LOC151658
LOC152024
LOC152225
LOC152578
LOC153328
LOC153684
LOC153910
LOC154449
LOC154761
LOC154822
LOC157273
LOC157627
LOC158376
LOC158381
LOC158434
LOC158435
LOC158572
LOC162632
LOC169834
LOC200726
LOC200772
LOC201651
LOC202181
LOC202781
LOC219347
LOC220429
LOC220729
LOC221442
LOC221710

LOC221946
LOC222699
LOC253573
LOC253724
LOC254028
LOC254312
LOC254559
LOC254896
LOC255025
LOC255167
LOC255411
LOC255512
LOC256880
LOC257396
LOC25845
LOC282997
LOC283038
LOC283050
LOC283140
LOC283177
LOC283194
LOC283214
LOC283267
LOC283299
LOC283352
LOC283392
LOC283404
LOC283440
LOC283575
LOC283585
LOC283663
LOC283683
LOC283761
LOC283856
LOC283867
LOC283922
LOC283999
LOC284009
LOC284023
LOC284100
LOC284232
LOC284233
LOC284276
LOC284294
LOC284344
LOC284395
LOC284441
LOC284551
LOC284661
LOC284688

LOC284749
LOC284788
LOC284798
LOC284805
LOC284825
LOC284930
LOC284933
LOC284950
LOC285043
LOC285045
LOC285074
LOC285205
LOC285359
LOC285370
LOC285375
LOC285401
LOC285419
LOC285456
LOC285484
LOC285501
LOC285548
LOC285550
LOC285593
LOC285626
LOC285629
LOC285692
LOC285696
LOC285740
LOC285762
LOC285766
LOC285768
LOC285780
LOC285796
LOC285830
LOC285847
LOC285954
LOC286002
LOC286016
LOC286059
LOC286083
LOC286094
LOC286135
LOC286238
LOC286297
LOC286359
LOC286370
LOC286467
LOC338694
LOC338758
LOC338797

LOC338799
LOC339166
LOC339240
LOC339260
LOC339524
LOC339529
LOC339535
LOC339568
LOC339593
LOC339622
LOC339666
LOC339674
LOC339803
LOC339862
LOC339975
LOC340074
LOC340094
LOC340107
LOC340113
LOC340357
LOC340508
LOC342346
LOC344595
LOC344887
LOC344967
LOC348021
LOC348840
LOC348926
LOC349114
LOC360030
LOC374443
LOC375196
LOC387646
LOC387763
LOC387810
LOC388282
LOC388387
LOC388406
LOC388428
LOC388436
LOC388692
LOC388789
LOC388796
LOC388813
LOC388849
LOC388942
LOC388946
LOC389033
LOC389247
LOC389333

LOC389458
LOC389493
LOC389602
LOC389634
LOC389641
LOC389765
LOC389791
LOC390594
LOC390595
LOC390858
LOC391003
LOC391322
LOC399815
LOC399886
LOC399959
LOC400043
LOC400578
LOC400655
LOC400657
LOC400685
LOC400696
LOC400752
LOC400794
LOC400804
LOC400867
LOC400891
LOC400927
LOC400927-CSNK1E
LOC400931
LOC400940
LOC400958
LOC400997
LOC401052
LOC401097
LOC401127
LOC401177
LOC401286
LOC401312
LOC401320
LOC401324
LOC401387
LOC401463
LOC401557
LOC401629
LOC402377
LOC402644
LOC404266
LOC407835
LOC439933
LOC440028

LOC440040
LOC440311
LOC440335
LOC440356
LOC440434
LOC440446
LOC440461
LOC440563
LOC440600
LOC440839
LOC440895
LOC440905
LOC440910
LOC440925
LOC440944
LOC440957
LOC440982
LOC441025
LOC441046
LOC441089
LOC441177
LOC441204
LOC441455
LOC441601
LOC441666
LOC441869
LOC441897
LOC442245
LOC442421
LOC442459
LOC493754
LOC494141
LOC51145
LOC541472
LOC550112
LOC552889
LOC553103
LOC55338
LOC554203
LOC55908
LOC595101
LOC613266
LOC619207
LOC63930
LOC641367
LOC641518
LOC642006
LOC642361
LOC642366
LOC642587

LOC642597
LOC642846
LOC642852
LOC643072
LOC643201
LOC643339
LOC643406
LOC643441
LOC643486
LOC643542
LOC643623
LOC643677
LOC643711
LOC643719
LOC643733
LOC643837
LOC643923
LOC643955
LOC644145
LOC644165
LOC644172
LOC644538
LOC644554
LOC644649
LOC644669
LOC645323
LOC645332
LOC645513
LOC645638
LOC645676
LOC645752
LOC645961
LOC646214
LOC646268
LOC646405
LOC646498
LOC646626
LOC646627
LOC646736
LOC646762
LOC646851
LOC646982
LOC646999
LOC647121
LOC647323
LOC647946
LOC647979
LOC648691
LOC648740
LOC648987

LOC651250
LOC652276
LOC653486
LOC653602
LOC653653
LOC653712
LOC653786
LOC654841
LOC723809
LOC723972
LOC727924
LOC728024
LOC728040
LOC728175
LOC728190
LOC728264
LOC728276
LOC728392
LOC728407
LOC728554
LOC728606
LOC728613
LOC728643
LOC728661
LOC728723
LOC728730
LOC728743
LOC728752
LOC728855
LOC728875
LOC728989
LOC729080
LOC729156
LOC729159
LOC729234
LOC729348
LOC729375
LOC729384
LOC729467
LOC729506
LOC729603
LOC729609
LOC729658
LOC729668
LOC729678
LOC729950
LOC729970
LOC729991-MEF2B
LOC730183
LOC730668

LOC730755
LOC731779
LOC731789
LOC732275
LOC80054
LOC81691
LOC84740
LOC84856
LOC84931
LOC90110
LOC90246
LOC90586
LOC90768
LOC90784
LOC90834
LOC91149
LOC91316
LOC92249
LOC92659
LOC92973
LOC93432
LOC93622
LOC96610
LOH12CR1
LOH12CR2
LONP1
LONP2
LONRF1
LONRF2
LONRF3
LOR
LOX
LOXHD1
LOXL1
LOXL1-AS1
LOXL2
LOXL3
LOXL4
LPA
LPAL2
LPAR1
LPAR2
LPAR3
LPAR5
LPAR6
LPCAT1
LPCAT2
LPCAT3
LPCAT4
LPGAT1

LPHN1
LPHN2
LPHN3
LPIN1
LPIN2
LPIN3
LPL
LPO
LPP
LPP-AS1
LPPR1
LPPR2
LPPR3
LPPR4
LPPR5
LPXN
LQK1
LRAT
LRBA
LRCH1
LRCH2
LRCH3
LRCH4
LRCOL1
LRDD
LRFN1
LRFN2
LRFN3
LRFN5
LRG1
LRGUK
LRIG1
LRIG2
LRIG3
LRIT1
LRIT2
LRIT3
LRMP
LRP1
LRP1-AS
LRP10
LRP11
LRP12
LRP1B
LRP2
LRP2BP
LRP3
LRP4
LRP5
LRP5L

LRP6
LRP8
LRPAP1
LRPPRC
LRR1
LRRC1
LRRC10
LRRC10B
LRRC14
LRRC14B
LRRC15
LRRC16A
LRRC16B
LRRC17
LRRC18
LRRC2
LRRC2-AS1
LRRC20
LRRC23
LRRC24
LRRC25
LRRC26
LRRC27
LRRC28
LRRC29
LRRC3
LRRC30
LRRC31
LRRC32
LRRC33
LRRC34
LRRC36
LRRC37A3
LRRC37A6P
LRRC37B
LRRC37B2
LRRC37BP1
LRRC38
LRRC39
LRRC3B
LRRC3C
LRRC4
LRRC40
LRRC41
LRRC42
LRRC43
LRRC45
LRRC47
LRRC48
LRRC49

LRRC4B
LRRC4C
LRRC50
LRRC55
LRRC56
LRRC57
LRRC58
LRRC59
LRRC6
LRRC61
LRRC63
LRRC67
LRRC69
LRRC7
LRRC71
LRRC72
LRRC73
LRRC74A
LRRC74B
LRRC75A
LRRC75A-AS1
LRRC8A
LRRC8B
LRRC8C
LRRC8D
LRRC8E
LRRC9
LRRCC1
LRRFIP1
LRRFIP2
LRRIQ1
LRRIQ4
LRRK1
LRRK2
LRRN1
LRRN2
LRRN3
LRRN4
LRRN4CL
LRRTM1
LRRTM2
LRRTM3
LRRTM4
LRSAM1
LRTM1
LRTM2
LRTOMT
LRWD1
LSAMP
LSG1

LSM1
LSM10
LSM11
LSM12
LSM14A
LSM14B
LSM2
LSM3
LSM4
LSM5
LSM6
LSM7
LSP1
LSR
LSS
LST1
LTA
LTA4H
LTB
LTB4R
LTB4R2
LTBP1
LTBP2
LTBP3
LTBP4
LTBR
LTF
LTK
LTN1
LTV1
LUC7L
LUC7L3
LUCAT1
LUM
LURAP1L
LURAP1L-AS1
LUZP1
LUZP2
LUZP4
LUZP6
LVRN
LXN
LY6D
LY6E
LY6G5B
LY6G5C
LY6G6D
LY6G6E
LY6G6F
LY6H

LY6K
LY75
LY75-CD302
LY86
LY86-AS1
LY9
LY96
LYG1
LYG2
LYL1
LYN
LYNX1
LYPD1
LYPD2
LYPD3
LYPD4
LYPD5
LYPD6
LYPD6B
LYPD8
LYPLA1
LYPLA2
LYPLAL1
LYPLAL1-AS1
LYRM1
LYRM2
LYRM4
LYRM5
LYRM7
LYRM9
LYSMD1
LYSMD2
LYSMD4
LYST
LYVE1
LYZL1
LYZL2
LYZL4
LYZL6
LZIC
LZTFL1
LZTR1
LZTS1
LZTS1-AS1
LZTS2
LZTS3
M1AP
M6PR
MAATS1
MAB21L1

MAB21L2
MAB21L3
MACC1
MACF1
MACROD1
MACROD2
MACROD2-IT1
MAD1L1
MAD2L1
MAD2L1BP
MAD2L2
MADCAM1
MADD
MAEA
MAEL
MAF
MAF1
MAFA
MAFB
MAFF
MAFG
MAFK
MAG
MAGEA1
MAGEA10
MAGEA4
MAGEA8
MAGEB1
MAGEB10
MAGEB16
MAGEB17
MAGEB2
MAGEB3
MAGEB6
MAGEC1
MAGEC3
MAGED1
MAGED2
MAGEE1
MAGEF1
MAGEH1
MAGEL2
MAGI1
MAGI1-AS1
MAGI2
MAGI2-AS3
MAGI3
MAGIX
Magmas
MAGOH

MAGOHB
MAGT1
MAK
MAK16
MAL
MAL2
MALAT1
MALL
MALRD1
MALSU1
MALT1
MAMDC2
MAMDC2-AS1
MAMDC4
MAML1
MAML2
MAML3
MAMLD1
MAMSTR
MAN1A1
MAN1A2
MAN1B1
MAN1C1
MAN2A1
MAN2A2
MAN2B1
MAN2B2
MAN2C1
MANBA
MANBAL
MANEAL
MANF
MANSC1
MANSC4
MAOA
MAOB
MAP1A
MAP1B
MAP1D
MAP1LC3A
MAP1LC3B
MAP1LC3B2
MAP1LC3C
MAP1S
MAP2
MAP2K1
MAP2K2
MAP2K3
MAP2K4
MAP2K5

MAP2K6
MAP2K7
MAP3K1
MAP3K10
MAP3K11
MAP3K12
MAP3K13
MAP3K14
MAP3K14-AS1
MAP3K15
MAP3K19
MAP3K2
MAP3K3
MAP3K4
MAP3K5
MAP3K6
MAP3K7
MAP3K7CL
MAP3K7IP1
MAP3K7IP2
MAP3K7IP3
MAP3K8
MAP3K9
MAP4
MAP4K1
MAP4K2
MAP4K3
MAP4K4
MAP4K5
MAP6
MAP6D1
MAP7
MAP7D1
MAP7D2
MAP7D3
MAP9
MAPK1
MAPK10
MAPK11
MAPK12
MAPK13
MAPK14
MAPK15
MAPK1IP1L
MAPK3
MAPK4
MAPK6
MAPK7
MAPK8
MAPK8IP1

MAPK8IP2
MAPK8IP3
MAPK9
MAPKAP1
MAPKAPK2
MAPKAPK3
MAPKAPK5
MAPKAPK5-AS1
MAPKBP1
MAPRE1
MAPRE2
MAPRE3
MAPT
MAPT-AS1
1-Mar
2-Mar
1-Mar
10-Mar
11-Mar
2-Mar
3-Mar
4-Mar
5-Mar
6-Mar
7-Mar
8-Mar
9-Mar
MARCKS
MARCKSL1
MARCO
MARK1
MARK2
MARK3
MARK4
MARS
MARS2
MARVELD2
MARVELD3
MAS1L
MASP1
MASP2
MAST1
MAST2
MAST3
MAST4
MASTL
MAT1A
MAT2A
MAT2B
MATK

MATN1
MATN2
MATN3
MATN4
MATR3
MAU2
MAVS
MAX
MAZ
MB
MB21D2
MBD1
MBD2
MBD3
MBD4
MBD5
MBD6
MBIP
MBL2
MBLAC1
MBLAC2
MBNL1
MBNL1-AS1
MBNL2
MBNL3
MBOAT1
MBOAT2
MBOAT7
MBP
MBTD1
MBTPS1
MC1R
MC2R
MC3R
MC4R
MC5R
MCAM
MCART1
MCART2
MCART6
MCAT
MCC
MCCC1
MCCC2
MCCD1
MCEE
MCEMP1
MCF2
MCF2L
MCF2L2

MCFD2
MCHR1
MCHR2
MCHR2-AS1
MCIDAS
MCL1
MCM10
MCM2
MCM3
MCM3AP
MCM3AP-AS1
MCM4
MCM5
MCM6
MCM7
MCM8
MCM9
MCMBP
MCOLN1
MCOLN2
MCOLN3
MCPH1
MCPH1-AS1
MCRS1
MCTP1
MCTP2
MCTS1
MCU
MCUR1
MDC1
MDFI
MDFIC
MDGA1
MDGA2
MDH1
MDH1B
MDH2
MDK
MDM1
MDM2
MDM4
MDN1
MDP1
MDS2
ME1
ME2
ME3
MEA1
MEAF6
MECOM

MECP2
MECR
MED1
MED10
MED11
MED12
MED12L
MED13
MED13L
MED14
MED15
MED15P9
MED16
MED17
MED18
MED19
MED20
MED21
MED22
MED23
MED24
MED25
MED26
MED27
MED28
MED29
MED30
MED31
MED6
MED7
MED8
MED9
MEDAG
MEF2A
MEF2B
MEF2BNB
MEF2C
MEF2C-AS1
MEF2D
MEFV
MEG3
MEG8
MEG9
MEGF10
MEGF11
MEGF6
MEGF8
MEGF9
MEI1
MEI4

MEIG1
MEIKIN
MEIS1
MEIS1-AS2
MEIS1-AS3
MEIS2
MEIS3
MEIS3P1
MELK
MEMO1
MEN1
MEOX1
MEOX2
MEP1A
MEP1B
MEPCE
MEPE
MERTK
MESDC1
MESDC2
MESP1
MESP2
MEST
MESTIT1
MET
METAP1
METAP1D
METAP2
METRN
METRNL
METT10D
METT11D1
METT5D1
METTL1
METTL10
METTL11B
METTL13
METTL14
METTL15
METTL16
METTL17
METTL20
METTL21A
METTL21B
METTL21C
METTL22
METTL25
METTL2A
METTL2B
METTL3

METTL4
METTL5
METTL6
METTL7A
METTL7B
METTL8
METTL9
MEX3A
MEX3B
MEX3C
MEX3D
MFAP1
MFAP2
MFAP3
MFAP3L
MFAP4
MFAP5
MFF
MFGE8
MFHAS1
MFI2
MFN1
MFN2
MFNG
MFSD1
MFSD10
MFSD11
MFSD2A
MFSD2B
MFSD3
MFSD4
MFSD5
MFSD6
MFSD6L
MFSD7
MFSD8
MFSD9
MGA
MGAM
MGAM2
MGARP
MGAT1
MGAT3
MGAT4A
MGAT4B
MGAT4C
MGAT4D
MGAT4EP
MGAT5
MGAT5B

MGC12982
MGC13005
MGC14436
MGC15885
MGC16121
MGC16275
MGC23270
MGC23284
MGC26597
MGC27345
MGC27382
MGC2752
MGC2889
MGC29506
MGC34034
MGC42105
MGC4473
MGC45800
MGC57346
MGC57346-CRHR1
MGC70857
MGC72080
MGC87042
MGEA5
MGLL
MGMT
MGP
MGRN1
MGST1
MGST2
MGST3
MIA
MIA3
MIAT
MIATNB
MIB1
MIB2
MICA
MICAL1
MICAL2
MICAL3
MICALCL
MICALL1
MICALL2
MICB
MICU1
MICU2
MICU3
MID1
MID1IP1

MID2
MIDN
MIEF1
MIEF2
MIER1
MIER2
MIER3
MIF
MIF-AS1
MIF4GD
MIIP
MILR1
MIMT1
MINA
MINK1
MINPP1
MIOS
MIOX
MIP
MIPEP
MIPEPP3
MIPOL1
MIR1-1
MIR1-1HG
MIR1-2
MIR100HG
MIR101-1
MIR10A
MIR10B
MIR1178
MIR1180
MIR1182
MIR1183
MIR1185-1
MIR1185-2
MIR1193
MIR1200
MIR1203
MIR1204
MIR1205
MIR1207
MIR122
MIR1228
MIR1229
MIR1237
MIR124-1
MIR124-2
MIR124-2HG
MIR124-3
MIR1246

MIR1248
MIR1253
MIR1254-1
MIR1255A
MIR1256
MIR1258
MIR125A
MIR125B1
MIR126
MIR1260
MIR1261
MIR1266
MIR1267
MIR1268A
MIR1269A
MIR1269B
MIR127
MIR1273A
MIR1273E
MIR1273H
MIR1276
MIR128-2
MIR1284
MIR1286
MIR1288
MIR129-2
MIR1295
MIR1297
MIR1301
MIR1302-1
MIR1302-6
MIR1302-8
MIR1306
MIR1307
MIR130B
MIR132
MIR1322
MIR1323
MIR133A1HG
MIR133B
MIR134
MIR135A2
MIR135B
MIR136
MIR137
MIR143HG
MIR145
MIR1468
MIR146A
MIR147

MIR1470
MIR152
MIR153-2
MIR1539
MIR154
MIR155
MIR155HG
MIR15B
MIR16-1
MIR17HG
MIR181A1
MIR181A1HG
MIR181A2HG
MIR181D
MIR182
MIR1826
MIR1827
MIR183
MIR186
MIR187
MIR1908
MIR190B
MIR191
MIR1913
MIR193A
MIR194-2
MIR195
MIR196A1
MIR196A2
MIR196B
MIR197
MIR1974
MIR1977
MIR199A1
MIR199A2
MIR199B
MIR19A
MIR200A
MIR200B
MIR202
MIR203A
MIR2052
MIR2052HG
MIR206
MIR208B
MIR20B
MIR21
MIR210
MIR2110
MIR2113

MIR2114
MIR2116
MIR2117
MIR212
MIR216B
MIR217HG
MIR218-1
MIR218-2
MIR219-2
MIR219A2
MIR223
MIR224
MIR22HG
MIR23A
MIR24-1
MIR24-2
MIR2681
MIR296
MIR299
MIR29A
MIR29B2
MIR301B
MIR302A
MIR3065
MIR30A
MIR30B
MIR30C1
MIR30C2
MIR3115
MIR3122
MIR3132
MIR3134
MIR3145
MIR3166
MIR3174
MIR3175
MIR3179-4
MIR3197
MIR31HG
MIR320B1
MIR320C1
MIR325HG
MIR329-2
MIR330
MIR335
MIR337
MIR33B
MIR345
MIR346
MIR34A

MIR34B
MIR361
MIR3611
MIR3646
MIR365-2
MIR3663
MIR3663HG
MIR3666
MIR3679
MIR3680-2
MIR369
MIR370
MIR375
MIR376B
MIR377
MIR378A
MIR379
MIR380
MIR381
MIR382
MIR384
MIR3909
MIR3929
MIR3945
MIR3960
MIR3972
MIR3976HG
MIR409
MIR410
MIR411
MIR412
MIR425
MIR4254
MIR4293
MIR4299
MIR431
MIR4319
MIR432
MIR4324
MIR4327
MIR4421
MIR4423
MIR4435-2HG
MIR4442
MIR449A
MIR4505
MIR450A1
MIR451
MIR4519
MIR4524A

MIR4524B
MIR453
MIR454
MIR4636
MIR4643
MIR4678
MIR4679-2
MIR4681
MIR4688
MIR4692
MIR4706
MIR4708
MIR4722
MIR4738
MIR4740
MIR4757
MIR4769
MIR4774
MIR4777
MIR4785
MIR4787
MIR4792
MIR4799
MIR483
MIR485
MIR486-1
MIR487A
MIR487B
MIR488
MIR489
MIR490
MIR492
MIR493
MIR494
MIR495
MIR496
MIR498
MIR499
MIR5001
MIR5003
MIR5006
MIR5009
MIR504
MIR508
MIR509-2
MIR5095
MIR5096
MIR512-1
MIR513A1
MIR513A2

MIR514-2
MIR514B
MIR515-2
MIR516A1
MIR518B
MIR518D
MIR518F
MIR519B
MIR520E
MIR521-1
MIR524
MIR525
MIR526A1
MIR541
MIR542
MIR543
MIR548A2
MIR548AC
MIR548AE2
MIR548AI
MIR548AJ2
MIR548AL
MIR548AO
MIR548AP
MIR548AU
MIR548AY
MIR548AZ
MIR548D2
MIR548F1
MIR548F3
MIR548F5
MIR548G
MIR548H1
MIR548H2
MIR548H3
MIR548H4
MIR548I2
MIR548I4
MIR548L
MIR548N
MIR548O2
MIR548Q
MIR548W
MIR549
MIR550-2
MIR551B
MIR553
MIR556
MIR557
MIR558

MIR5588
MIR566
MIR568
MIR5694
MIR570
MIR5703
MIR5707
MIR572
MIR573
MIR574
MIR577
MIR578
MIR5787
MIR583
MIR585
MIR588
MIR589
MIR592
MIR596
MIR598
MIR600
MIR602
MIR603
MIR607
MIR611
MIR612
MIR613
MIR6130
MIR6132
MIR614
MIR620
MIR629
MIR633
MIR636
MIR638
MIR639
MIR641
MIR642
MIR645
MIR646
MIR646HG
MIR651
MIR6511B2
MIR653
MIR654
MIR655
MIR656
MIR657
MIR661
MIR663

MIR665
MIR670
MIR670HG
MIR671
MIR6715A
MIR6728
MIR6729
MIR6733
MIR6737
MIR6740
MIR6742
MIR6781
MIR6784
MIR6790
MIR6791
MIR6796
MIR6819
MIR6830
MIR6850
MIR6854
MIR6863
MIR6880
MIR7-2
MIR711
MIR7152
MIR744
MIR7515
MIR7515HG
MIR758
MIR759
MIR760
MIR7641-2
MIR769
MIR770
MIR7847
MIR7853
MIR8061
MIR8065
MIR8074
MIR8085
MIR8485
MIR889
MIR891A
MIR892A
MIR9-1
MIR9-2
MIR9-3
MIR922
MIR936
MIR937

MIR941-1
MIR942
MIR944
MIR95
MIR96
MIR98
MIR99AHG
MIRLET7A1
MIRLET7A3
MIRLET7BHG
MIRLET7DHG
MIRLET7F1
MIRLET7I
MIS12
MIS18A
MIS18BP1
MISP
MITD1
MITF
MIXL1
MKI67IP
MKKS
MKL1
MKL2
MKLN1
MKNK1
MKNK1-AS1
MKNK2
MKRN1
MKRN2
MKRN2OS
MKRN3
MKS1
MKX
MLANA
MLC1
MLEC
MLF1
MLF1IP
MLF2
MLH1
MLH3
MLIP
MLIP-IT1
MLK7-AS1
MLKL
MLL
MLL2
MLL3
MLL5

MLLT1
MLLT10
MLLT11
MLLT3
MLLT4
MLLT4-AS1
MLLT6
MLN
MLNR
MLPH
MLST8
MLX
MLXIP
MLXIPL
MLYCD
MMAA
MMAB
MMACHC
MMADHC
MMD
MMD2
MME
MMEL1
MMGT1
MMP1
MMP10
MMP11
MMP12
MMP13
MMP14
MMP15
MMP16
MMP17
MMP19
MMP2
MMP20
MMP21
MMP23A
MMP24
MMP24-AS1
MMP25
MMP25-AS1
MMP26
MMP27
MMP28
MMP3
MMP7
MMP9
MMRN1
MMRN2

MMS19
MN1
MNAT1
MND1
MNDA
MNS1
MNT
MNX1
MOB1B
MOB2
MOB3B
MOB3C
MOBKL1A
MOBKL2A
MOBKL2B
MOBKL2C
MOBKL3
MOBP
MOCOS
MOCS1
MOCS2
MOG
MOGAT1
MOGAT2
MOGAT3
MOGS
MOK
MON1A
MON1B
MON2
MORC1
MORC2
MORC3
MORC4
MORF4L1
MORF4L2
MORG1
MORN1
MORN2
MORN3
MORN4
MORN5
MOS
MOSC1
MOSC2
MOSPD1
MOSPD2
MOSPD3
MOV10
MOV10L1

MOXD1
MOXD2
MOXD2P
MPDU1
MPDZ
MPEG1
MPG
MPHOSPH10
MPHOSPH6
MPHOSPH8
MPHOSPH9
MPI
MPL
MPND
MPO
MPP1
MPP2
MPP3
MPP4
MPP5
MPP6
MPP7
MPPE1
MPPED1
MPPED2
MPRIP
MPST
MPV17
MPV17L
MPV17L2
MPZL1
MPZL3
MR1
MRAP
MRAP2
MRAS
MRC1
MRC2
MRE11A
MREG
MRFAP1
MRGPRE
MRGPRF
MRGPRG
MRGPRG-AS1
MRGPRX1
MRGPRX3
MRI1
MRM1
MRO

MROH1
MROH2A
MROH2B
MROH5
MROH6
MROH7
MROH7-TTC4
MROH8
MROH9
MRP63
MRPL1
MRPL10
MRPL11
MRPL12
MRPL13
MRPL15
MRPL16
MRPL17
MRPL18
MRPL19
MRPL20
MRPL21
MRPL22
MRPL23
MRPL23-AS1
MRPL24
MRPL28
MRPL3
MRPL30
MRPL32
MRPL33
MRPL35
MRPL37
MRPL38
MRPL39
MRPL4
MRPL42
MRPL42P5
MRPL43
MRPL44
MRPL45
MRPL46
MRPL48
MRPL49
MRPL50
MRPL51
MRPL52
MRPL54
MRPL55
MRPS10

MRPS11
MRPS12
MRPS15
MRPS16
MRPS17
MRPS18A
MRPS18B
MRPS18C
MRPS21
MRPS22
MRPS23
MRPS25
MRPS26
MRPS27
MRPS28
MRPS30
MRPS31
MRPS31P5
MRPS33
MRPS34
MRPS35
MRPS36
MRPS5
MRPS6
MRPS7
MRPS9
MRRF
MRS2
MRTO4
MRVI1
MRVI1-AS1
MS4A1
MS4A10
MS4A12
MS4A13
MS4A14
MS4A15
MS4A18
MS4A2
MS4A3
MS4A4A
MS4A5
MS4A6A
MS4A6E
MS4A7
MS4A8
MS4A8B
MSANTD1
MSANTD2
MSANTD3

MSANTD4
MSC
MSC-AS1
MSH2
MSH3
MSH4
MSH5
MSH5-SAPCD1
MSH6
MSI1
MSI2
MSL1
MSL2
MSL3
MSLN
MSLNL
MSMB
MSN
MSR1
MSRA
MSRB2
MSRB3
MST1
MST1L
MST1P2
MST1P9
MST1R
MST4
MSTO1
MSX1
MSX2
MSX2P1
MT1A
MT1DP
MT1F
MT1G
MT1HL1
MT1IP
MT1JP
MT1M
MT1X
MT2A
MT3
MT4
MTA1
MTA2
MTA3
MTAP
MTBP
MTCH1

MTCH2
MTCL1
MTCP1NB
MTDH
MTERF
MTERF1
MTERF3
MTERFD1
MTERFD2
MTERFD3
MTF1
MTF2
MTFMT
MTFR1
MTFR1L
MTFR2
MTG1
MTG2
MTHFD1
MTHFD1L
MTHFD2
MTHFD2L
MTHFD2P1
MTHFR
MTHFS
MTHFSD
MTIF2
MTIF3
MTL5
MTM1
MTMR1
MTMR10
MTMR11
MTMR12
MTMR14
MTMR15
MTMR2
MTMR3
MTMR4
MTMR6
MTMR7
MTMR8
MTMR9
MTMR9L
MTMR9LP
MTNR1A
MTNR1B
MTO1
MTOR
MTOR-AS1

MTP18
MTPAP
MTPN
MTR
MTRF1
MTRNR2L1
MTRNR2L10
MTRNR2L4
MTRNR2L5
MTRNR2L7
MTRR
MTSS1
MTSS1L
MTTP
MTURN
MTUS1
MTUS2
MTX1
MTX2
MTX3
MUC1
MUC12
MUC13
MUC15
MUC16
MUC17
MUC19
MUC2
MUC20
MUC21
MUC22
MUC3A
MUC4
MUC5B
MUC6
MUC7
MUCL1
MUDENG
MUL1
MUM1
MUM1L1
MUPCDH
MURC
MUS81
MUSK
MUSTN1
MUTYH
MVB12A
MVB12B
MVD

MVK
MVP
MX1
MX2
MXD1
MXD3
MXD4
MXI1
MXRA5
MXRA7
MXRA8
MYADM
MYADML2
MYB
MYBBP1A
MYBL1
MYBL2
MYBPC1
MYBPC2
MYBPC3
MYBPH
MYBPHL
MYC
MYCBP
MYCBP2
MYCBPAP
MYCL
MYCL1
MYCN
MYCNUT
MYD88
MYEF2
MYEOV
MYEOV2
MYF6
MYH1
MYH10
MYH11
MYH13
MYH14
MYH15
MYH16
MYH2
MYH3
MYH4
MYH6
MYH7
MYH7B
MYH9
MYHAS

MYL1
MYL10
MYL12A
MYL12B
MYL2
MYL3
MYL4
MYL5
MYL6
MYL6B
MYL7
MYL9
MYLIP
MYLK
MYLK-AS1
MYLK-AS2
MYLK2
MYLK3
MYLK4
MYLPF
MYNN
MYO10
MYO15A
MYO15B
MYO16
MYO18A
MYO18B
MYO19
MYO1A
MYO1B
MYO1C
MYO1D
MYO1E
MYO1F
MYO1G
MYO1H
MYO3A
MYO3B
MYO5A
MYO5B
MYO5C
MYO6
MYO7A
MYO7B
MYO9A
MYO9B
MYOCD
MYOD1
MYOF
MYOG

MYOM1
MYOM2
MYOM3
MYOT
MYOZ1
MYOZ2
MYOZ3
MYPN
MYPOP
MYRF
MYRFL
MYRIP
MYSM1
MYST1
MYST2
MYST3
MYST4
MYT1
MYT1L
MZF1
N4BP1
N4BP2
N4BP2L1
N4BP2L2
N4BP3
N6AMT1
N6AMT2
NAA10
NAA11
NAA15
NAA16
NAA20
NAA25
NAA30
NAA35
NAA38
NAA40
NAA50
NAA60
NAALAD2
NAALADL1
NAALADL2
NAALADL2-AS1
NAALADL2-AS2
NAALADL2-AS3
NAB1
NAB2
NABP1
NABP2
NACA

NACA2
NACAD
NACAP1
NACC1
NACC2
NADK
NADK2
NADSYN1
NAE1
NAF1
NAGA
NAGK
NAGLU
NAGPA
NAGPA-AS1
NAGS
NAIF1
NAIP
NALCN
NALCN-AS1
NAMA
NAMPT
NANOG
NANOS1
NANOS2
NANOS3
NANP
NANS
NAP1L1
NAP1L2
NAP1L4
NAP1L5
NAP1L6
NAPA
NAPB
NAPEPLD
NAPG
NAPSA
NAPSB
NARF
NARFL
NARG2
NARR
NARS
NARS2
NASP
NAT1
NAT10
NAT14
NAT15

NAT16
NAT2
NAT6
NAT8
NAT8B
NAT8L
NATD1
NAV1
NAV2
NAV2-AS4
NAV2-AS5
NAV3
NBAS
NBAT1
NBEA
NBEAL1
NBEAL2
NBEAP1
NBL1
NBLA00301
NBN
NBPF1
NBPF10
NBPF15
NBPF20
NBPF25P
NBPF3
NBPF4
NBPF8
NBR1
NBR2
NCALD
NCAM1
NCAM2
NCAN
NCAPD2
NCAPD3
NCAPG2
NCAPH
NCAPH2
NCBP2
NCCRP1
NCDN
NCEH1
NCF2
NCF4
NCK1
NCK2
NCKAP1
NCKAP1L

NCKAP5
NCKAP5L
NCKIPSD
NCL
NCLN
NCMAP
NCOA1
NCOA2
NCOA3
NCOA4
NCOA5
NCOA6
NCOA7
NCOR1
NCOR2
NCR1
NCR2
NCR3LG1
NCRNA00028
NCRNA00029
NCRNA00032
NCRNA00051
NCRNA00086
NCRNA00087
NCRNA00092
NCRNA00093
NCRNA00095
NCRNA00111
NCRNA00114
NCRNA00116
NCRNA00119
NCRNA00162
NCRNA00164
NCRNA00167
NCRNA00171
NCRNA00174
NCRNA00175
NCRNA00176
NCRNA00181
NCRNA00182
NCRNA00188
NCRNA00200
NCRNA00202
NCRNA00207
NCRNA00219
NCRNA00250
NCS1
NCSTN
NDC1
NDC80

NDE1
NDEL1
NDFIP1
NDFIP2
NDNF
NDNL2
NDOR1
NDP
NDRG1
NDRG2
NDRG3
NDRG4
NDST1
NDST2
NDST3
NDST4
NDUFA10
NDUFA11
NDUFA12
NDUFA13
NDUFA2
NDUFA3
NDUFA4
NDUFA4L2
NDUFA5
NDUFA6
NDUFA6-AS1
NDUFA7
NDUFA8
NDUFA9
NDUFAB1
NDUFAF1
NDUFAF2
NDUFAF3
NDUFAF4
NDUFAF4P1
NDUFAF5
NDUFAF6
NDUFAF7
NDUFB10
NDUFB11
NDUFB2
NDUFB3
NDUFB4
NDUFB5
NDUFB6
NDUFB7
NDUFB8
NDUFB9
NDUFC1

NDUFC2
NDUFC2-KCTD14
NDUFS1
NDUFS2
NDUFS4
NDUFS5
NDUFS6
NDUFS7
NDUFS8
NDUFV1
NDUFV2
NDUFV3
NEAT1
NEB
NEBL
NEBL-AS1
NECAB1
NECAB2
NECAB3
NECAP1
NECAP2
NEDD1
NEDD4
NEDD4L
NEDD8
NEDD9
NEFH
NEFL
NEFM
NEGR1
NEGR1-IT1
NEIL1
NEIL2
NEIL3
NEK1
NEK10
NEK11
NEK3
NEK4
NEK5
NEK6
NEK7
NEK9
NELF
NELFA
NELFCD
NELL1
NELL2
NEMF
NEMP1

NEMP2
NENF
NEO1
NES
NET1
NETO1
NETO2
NEU1
NEU2
NEU3
NEU4
NEURL
NEURL1
NEURL1-AS1
NEURL1B
NEURL3
NEURL4
NEUROD1
NEUROD2
NEUROD4
NEUROD6
NEUROG1
NEUROG2
NEUROG3
NEXN
NEXN-AS1
NF1
NF2
NFAM1
NFASC
NFAT5
NFATC1
NFATC2
NFATC2IP
NFATC3
NFATC4
NFE2
NFE2L1
NFE2L2
NFE2L3
NFIA
NFIA-AS1
NFIA-AS2
NFIB
NFIC
NFIL3
NFIX
NFKB1
NFKB2
NFKBIA

NFKBIB
NFKBID
NFKBIL1
NFKBIL2
NFKBIZ
NFRKB
NFS1
NFU1
NFX1
NFXL1
NFYA
NFYB
NFYC
NFYC-AS1
NGB
NGDN
NGEF
NGF
NGFR
NGFRAP1
NGLY1
NGRN
NHEDC1
NHEG1
NHEJ1
NHLH1
NHLH2
NHLRC1
NHLRC2
NHLRC3
NHLRC4
NHP2
NHP2L1
NHS
NHS-AS1
NHSL1
NHSL2
NICN1
NID1
NID2
NIF3L1
NIFK-AS1
NIM1K
NIN
NINJ1
NINJ2
NINL
NIP7
NIPA1
NIPAL2

NIPAL3
NIPAL4
NIPBL
NIPBL-AS1
NIPSNAP1
NIPSNAP3A
NIPSNAP3B
NISCH
NIT1
NIT2
NKAIN1
NKAIN2
NKAIN3
NKAIN4
NKAP
NKAPL
NKAPP1
NKD1
NKD2
NKG7
NKIRAS1
NKIRAS2
NKPD1
NKRF
NKTR
NKX1-1
NKX1-2
NKX2-1
NKX2-1-AS1
NKX2-2
NKX2-3
NKX2-4
NKX2-5
NKX2-6
NKX2-8
NKX3-1
NKX3-2
NKX6-1
NKX6-2
NKX6-3
NLE1
NLGN1
NLGN2
NLGN3
NLGN4X
NLGN4Y
NLK
NLN
NLRC3
NLRC5

NLRP1
NLRP10
NLRP11
NLRP12
NLRP14
NLRP2
NLRP3
NLRP4
NLRP5
NLRP6
NLRP7
NLRP8
NLRP9
NLRX1
NMB
NMBR
NMD3
NME1
NME2
NME3
NME4
NME5
NME7
NME8
NME9
NMI
NMNAT1
NMNAT2
NMNAT3
NMRAL1
NMRK1
NMRK2
NMS
NMT1
NMT2
NMU
NMUR1
NMUR2
NNMT
NNT
NOB1
NOBOX
NOC2L
NOC3L
NOC4L
NOCT
NOD1
NOD2
NODAL
NOG

NOL10
NOL11
NOL12
NOL3
NOL4
NOL4L
NOL6
NOL7
NOL8
NOL9
NOLC1
NOM1
NOMO1
NOMO3
NONO
NOP10
NOP14
NOP16
NOP2
NOP56
NOP58
NOP9
NOS1
NOS1AP
NOS2
NOS3
NOSIP
NOSTRIN
NOTCH1
NOTCH2
NOTCH2NL
NOTCH3
NOTCH4
NOTO
NOTUM
NOV
NOVA1
NOVA2
NOX1
NOX3
NOX4
NOX5
NOXA1
NOXO1
NPAP1
NPAS1
NPAS2
NPAS3
NPAS4
NPAT

NPB
NPBWR1
NPBWR2
NPC1
NPC1L1
NPC2
NPDC1
NPEPL1
NPEPPS
NPFF
NPFFR1
NPFFR2
NPHP1
NPHP3
NPHP3-AS1
NPHP4
NPHS1
NPHS2
NPIPB8
NPIPB9
NPL
NPLOC4
NPM1
NPM2
NPM3
NPNT
NPPA
NPPA-AS1
NPPB
NPPC
NPR1
NPR2
NPR3
NPRL3
NPS
NPSR1
NPSR1-AS1
NPTN
NPTX1
NPTX2
NPTXR
NPVF
NPW
NPY
NPY1R
NPY2R
NPY5R
NPY6R
NQO1
NQO2

NR0B1
NR0B2
NR1D1
NR1D2
NR1H3
NR1H4
NR1I2
NR1I3
NR2C1
NR2C2
NR2C2AP
NR2E1
NR2E3
NR2F1
NR2F1-AS1
NR2F2
NR2F2-AS1
NR2F6
NR3C1
NR3C2
NR4A1
NR4A2
NR4A3
NR5A1
NR5A2
NR6A1
NRAP
NRARP
NRAS
NRBF2
NRBP1
NRBP2
NRCAM
NRD1
NRDE2
NREP
NREP-AS1
NRF1
NRG1
NRG2
NRG3
NRG3-AS1
NRG4
NRGN
NRIP1
NRIP2
NRIP3
NRIR
NRL
NRM

NRN1
NRN1L
NRON
NRP1
NRP2
NRROS
NRSN1
NRSN2
NRTN
NRXN1
NRXN2
NRXN3
NSA2
NSD1
NSDHL
NSF
NSFL1C
NSG1
NSMAF
NSMCE1
NSMCE2
NSMCE4A
NSUN2
NSUN3
NSUN4
NSUN5
NSUN5B
NSUN6
NSUN7
NT5C
NT5C1A
NT5C1B
NT5C1B-RDH14
NT5C2
NT5C3
NT5C3A
NT5C3L
NT5DC1
NT5DC2
NT5DC3
NT5E
NT5M
NTAN1
NTF3
NTHL1
NTM
NTM-IT
NTN1
NTN4
NTN5

NTNG1
NTNG2
NTPCR
NTRK1
NTRK2
NTRK3
NTRK3-AS1
NTSR1
NTSR2
NUAK1
NUAK2
NUB1
NUBP1
NUBP2
NUBPL
NUCB1
NUCB1-AS1
NUCKS1
NUDC
NUDCD1
NUDCD2
NUDCD3
NUDT1
NUDT10
NUDT12
NUDT13
NUDT14
NUDT15
NUDT16
NUDT16L1
NUDT16P
NUDT17
NUDT18
NUDT2
NUDT22
NUDT3
NUDT4
NUDT4P2
NUDT5
NUDT6
NUDT7
NUDT8
NUDT9
NUF2
NUFIP1
NUFIP2
NUGGC
NUMA1
NUMB
NUMBL

NUP107
NUP133
NUP153
NUP155
NUP160
NUP205
NUP210
NUP210L
NUP210P1
NUP214
NUP35
NUP37
NUP43
NUP50
NUP50-AS1
NUP54
NUP62
NUP62CL
NUP85
NUP88
NUP93
NUP98
NUPL1
NUPL2
NUPR1
NUS1
NUSAP1
NUTF2
NUTM2A-AS1
NUTM2F
NVL
NWD1
NWD2
NXF1
NXF2
NXF3
NXF5
NXN
NXNL1
NXNL2
NXPE1
NXPE2
NXPE3
NXPE4
NXPH1
NXPH2
NXPH3
NXPH4
NXT1
NXT2

NYAP1
NYAP2
NYNRIN
NYX
OACYLP
OAF
OARD1
OAS1
OAS2
OAS3
OASL
OAT
OAZ1
OAZ2
OAZ3
OBFC1
OBFC2A
OBFC2B
OBP2A
OBP2B
OBSCN
OBSL1
OC90
OCA2
OCEL1
OCIAD1
OCIAD2
OCLN
OCM
OCM2
OCRL
OCSTAMP
ODAM
ODC1
ODF1
ODF2
ODF2L
ODF3
ODF3L1
ODF3L2
ODF4
ODZ1
ODZ2
ODZ3
ODZ4
OFD1
OGDH
OGDHL
OGFOD1
OGFOD2

OGFOD3
OGFR
OGFR-AS1
OGFRL1
OGFRP1
OGG1
OGT
OIP5
OIP5-AS1
OIT3
OLA1
OLAH
OLFM1
OLFM2
OLFM3
OLFM4
OLFML1
OLFML2A
OLFML2B
OLFML3
OLIG1
OLIG2
OLIG3
OLR1
OMA1
OMP
ONECUT1
ONECUT2
ONECUT3
OOEP
OOSP1
OOSP2
OPA1
OPA3
OPALIN
OPCML
OPHN1
OPLAH
OPN1LW
OPN1SW
OPN3
OPN4
OPN5
OPRD1
OPRK1
OPRL1
OPRM1
OPTC
OPTN
OR10A2

OR10A3
OR10AD1
OR10AG1
OR10C1
OR10G2
OR10G4
OR10G8
OR10H1
OR10H2
OR10H5
OR10J1
OR10J3
OR10J5
OR10K1
OR10K2
OR10Q1
OR10S1
OR10T2
OR10V1
OR10W1
OR10X1
OR10Z1
OR11A1
OR11G2
OR11H12
OR11H6
OR11L1
OR12D2
OR12D3
OR13A1
OR13C2
OR13C3
OR13C9
OR13D1
OR13J1
OR14J1
OR1A1
OR1A2
OR1B1
OR1C1
OR1E2
OR1F1
OR1F2P
OR1G1
OR1I1
OR1J1
OR1J4
OR1L1
OR1L3
OR1N1

OR1N2
OR1S2
OR2A2
OR2A5
OR2AG1
OR2AP1
OR2B11
OR2C1
OR2C3
OR2F1
OR2F2
OR2G3
OR2G6
OR2H1
OR2H2
OR2J3
OR2L13
OR2L3
OR2L5
OR2L8
OR2M1P
OR2M2
OR2M3
OR2M4
OR2M5
OR2M7
OR2T10
OR2T27
OR2T33
OR2T35
OR2T4
OR2T6
OR2T8
OR2V1
OR2W1
OR2W5
OR2Y1
OR3A1
OR3A2
OR3A4
OR4A15
OR4A16
OR4A47
OR4B1
OR4C11
OR4C12
OR4C15
OR4C3
OR4D1
OR4D10

OR4D11
OR4D2
OR4D5
OR4D6
OR4D9
OR4F13P
OR4F6
OR4K17
OR4L1
OR4M1
OR4M2
OR4N2
OR4S1
OR4S2
OR51A2
OR51A4
OR51B2
OR51B4
OR51B5
OR51B6
OR51D1
OR51E1
OR51E2
OR51F2
OR51G1
OR51G2
OR51I1
OR51L1
OR51Q1
OR51S1
OR51T1
OR51V1
OR52A4
OR52B2
OR52B4
OR52E8
OR52I1
OR52I2
OR52K1
OR52K2
OR52M1
OR52N1
OR52W1
OR56A1
OR56A3
OR56A4
OR56A5
OR5A2
OR5AN1
OR5AS1

OR5AU1
OR5B12
OR5B3
OR5C1
OR5D13
OR5D14
OR5D16
OR5E1P
OR5F1
OR5H1
OR5H14
OR5H2
OR5H6
OR5I1
OR5K2
OR5L2
OR5M1
OR5M10
OR5M8
OR5P3
OR5R1
OR5T1
OR5T3
OR6A2
OR6B1
OR6B2
OR6B3
OR6C3
OR6C4
OR6C68
OR6C70
OR6C75
OR6C76
OR6F1
OR6K2
OR6K3
OR6K6
OR6N1
OR6P1
OR6S1
OR6T1
OR6V1
OR6W1P
OR6X1
OR6Y1
OR7A17
OR7D2
OR7D4
OR7E24
OR7E37P

OR7E47P
OR7G1
OR7G2
OR8A1
OR8B12
OR8B2
OR8B3
OR8B4
OR8B8
OR8D1
OR8D2
OR8D4
OR8G1
OR8G5
OR8H1
OR8H2
OR8J3
OR8K3
OR8K5
OR8S1
OR8U8
OR9A2
OR9A4
OR9G4
OR9K2
OR9Q1
OR9Q2
ORAI1
ORAI2
ORAI3
ORAOV1
ORC1
ORC1L
ORC2
ORC2L
ORC3
ORC3L
ORC5L
ORC6L
ORM1
ORMDL3
OS9
OSBP
OSBP2
OSBPL10
OSBPL10-AS1
OSBPL11
OSBPL1A
OSBPL2
OSBPL3

OSBPL5
OSBPL6
OSBPL8
OSBPL9
OSCAR
OSCP1
OSER1
OSER1-AS1
OSGEP
OSGEPL1
OSGEPL1-AS1
OSGIN1
OSGIN2
OSMR
OSMR-AS1
OSR1
OSR2
OST4
OSTalpha
OSTBETA
OSTC
OSTCP1
OSTF1
OSTM1
OSTN
OTC
OTOA
OTOF
OTOG
OTOGL
OTOL1
OTOP1
OTOP2
OTOP3
OTOR
OTOS
OTP
OTUB1
OTUB2
OTUD1
OTUD3
OTUD4
OTUD5
OTUD6A
OTUD6B
OTUD7A
OTUD7B
OTULIN
OTX1
OTX2

OTX2-AS1
OTX2OS1
OVCA2
OVCH1
OVCH2
OVGP1
OVOL1
OVOL2
OXA1L
OXCT1
OXCT2
OXER1
OXGR1
OXLD1
OXNAD1
OXR1
OXSM
OXSR1
OXT
OXTR
P11
P2RX1
P2RX2
P2RX3
P2RX4
P2RX5
P2RX5-TAX1BP3
P2RX6
P2RX6P
P2RX7
P2RY1
P2RY10
P2RY12
P2RY14
P2RY2
P2RY4
P2RY6
P3H1
P3H2
P3H3
P3H4
P4HA1
P4HA2
P4HA2-AS1
P4HA3
P4HB
P4HTM
PA2G4
PAAF1
PABPC1

PABPC1L
PABPC1L2A
PABPC1L2B
PABPC1L2B-AS1
PABPC1P2
PABPC4
PABPC5-AS1
PABPN1
PABPN1L
PACRG
PACRG-AS1
PACS1
PACS2
PACSIN1
PACSIN2
PACSIN3
PADI1
PADI2
PADI3
PADI4
PADI6
PAEP
PAF1
PAFAH1B1
PAFAH1B2
PAFAH1B3
PAFAH2
PAG1
PAGE1
PAGE2
PAGE2B
PAGE3
PAGE4
PAGE5
PAGR1
PAH
PAICS
PAIP1
PAIP2
PAIP2B
PAK1
PAK2
PAK3
PAK4
PAK6
PAK7
PALB2
PALD1
PALLD
PALM

PALM2
PALM2-AKAP2
PALM3
PALMD
PAM
PAM16
PAMR1
PAN2
PAN3
PAN3-AS1
PANDAR
PANK1
PANK2
PANK3
PANK4
PANX1
PANX2
PANX3
PAOX
PAPD4
PAPD5
PAPD7
PAPL
PAPLN
PAPOLA
PAPOLB
PAPOLG
PAPPA
PAPPA2
PAPSS1
PAPSS2
PAQR3
PAQR4
PAQR5
PAQR6
PAQR7
PAQR8
PAQR9
PAQR9-AS1
PARD3
PARD3B
PARD6A
PARD6B
PARD6G
PARD6G-AS1
PARG
PARK2
PARK7
PARL
PARM1

PARN
PARP1
PARP10
PARP11
PARP12
PARP14
PARP15
PARP16
PARP2
PARP3
PARP4
PARP6
PARP8
PARP9
PARPBP
PARS2
PART1
PARVA
PARVB
PARVG
PASD1
PASK
PATE1
PATE2
PATE3
PATL1
PATL2
PATZ1
PAWR
PAX1
PAX2
PAX3
PAX4
PAX5
PAX6
PAX7
PAX8
PAX8-AS1
PAX9
PAXBP1
PAXBP1-AS1
PAXIP1
PBK
PBLD
PBRM1
PBX1
PBX2
PBX3
PBX4
PBXIP1

PC
PCAT1
PCAT18
PCAT19
PCAT29
PCAT4
PCAT5
PCAT7
PCBD1
PCBD2
PCBP1-AS1
PCBP2
PCBP3
PCBP4
PCCA
PCCA-AS1
PCCB
PCDH1
PCDH10
PCDH11X
PCDH11Y
PCDH12
PCDH15
PCDH17
PCDH18
PCDH19
PCDH20
PCDH21
PCDH24
PCDH7
PCDH8
PCDH9
PCDH9-AS3
PCDH9-AS4
PCDHA1
PCDHA10
PCDHA11
PCDHA12
PCDHA13
PCDHA2
PCDHA3
PCDHA4
PCDHA5
PCDHA6
PCDHA7
PCDHA8
PCDHA9
PCDHAC1
PCDHAC2
PCDHB1

PCDHB10
PCDHB11
PCDHB12
PCDHB13
PCDHB14
PCDHB15
PCDHB16
PCDHB17
PCDHB18
PCDHB18P
PCDHB19P
PCDHB2
PCDHB3
PCDHB4
PCDHB5
PCDHB6
PCDHB7
PCDHB8
PCDHB9
PCDHGA1
PCDHGA10
PCDHGA11
PCDHGA2
PCDHGA3
PCDHGA4
PCDHGA5
PCDHGA6
PCDHGA7
PCDHGA8
PCDHGA9
PCDHGB1
PCDHGB3
PCDHGB4
PCDHGB5
PCDHGB7
PCDHGB8P
PCDHGC3
PCDHGC5
PCDP1
PCED1A
PCED1B
PCED1B-AS1
PCF11
PCGEM1
PCGF1
PCGF2
PCGF3
PCGF5
PCGF6
PCID2

PCIF1
PCK1
PCK2
PCLO
PCM1
PCMT1
PCMTD1
PCMTD2
PCNA
PCNAP1
PCNP
PCNT
PCNX
PCNXL2
PCNXL3
PCNXL4
PCOLCE
PCOLCE-AS1
PCOLCE2
PCOTH
PCP2
PCP4
PCP4L1
PCSK1
PCSK1N
PCSK2
PCSK4
PCSK5
PCSK6
PCSK7
PCSK9
PCTP
PCYOX1
PCYOX1L
PCYT1A
PCYT1B
PCYT1B-AS1
PCYT2
PDAP1
PDCD1
PDCD10
PDCD1LG2
PDCD2
PDCD2L
PDCD4-AS1
PDCD5
PDCD6
PDCD7
PDCL
PDCL2

PDCL3
PDE10A
PDE11A
PDE12
PDE1A
PDE1B
PDE1C
PDE2A
PDE3A
PDE3B
PDE4A
PDE4B
PDE4C
PDE4D
PDE4DIP
PDE5A
PDE6A
PDE6B
PDE6C
PDE6D
PDE6G
PDE6H
PDE7A
PDE7B
PDE8A
PDE8B
PDE9A
PDF
PDGFA
PDGFB
PDGFC
PDGFD
PDGFRA
PDGFRB
PDGFRL
PDHA1
PDHA2
PDHB
PDHX
PDIA2
PDIA3
PDIA3P
PDIA4
PDIA5
PDIA6
PDIK1L
PDILT
PDK1
PDK2
PDK3

PDLIM1
PDLIM2
PDLIM3
PDLIM4
PDLIM5
PDLIM7
PDP1
PDP2
PDPK1
PDPN
PDPR
PDRG1
PDS5A
PDS5B
PDSS1
PDSS2
PDX1
PDX1-AS1
PDXDC1
PDXDC2
PDXDC2P
PDXK
PDXP
PDYN
PDZD2
PDZD3
PDZD4
PDZD7
PDZD8
PDZD9
PDZK1
PDZRN3
PDZRN4
PEA15
PEAK1
PEAR1
PEBP1
PEBP4
PECAM1
PECI
PECR
PEF1
PEG10
PEG3
PELI1
PELI2
PELI3
PELO
PELP1
PEMT

PENK
PEPD
PER1
PER3
PER4
PERM1
PERP
PES1
PET112L
PET117
PEX1
PEX10
PEX11B
PEX11G
PEX12
PEX13
PEX14
PEX16
PEX19
PEX2
PEX26
PEX3
PEX5
PEX5L
PEX6
PEX7
PF4
PF4V1
PFAS
PFDN1
PFDN2
PFDN4
PFDN5
PFDN6
PFKFB1
PFKFB2
PFKFB3
PFKFB4
PFKL
PFKP
PFN1
PFN2
PFN3
PFN4
PGA3
PGA5
PGAM1
PGAM2
PGAM4
PGAM5

PGAP1
PGAP2
PGAP3
PGBD1
PGBD2
PGBD3
PGBD4
PGBD5
PGC
PGCP
PGD
PGF
PGGT1B
PGK1
PGLS
PGLYRP1
PGLYRP2
PGLYRP4
PGM1
PGM2
PGM2L1
PGM5
PGM5-AS1
PGM5P2
PGM5P3-AS1
PGPEP1
PGPEP1L
PGR
PGRMC1
PGRMC2
PGS1
PHACTR1
PHACTR2
PHACTR3
PHACTR4
PHAX
PHB
PHB2
PHC1
PHC2
PHC3
PHEX
PHF1
PHF11
PHF12
PHF13
PHF14
PHF15
PHF17
PHF19

PHF2
PHF20
PHF20L1
PHF21A
PHF21B
PHF23
PHF24
PHF3
PHF5A
PHF6
PHF7
PHF8
PHGDH
PHGR1
PHIP
PHKA1
PHKA2
PHKA2-AS1
PHKB
PHKG1
PHLDA1
PHLDA2
PHLDA3
PHLDB1
PHLDB2
PHLDB3
PHLPP1
PHLPP2
PHOSPHO1
PHOSPHO2
PHOSPHO2-KLHL23
PHOX2A
PHOX2B
PHPT1
PHRF1
PHTF1
PHTF2
PHYH
PHYHD1
PHYHIP
PHYHIPL
PHYKPL
PI15
PI16
PI3
PI4K2A
PI4K2B
PI4KA
PI4KAP2
PI4KB

PIANP
PIAS1
PIAS2
PIAS3
PIAS4
PIBF1
PICALM
PICK1
PID1
PIDD1
PIEZO1
PIEZO2
PIF1
PIFO
PIGA
PIGBOS1
PIGC
PIGF
PIGG
PIGH
PIGK
PIGL
PIGM
PIGN
PIGO
PIGP
PIGQ
PIGR
PIGS
PIGT
PIGU
PIGV
PIGW
PIGX
PIGY
PIGZ
PIH1D1
PIK3AP1
PIK3C2A
PIK3C2B
PIK3C2G
PIK3C3
PIK3CA
PIK3CB
PIK3CD
PIK3CD-AS2
PIK3CG
PIK3IP1
PIK3R1
PIK3R2

PIK3R3
PIK3R4
PIK3R5
PIK3R6
PIKFYVE
PILRA
PILRB
PIM2
PIM3
PIN1
PIN4
PINK1
PINLYP
PINX1
PION
PIP
PIP4K2A
PIP4K2B
PIP4K2C
PIP5K1A
PIP5K1B
PIP5K1C
PIP5KL1
PIPOX
PIPSL
PIR
PIRT
PISD
PISRT1
PITHD1
PITPNA
PITPNB
PITPNC1
PITPNM1
PITPNM2
PITPNM3
PITRM1
PITRM1-AS1
PITX1
PITX2
PITX3
PIWIL1
PIWIL2
PIWIL3
PIWIL4
PJA1
PJA2
PKD1
PKD1L1
PKD1L2

PKD1L3
PKD1P6
PKD2
PKD2L1
PKD2L2
PKDCC
PKDREJ
PKHD1
PKHD1L1
PKIA
PKIB
PKIG
PKLR
PKM
PKM2
PKMYT1
PKN1
PKN2
PKN2-AS1
PKN3
PKNOX1
PKNOX2
PKP1
PKP2
PKP4
PL-5283
PLA1A
PLA2G12A
PLA2G12B
PLA2G15
PLA2G16
PLA2G1B
PLA2G2A
PLA2G2C
PLA2G2D
PLA2G2E
PLA2G2F
PLA2G3
PLA2G4A
PLA2G4C
PLA2G4D
PLA2G4E
PLA2G4F
PLA2G5
PLA2G6
PLA2G7
PLA2R1
PLAA
PLAC1
PLAC1L

PLAC2
PLAC4
PLAC8
PLAC8L1
PLAC9
PLAG1
PLAGL1
PLAGL2
PLAT
PLAU
PLB1
PLBD1
PLBD2
PLCB1
PLCB2
PLCB3
PLCB4
PLCD1
PLCD3
PLCD4
PLCE1
PLCE1-AS1
PLCE1-AS2
PLCG1
PLCG1-AS1
PLCG2
PLCH1
PLCH2
PLCL1
PLCL2
PLCXD2
PLCXD2-AS1
PLCXD3
PLCZ1
PLD1
PLD2
PLD3
PLD4
PLD5
PLD6
PLDN
PLEC
PLEC1
PLEK
PLEK2
PLEKHA1
PLEKHA2
PLEKHA3
PLEKHA4
PLEKHA5

PLEKHA6
PLEKHA7
PLEKHA8
PLEKHA8P1
PLEKHA9
PLEKHB1
PLEKHB2
PLEKHD1
PLEKHF1
PLEKHF2
PLEKHG1
PLEKHG2
PLEKHG3
PLEKHG4
PLEKHG4B
PLEKHG5
PLEKHG6
PLEKHG7
PLEKHH1
PLEKHH2
PLEKHH3
PLEKHJ1
PLEKHM1
PLEKHM1P
PLEKHM2
PLEKHM3
PLEKHN1
PLEKHO1
PLEKHO2
PLEKHS1
PLG
PLGLA
PLGRKT
PLIN1
PLIN2
PLIN3
PLIN4
PLIN5
PLK1
PLK1S1
PLK2
PLK3
PLK5
PLK5P
PLLP
PLOD1
PLOD2
PLOD3
PLP1
PLP2

PLRG1
PLS1
PLSCR2
PLSCR3
PLSCR4
PLSCR5
PLTP
PLUNC
PLVAP
PLXDC1
PLXDC2
PLXNA1
PLXNA2
PLXNA3
PLXNA4
PLXNB1
PLXNB2
PLXNB3
PLXNC1
PLXND1
PM20D1
PM20D2
PMAIP1
PMEL
PMEPA1
PMF1
PMFBP1
PML
PMM1
PMM2
PMP2
PMP22
PMPCA
PMPCB
PMS1
PMS2
PMS2CL
PMS2L11
PMS2L3
PMS2L4
PMVK
PNCK
PNISR
PNKD
PNKP
PNLDC1
PNLIP
PNLIPRP1
PNLIPRP2
PNLIPRP3

PNMA1
PNMA2
PNMA3
PNMA5
PNMAL1
PNMAL2
PNMT
PNN
PNO1
PNOC
PNP
PNPLA1
PNPLA2
PNPLA3
PNPLA4
PNPLA5
PNPLA6
PNPLA7
PNPLA8
PNPO
PNPT1
PNRC1
PNRC2
POC1A
POC1B
POC5
PODN
PODNL1
PODXL
PODXL2
POF1B
POFUT1
POFUT2
POGK
POGLUT1
POGZ
POLA1
POLA2
POLB
POLD1
POLD2
POLD3
POLD4
POLDIP2
POLDIP3
POLE
POLE2
POLE3
POLE4
POLG

POLG2
POLH
POLI
POLK
POLL
POLM
POLN
POLQ
POLR1A
POLR1B
POLR1C
POLR1D
POLR2A
POLR2B
POLR2C
POLR2D
POLR2E
POLR2F
POLR2G
POLR2H
POLR2J4
POLR2K
POLR2L
POLR2M
POLR3A
POLR3B
POLR3C
POLR3D
POLR3E
POLR3G
POLR3GL
POLR3H
POLR3K
POLRMT
POLS
POM121
POM121C
POM121L10P
POM121L12
POM121L1P
POM121L2
POM121L4P
POMC
POMGNT1
POMGNT2
POMP
POMT1
POMT2
POMZP3
PON1

PON2
PON3
POP1
POP4
POP5
POP7
POPDC2
POPDC3
POR
POSTN
POT1
POT1-AS1
POTEA
POTEC
POTEF
POTEG
POTEJ
POU1F1
POU2AF1
POU2F1
POU2F2
POU2F3
POU3F1
POU3F2
POU3F3
POU4F1
POU4F2
POU4F3
POU5F1
POU5F1B
POU6F1
POU6F2
POU6F2-AS1
PP12613
PP14571
PPA1
PPA2
PPAN-P2RY11
PPAP2A
PPAP2B
PPAP2C
PPAPDC1A
PPAPDC1B
PPAPDC3
PPARA
PPARD
PPARG
PPARGC1A
PPARGC1B
PPAT

PPBP
PPBPL2
PPCDC
PPCS
PPDPF
PPEF1
PPEF2
PPFIA1
PPFIA2
PPFIA3
PPFIA4
PPFIBP1
PPFIBP2
PPHLN1
PPIAL4A
PPIAL4G
PPIB
PPIC
PPID
PPIEL
PPIF
PPIG
PPIH
PPIL1
PPIL2
PPIL3
PPIL4
PPIL5
PPIL6
PPIP5K2
PPL
PPM1A
PPM1D
PPM1E
PPM1F
PPM1G
PPM1H
PPM1J
PPM1K
PPM1L
PPM1M
PPM1N
PPME1
PPOX
PPP1CA
PPP1CB
PPP1CC
PPP1R10
PPP1R11
PPP1R12A

PPP1R12B
PPP1R12C
PPP1R13B
PPP1R13L
PPP1R14A
PPP1R14B
PPP1R14C
PPP1R14D
PPP1R15A
PPP1R15B
PPP1R16A
PPP1R16B
PPP1R17
PPP1R1A
PPP1R1B
PPP1R1C
PPP1R2
PPP1R21
PPP1R26
PPP1R27
PPP1R2P1
PPP1R2P3
PPP1R2P9
PPP1R32
PPP1R37
PPP1R3A
PPP1R3B
PPP1R3C
PPP1R3E
PPP1R3F
PPP1R3G
PPP1R42
PPP1R7
PPP1R8
PPP1R9A
PPP1R9B
PPP2CA
PPP2CB
PPP2R1A
PPP2R1B
PPP2R2A
PPP2R2B
PPP2R2B-IT1
PPP2R2C
PPP2R2D
PPP2R3A
PPP2R3C
PPP2R4
PPP2R5A
PPP2R5B

PPP2R5C
PPP2R5D
PPP2R5E
PPP3CA
PPP3CB
PPP3CB-AS1
PPP3CC
PPP3R1
PPP3R2
PPP4C
PPP4R1
PPP4R1-AS1
PPP4R1L
PPP4R2
PPP4R3A
PPP4R4
PPP5C
PPP5D1
PPP6C
PPP6R1
PPP6R2
PPP6R3
PPPDE2
PPRC1
PPT1
PPT2
PPTC7
PPWD1
PPY
PPYR1
PQLC1
PQLC2
PQLC2L
PQLC3
PRAC
PRAC1
PRAC2
PRAF2
PRAGMIN
PRAM1
PRAME
PRAMEF1
PRAMEF11
PRAMEF12
PRAMEF2
PRAMEF20
PRAMEF4
PRAMEF6
PRAP1
PRB1

PRB3
PRB4
PRC1
PRCAT47
PRCC
PRCD
PRCP
PRDM1
PRDM10
PRDM11
PRDM12
PRDM13
PRDM14
PRDM15
PRDM16
PRDM2
PRDM4
PRDM5
PRDM6
PRDM7
PRDM8
PRDM9
PRDX1
PRDX2
PRDX3
PRDX4
PRDX5
PRDX6
PRDXDD1P
PREB
PRELID2
PRELP
PREP
PREPL
PREX1
PREX2
PRF1
PRG2
PRG3
PRG4
PRH1-PRR4
PRH2
PRHOXNB
PRIC285
PRICKLE1
PRICKLE2
PRICKLE2-AS1
PRICKLE2-AS3
PRICKLE3
PRICKLE4

PRIM1
PRIM2
PRIMA1
PRIMPOL
PRKAA1
PRKAA2
PRKAB1
PRKAB2
PRKACA
PRKACB
PRKACG
PRKAG1
PRKAG2
PRKAG2-AS1
PRKAG3
PRKAR1A
PRKAR1B
PRKAR2A
PRKAR2A-AS1
PRKAR2B
PRKCA
PRKCB
PRKCD
PRKCDBP
PRKCE
PRKCG
PRKCH
PRKCI
PRKCQ
PRKCQ-AS1
PRKCSH
PRKCZ
PRKD1
PRKD2
PRKD3
PRKDC
PRKG1
PRKG1-AS1
PRKG2
PRKRA
PRKRIP1
PRKRIR
PRKX
PRKXP1
PRKY
PRLH
PRLHR
PRLR
PRM1
PRM2

PRM3
PRMT1
PRMT10
PRMT2
PRMT3
PRMT5
PRMT6
PRMT7
PRMT8
PRMT9
PRNCR1
PRND
PRNP
PRNT
PRO1768
PROC
PROCA1
PROCR
PRODH
PRODH2
PROK1
PROK2
PROKR1
PROKR2
PROL1
PROM1
PROM2
PROP1
PRORSD1P
ProSAPiP1
PROSC
PROSER1
PROSER2
PROSER2-AS1
PROSER3
PROX1
PROX1-AS1
PROX2
PROZ
PRPF18
PRPF19
PRPF3
PRPF31
PRPF38B
PRPF39
PRPF4
PRPF40A
PRPF40B
PRPF4B
PRPF6

PRPF8
PRPH
PRPH2
PRPS1
PRPS1L1
PRPS2
PRPSAP1
PRPSAP2
PRR11
PRR12
PRR13
PRR14
PRR14L
PRR15
PRR15L
PRR16
PRR18
PRR19
PRR20A
PRR22
PRR23A
PRR23B
PRR23C
PRR25
PRR3
PRR30
PRR32
PRR34-AS1
PRR35
PRR36
PRR4
PRR5
PRR5-ARHGAP8
PRR5L
PRR7
PRR7-AS1
PRR9
PRRC1
PRRC2B
PRRC2C
PRRG1
PRRG2
PRRT1
PRRT2
PRRT3
PRRT4
PRRX1
PRRX2
PRRX2-AS1
PRSS1

PRSS12
PRSS16
PRSS21
PRSS22
PRSS23
PRSS27
PRSS3
PRSS33
PRSS35
PRSS36
PRSS37
PRSS41
PRSS45
PRSS46
PRSS48
PRSS50
PRSS55
PRSS56
PRSS8
PRSSL1
PRTFDC1
PRTG
PRTN3
PRUNE2
PRX
PSAP
PSAPL1
PSAT1
PSCA
PSD
PSD2
PSD3
PSD4
PSEN1
PSEN2
PSENEN
PSG1
PSG10
PSG2
PSG3
PSG4
PSG8
PSG9
PSIP1
psiTPTE22
PSKH1
PSKH2
PSMA1
PSMA2
PSMA3

PSMA3-AS1
PSMA4
PSMA5
PSMA6
PSMA7
PSMA8
PSMB1
PSMB10
PSMB11
PSMB2
PSMB3
PSMB5
PSMB6
PSMB7
PSMB8
PSMB8-AS1
PSMB9
PSMC1
PSMC2
PSMC3IP
PSMC4
PSMC5
PSMC6
PSMD1
PSMD10
PSMD11
PSMD12
PSMD13
PSMD14
PSMD2
PSMD3
PSMD4
PSMD5
PSMD6
PSMD7
PSMD8
PSMD9
PSME1
PSME2
PSME3
PSME4
PSMF1
PSMG1
PSMG2
PSMG3
PSMG3-AS1
PSMG4
PSORS1C1
PSORS1C3
PSPC1

PSPH
PSPN
PSRC1
PSTK
PSTPIP1
PSTPIP2
PTAFR
PTAR1
PTBP1
PTBP2
PTBP3
PTCD1
PTCD2
PTCD3
PTCH1
PTCH2
PTCHD1
PTCHD1-AS
PTCHD2
PTCHD3
PTCHD4
PTCRA
PTDSS1
PTDSS2
PTEN
PTER
PTF1A
PTGDR
PTGDR2
PTGDS
PTGER1
PTGER2
PTGER3
PTGER4
PTGES
PTGES2
PTGES3
PTGES3L-AARSD1
PTGFR
PTGFRN
PTGIR
PTGIS
PTGR1
PTGR2
PTGS1
PTGS2
PTH1R
PTH2
PTH2R
PTHLH

PTK2
PTK2B
PTK6
PTK7
PTMA
PTMS
PTN
PTOV1
PTOV1-AS1
PTP4A1
PTP4A2
PTP4A3
PTPDC1
PTPLA
PTPLAD1
PTPLB
PTPMT1
PTPN1
PTPN11
PTPN12
PTPN13
PTPN14
PTPN18
PTPN2
PTPN21
PTPN22
PTPN23
PTPN3
PTPN4
PTPN5
PTPN6
PTPN9
PTPRA
PTPRB
PTPRC
PTPRCAP
PTPRD
PTPRD-AS2
PTPRE
PTPRF
PTPRG
PTPRH
PTPRJ
PTPRK
PTPRM
PTPRN
PTPRN2
PTPRO
PTPRQ
PTPRR

PTPRS
PTPRT
PTPRU
PTPRV
PTPRZ1
PTRF
PTRH1
PTRH2
PTRHD1
PTS
PTTG1
PTTG1IP
PTX3
PUF60
PUM1
PUM2
PURA
PURB
PURG
PUS1
PUS10
PUS3
PUS7
PUS7L
PUSL1
PVALB
PVR
PVRL1
PVRL2
PVRL3
PVRL4
PVT1
PWP1
PWP2
PWRN1
PWRN4
PWWP2A
PWWP2B
PXDN
PXDNL
PXK
PXMP2
PXMP3
PXMP4
PXN
PXN-AS1
PXT1
PXYLP1
PYCARD
PYCARD-AS1

PYCR1
PYCR2
PYCRL
PYGB
PYGL
PYGM
PYGO1
PYGO2
PYHIN1
PYROXD1
PYROXD2
PYY
PYY2
PZP
QARS
QDPR
QKI
QPCT
QPCTL
QPRT
QRFP
QRFPR
QRICH1
QRICH2
QRSL1
QSER1
QSOX1
QSOX2
QTRT1
QTRTD1
R3HCC1
R3HCC1L
R3HDM1
R3HDM2
R3HDM4
R3HDML
RAB10
RAB11A
RAB11B
RAB11FIP1
RAB11FIP2
RAB11FIP3
RAB11FIP4
RAB11FIP5
RAB12
RAB13
RAB14
RAB15
RAB17
RAB18

RAB19
RAB1A
RAB1B
RAB20
RAB21
RAB22A
RAB23
RAB26
RAB27A
RAB27B
RAB28
RAB29
RAB2A
RAB2B
RAB30
RAB31
RAB32
RAB33A
RAB33B
RAB34
RAB35
RAB36
RAB37
RAB38
RAB39
RAB39A
RAB39B
RAB3B
RAB3C
RAB3D
RAB3GAP1
RAB3GAP2
RAB3IL1
RAB3IP
RAB40A
RAB40B
RAB40C
RAB41
RAB42
RAB43
RAB44
RAB4A
RAB4B
RAB5A
RAB5B
RAB5C
RAB6A
RAB6B
RAB6C
RAB7A

RAB7L1
RAB8A
RAB8B
RAB9B
RABAC1
RABEP1
RABEP2
RABEPK
RABGAP1
RABGAP1L
RABGEF1
RABGGTA
RABGGTB
RABIF
RABL3
RABL4
RABL5
RABL6
RAC1
RAC2
RAC3
RACGAP1
RACGAP1P
RAD1
RAD17
RAD18
RAD21
RAD21L1
RAD23A
RAD23B
RAD50
RAD51
RAD51-AS1
RAD51AP2
RAD51B
RAD51C
RAD51D
RAD51L1
RAD51L3
RAD51L3-RFFL
RAD52
RAD54B
RAD54L
RAD54L2
RAD9A
RAD9B
RADIL
RAE1
RAET1E
RAET1G

RAET1K
RAF1
RAG1
RAG1AP1
RAG2
RAGE
RAI1
RAI14
RAI2
RALA
RALB
RALBP1
RALGAPA1
RALGAPA1P
RALGAPA2
RALGAPB
RALGDS
RALGPS1
RALGPS2
RALY
RALY-AS1
RALYL
RAMP1
RAMP2
RAMP2-AS1
RAMP3
RAN
RANBP1
RANBP10
RANBP17
RANBP2
RANBP3
RANBP3L
RANBP6
RANBP9
RANGAP1
RANGRF
RAP1A
RAP1B
RAP1GAP
RAP1GAP2
RAP1GDS1
RAP2A
RAP2B
RAP2C
RAPGEF1
RAPGEF2
RAPGEF3
RAPGEF4
RAPGEF5

RAPGEF6
RAPGEFL1
RAPSN
RARA
RARA-AS1
RARB
RARG
RARRES1
RARRES2
RARRES3
RARS2
RASA1
RASA2
RASA3
RASA4CP
RASAL1
RASAL2
RASAL3
RASD1
RASD2
RASEF
RASGEF1A
RASGEF1B
RASGEF1C
RASGRF1
RASGRF2
RASGRF2-AS1
RASGRP1
RASGRP2
RASGRP3
RASGRP4
RASIP1
RASL10A
RASL10B
RASL11A
RASL11B
RASL12
RASSF1
RASSF10
RASSF2
RASSF3
RASSF4
RASSF5
RASSF6
RASSF7
RASSF8
RASSF8-AS1
RASSF9
RAVER1
RAVER2

RAX
RB1
RB1CC1
RBAK
RBBP4
RBBP5
RBBP6
RBBP7
RBBP8
RBBP9
RBCK1
RBFA
RBFADN
RBFOX1
RBFOX2
RBFOX3
RBKS
RBL1
RBL2
RBM10
RBM12
RBM12B
RBM14
RBM15
RBM15B
RBM16
RBM17
RBM18
RBM19
RBM20
RBM22
RBM23
RBM24
RBM25
RBM26
RBM27
RBM28
RBM3
RBM33
RBM34
RBM38
RBM39
RBM4
RBM41
RBM42
RBM43
RBM44
RBM45
RBM46
RBM47

RBM4B
RBM5
RBM6
RBM8A
RBM9
RBMS1
RBMS2
RBMS3
RBMX
RBMX2
RBMXL1
RBMXL3
RBMY2FP
RBP1
RBP3
RBP4
RBP5
RBP7
RBPJ
RBPJL
RBPMS
RBPMS2
RBX1
RC3H2
RCAN1
RCAN2
RCAN3
RCBTB1
RCBTB2
RCC1
RCC2
RCCD1
RCE1
RCHY1
RCL1
RCN2
RCN3
RCOR1
RCOR2
RCOR3
RCSD1
RCVRN
RD3
RDBP
RDH10
RDH10-AS1
RDH11
RDH12
RDH13
RDH14

RDH16
RDH8
RDM1
RDX
REC8
RECK
RECQL4
RECQL5
REEP1
REEP2
REEP3
REEP5
REEP6
REG1A
REG1B
REG1P
REG3A
REG3G
REG4
REL
RELA
RELB
RELL1
RELL2
RELN
RELT
REM1
REM2
REN
RENBP
REP15
REPIN1
REPS1
REPS2
RER1
RERE
REREP3
RERG
RERG-AS1
RESP18
REST
RET
RETN
RETNLB
RETSAT
REV1
REV3L
REXO1
REXO2
REXO4

RFC1
RFC2
RFC3
RFC4
RFC5
RFESD
RFFL
RFK
RFNG
RFPL1S
RFPL2
RFPL3
RFPL3S
RFPL4AL1
RFPL4B
RFT1
RFTN1
RFTN2
RFWD2
RFWD3
RFX1
RFX2
RFX3
RFX4
RFX5
RFX6
RFX7
RFX8
RFXANK
RFXAP
RG9MTD1
RG9MTD2
RGCC
RGL1
RGL2
RGL3
RGL4
RGMA
RGMB
RGN
RGNEF
RGP1
RGPD1
RGPD5
RGPD8
RGR
RGS10
RGS11
RGS12
RGS13

RGS14
RGS16
RGS17
RGS2
RGS20
RGS22
RGS3
RGS4
RGS5
RGS6
RGS7
RGS7BP
RGS8
RGS9
RGS9BP
RGSL1
RHBDD1
RHBDD2
RHBDD3
RHBDF1
RHBDF2
RHBDL1
RHBDL2
RHBDL3
RHBG
RHCE
RHCG
RHD
RHEB
RHEBL1
RHO
RHOA
RHOB
RHOBTB1
RHOBTB2
RHOBTB3
RHOC
RHOD
RHOF
RHOG
RHOH
RHOJ
RHOQ
RHOT1
RHOT2
RHOU
RHOV
RHPN1
RHPN2
RIBC1

RIBC2
RIC1
RIC3
RIC8A
RIC8B
RICH2
RICS
RICTOR
RIF1
RIIAD1
RILP
RILPL1
RILPL2
RIMBP2
RIMKLA
RIMKLB
RIMS1
RIMS2
RIMS3
RIMS4
RIN1
RIN2
RIN3
RING1
RINL
RINT1
RIOK1
RIOK2
RIOK3
RIPK1
RIPK3
RIPK4
RIPPLY1
RIPPLY2
RIPPLY3
RIT1
RIT2
RLBP1
RLF
RLIM
RLN1
RLN3
RLTPR
RMDN1
RMDN2
RMDN3
RMI2
RMND1
RMND5A
RMND5B

RMST
RNASE1
RNASE10
RNASE12
RNASE13
RNASE2
RNASE4
RNASE6
RNASE7
RNASE8
RNASE9
RNASEH1
RNASEH2A
RNASEH2B
RNASEH2B-AS1
RNASEH2C
RNASEK
RNASEK-C17orf49
RNASEL
RNASEN
RNASET2
RND1
RND2
RND3
RNF10
RNF103
RNF103-CHMP3
RNF11
RNF111
RNF112
RNF113A
RNF114
RNF115
RNF121
RNF122
RNF123
RNF125
RNF126
RNF126P1
RNF128
RNF13
RNF130
RNF133
RNF135
RNF138
RNF139
RNF139-AS1
RNF14
RNF141
RNF144A

RNF144B
RNF145
RNF146
RNF149
RNF150
RNF151
RNF152
RNF157
RNF157-AS1
RNF160
RNF165
RNF166
RNF167
RNF168
RNF169
RNF17
RNF170
RNF175
RNF180
RNF182
RNF183
RNF185
RNF186
RNF187
RNF19A
RNF19B
RNF2
RNF20
RNF207
RNF212
RNF212B
RNF213
RNF214
RNF215
RNF216
RNF216-IT1
RNF216L
RNF216P1
RNF217
RNF217-AS1
RNF219
RNF219-AS1
RNF220
RNF222
RNF24
RNF25
RNF26
RNF31
RNF32
RNF34

RNF38
RNF39
RNF4
RNF40
RNF41
RNF43
RNF44
RNF5
RNF5P1
RNF6
RNF7
RNF8
RNFT1
RNFT2
RNGTT
RNH1
RNLS
RNMTL1
RNPC3
RNPEP
RNPEPL1
RNPS1
RNU11
RNU5E
RNU5E-1
RNU5F-1
RNU6-19P
RNU6-2
RNU6-28P
RNU6-52P
RNU6-66P
RNU6-71P
RNU6-76P
RNU6-79P
RNU6-81P
ROBO1
ROBO2
ROBO3
ROBO4
ROCK1
ROCK2
ROD1
ROGDI
ROM1
ROMO1
ROPN1
ROPN1B
ROPN1L
ROR1
ROR1-AS1

ROR2
RORA
RORA-AS1
RORB
RORC
ROS1
RP1
RP1-177G6.2
RP11-529I10.4
RP1L1
RP9
RP9P
RPA1
RPA2
RPA3
RPAIN
RPAP1
RPAP2
RPAP3
RPARP-AS1
RPE
RPE65
RPEL1
RPF1
RPF2
RPGR
RPGRIP1
RPGRIP1L
RPH3A
RPH3AL
RPIA
RPL10
RPL10A
RPL10L
RPL11
RPL13
RPL13A
RPL13AP17
RPL13AP20
RPL13AP3
RPL13AP5
RPL13P5
RPL14
RPL15
RPL17
RPL18
RPL18AP3
RPL19
RPL21
RPL22

RPL22L1
RPL23
RPL23A
RPL23AP53
RPL23AP7
RPL23AP82
RPL24
RPL26
RPL26L1
RPL27A
RPL28
RPL29
RPL3
RPL30
RPL31
RPL31P11
RPL32
RPL32P3
RPL34-AS1
RPL35
RPL35A
RPL36
RPL36A
RPL36A-HNRNPH2
RPL36AL
RPL37
RPL37A
RPL38
RPL39
RPL41
RPL5
RPL6
RPL7
RPL7L1
RPL8
RPL9
RPLP0
RPLP0P2
RPLP1
RPLP2
RPN1
RPN2
RPP14
RPP21
RPP25
RPP25L
RPP30
RPP38
RPP40
RPPH1

RPRD1A
RPRD1B
RPRD2
RPRM
RPRML
RPS10
RPS10P7
RPS11
RPS12
RPS13
RPS14
RPS15
RPS16
RPS18
RPS18P9
RPS19
RPS19BP1
RPS2
RPS20
RPS21
RPS23
RPS24
RPS25
RPS26
RPS27
RPS27A
RPS27L
RPS28
RPS29
RPS2P32
RPS3
RPS3A
RPS4X
RPS4Y1
RPS4Y2
RPS5
RPS6
RPS6KA1
RPS6KA2
RPS6KA2-IT1
RPS6KA3
RPS6KA4
RPS6KA5
RPS6KA6
RPS6KB2
RPS6KC1
RPS6KL1
RPS7
RPS8
RPS9

RPSA
RPSAP52
RPSAP58
RPSAP9
RPTOR
RPUSD1
RPUSD2
RPUSD3
RPUSD4
RQCD1
RRAD
RRAGA
RRAGB
RRAGC
RRAGD
RRAS
RRAS2
RRBP1
RREB1
RRM1
RRM2
RRM2B
RRN3
RRN3P1
RRN3P2
RRN3P3
RRNAD1
RRP1
RRP12
RRP15
RRP1B
RRP7A
RRP7B
RRP8
RRP9
RRS1
RRS1-AS1
RS1
RSAD1
RSAD2
RSBN1
RSBN1L
RSF1
RSL1D1
RSL24D1
RSPH1
RSPH10B
RSPH14
RSPH3
RSPH4A

RSPH6A
RSPH9
RSPO1
RSPO2
RSPO3
RSPO4
RSPRY1
RSRC1
RSRP1
RSU1
RSU1P2
RTBDN
RTDR1
RTEL1
RTEL1-TNFRSF6B
RTF1
RTKN
RTKN2
RTL1
RTN1
RTN2
RTN3
RTN4
RTN4IP1
RTN4R
RTN4RL1
RTN4RL2
RTP1
RTP2
RTP3
RTP4
RTTN
RUFY1
RUFY2
RUFY3
RUFY4
RUNDC1
RUNDC2A
RUNDC2C
RUNDC3A
RUNDC3B
RUNX1
RUNX1T1
RUNX2
RUNX3
RUSC1
RUSC2
RUVBL1
RUVBL1-AS1
RWDD1

RWDD2A
RWDD2B
RWDD3
RXFP1
RXFP2
RXFP3
RXFP4
RXRA
RXRB
RXRG
RYBP
RYK
RYR1
RYR2
RYR3
S100A10
S100A11
S100A12
S100A13
S100A14
S100A16
S100A2
S100A3
S100A5
S100A7
S100A7A
S100A7L2
S100A8
S100A9
S100B
S100P
S100PBP
S100Z
S1PR1
S1PR2
S1PR3
S1PR4
S1PR5
SAA1
SAA2
SAA4
SAAL1
SAC3D1
SACM1L
SACS
SAE1
SAFB
SAFB2
SAG
SAGE1

SALL1
SALL2
SALL3
SALL4
SAMD1
SAMD10
SAMD11
SAMD12
SAMD13
SAMD14
SAMD15
SAMD3
SAMD4A
SAMD4B
SAMD5
SAMD7
SAMD8
SAMD9
SAMD9L
SAMHD1
SAMM50
SAMSN1
SAP130
SAP18
SAP25
SAP30
SAP30BP
SAP30L
SAP30L-AS1
SAPCD2
SAPS1
SAPS2
SAPS3
SAR1A
SAR1B
SARAF
SARDH
SARM1
SARNP
SARS
SART1
SART3
SASH1
SASH3
SASS6
SAT1
SATB1
SATB2
SATB2-AS1
SAV1

SAXO1
SAXO2
SAYSD1
SBDS
SBDSP
SBDSP1
SBF1
SBF2
SBF2-AS1
SBK1
SBK2
SBK3
SBNO1
SBNO2
SBSN
SBSPON
SC4MOL
SC5D
SC5DL
SC65
SCAF1
SCAF11
SCAF4
SCAF8
SCAI
SCAMP1
SCAMP1-AS1
SCAMP2
SCAMP3
SCAMP4
SCAMP5
SCAND1
SCAND2
SCAND3
SCAP
SCAPER
SCARA3
SCARA5
SCARB1
SCARB2
SCARF1
SCARF2
SCARNA10
SCARNA11
SCARNA12
SCARNA16
SCARNA2
SCARNA27
SCARNA4
SCARNA8

SCARNA9
SCARNA9L
SCART1
SCCPDH
SCD
SCD5
SCEL
SCFD1
SCFD2
SCG2
SCG3
SCG5
SCGB1A1
SCGB1C1
SCGB1D1
SCGB1D2
SCGB1D4
SCGB2A1
SCGB2A2
SCGB3A1
SCGB3A2
SCGN
SCHIP1
SCHLAP1
SCIMP
SCIN
SCLT1
SCLY
SCMH1
SCML1
SCML2
SCML4
SCN10A
SCN11A
SCN1A
SCN1B
SCN2A
SCN2B
SCN3A
SCN3B
SCN4A
SCN4B
SCN5A
SCN7A
SCN8A
SCN9A
SCNN1A
SCNN1B
SCNN1D
SCNN1G

SCO1
SCO2
SCOC
SCOC-AS1
SCP2
SCPEP1
SCRG1
SCRIB
SCRN1
SCRN2
SCRT1
SCRT2
SCT
SCTR
SCUBE1
SCUBE2
SCUBE3
SCYL1
SCYL2
SCYL3
SDAD1
SDC1
SDC2
SDC3
SDC4
SDC4P
SDCBP
SDCBP2
SDCCAG10
SDCCAG3
SDCCAG8
SDF2
SDF2L1
SDF4
SDHA
SDHAF1
SDHAF2
SDHAP1
SDHAP2
SDHAP3
SDHB
SDHC
SDHD
SDK1
SDK2
SDR16C5
SDR16C6P
SDR39U1
SDR42E1
SDR9C7

SDS
SDSL
SEBOX
SEC1
SEC11A
SEC11C
SEC13
SEC14L1
SEC14L2
SEC14L3
SEC14L4
SEC14L5
SEC14L6
SEC16A
SEC16B
SEC1P
SEC22A
SEC22B
SEC22C
SEC23A
SEC23B
SEC23IP
SEC24A
SEC24C
SEC24D
SEC31A
SEC31B
SEC61A1
SEC61A2
SEC61B
SEC61G
SEC62
SEC63
SECISBP2
SECISBP2L
SECTM1
SEH1L
SEL1L
SEL1L2
SEL1L3
SELE
SELI
SELK
SELM
SELO
SELP
SELPLG
SELS
SELT
SELV

SEMA3A
SEMA3B
SEMA3C
SEMA3D
SEMA3E
SEMA3F
SEMA3G
SEMA4A
SEMA4B
SEMA4C
SEMA4D
SEMA4F
SEMA4G
SEMA5A
SEMA5B
SEMA6A
SEMA6B
SEMA6C
SEMA6D
SEMA7A
SEMG1
SEMG2
SENCR
SENP1
SENP2
SENP3
SENP5
SENP6
SENP7
SENP8
15-Sep
SEPHS1
SEPHS2
SEPN1
SEPP1
SEPSECS
SEPSECS-AS1
1-Sep
10-Sep
11-Sep
12-Sep
13-Sep
14-Sep
2-Sep
3-Sep
4-Sep
SEPT4-AS1
5-Sep
6-Sep
7-Sep

SEPT7P2
8-Sep
9-Sep
SEPW1
SEPX1
SERAC1
SERBP1
SERF2
SERGEF
SERHL
SERHL2
SERINC1
SERINC2
SERINC3
SERINC4
SERINC5
SERP1
SERP2
SERPINA1
SERPINA10
SERPINA12
SERPINA3
SERPINA4
SERPINA5
SERPINA6
SERPINA9
SERPINB1
SERPINB11
SERPINB12
SERPINB13
SERPINB2
SERPINB5
SERPINB6
SERPINB7
SERPINB8
SERPINB9
SERPINB9P1
SERPINC1
SERPIND1
SERPINE1
SERPINE2
SERPINE3
SERPINF1
SERPINF2
SERPING1
SERPINH1
SERPINI1
SERPINI2
SERTAD1
SERTAD2

SERTAD3
SERTAD4
SERTM1
SESN1
SESN2
SESN3
SESTD1
SET
SETBP1
SETD1A
SETD1B
SETD2
SETD3
SETD4
SETD5
SETD6
SETD7
SETD8
SETD9
SETDB1
SETDB2
SETMAR
SETX
SEZ6
SEZ6L
SEZ6L2
SF1
SF3A1
SF3A2
SF3A3
SF3B1
SF3B14
SF3B2
SF3B3
SF3B4
SF3B5
SF4
SFI1
SFMBT1
SFMBT2
SFPQ
SFRP1
SFRP2
SFRP4
SFRP5
SFRS1
SFRS11
SFRS12
SFRS12IP1
SFRS13A

SFRS13B
SFRS14
SFRS15
SFRS18
SFRS2B
SFRS2IP
SFRS3
SFRS4
SFRS5
SFRS6
SFRS7
SFRS8
SFRS9
SFSWAP
SFT2D1
SFT2D2
SFT2D3
SFTA2
SFTA3
SFTPA1
SFTPA2
SFTPB
SFTPC
SFTPD
SFXN1
SFXN2
SFXN3
SFXN4
SFXN5
SGCA
SGCD
SGCE
SGCG
SGCZ
SGEF
SGIP1
SGK1
SGK196
SGK2
SGK223
SGK269
SGK3
SGK494
SGMS1
SGMS2
SGOL1
SGOL2
SGPL1
SGPP1
SGPP2

SGSM1
SGSM2
SGSM3
SGTA
SGTB
SH2B1
SH2B2
SH2B3
SH2D1A
SH2D1B
SH2D2A
SH2D3A
SH2D3C
SH2D4A
SH2D4B
SH2D5
SH2D6
SH2D7
SH3BGR
SH3BGRL
SH3BGRL2
SH3BGRL3
SH3BP1
SH3BP2
SH3BP4
SH3BP5
SH3BP5L
SH3D19
SH3D21
SH3GL1
SH3GL2
SH3GL3
SH3GLB1
SH3GLB2
SH3KBP1
SH3PXD2A
SH3PXD2B
SH3RF1
SH3RF2
SH3RF3
SH3TC1
SH3TC2
SH3YL1
SHANK1
SHANK2
SHANK3
SHARPIN
SHB
SHBG
SHC1

SHC2
SHC3
SHC4
SHCBP1
SHCBP1L
SHD
SHE
SHF
SHFM1
SHH
SHISA2
SHISA3
SHISA4
SHISA5
SHISA6
SHISA7
SHISA9
SHKBP1
SHMT1
SHMT2
SHOC2
SHOX2
SHPK
SHPRH
SHQ1
SHROOM1
SHROOM2
SHROOM3
SHROOM4
SHTN1
SIAE
SIAH1
SIAH2
SIAH3
SIDT1
SIDT2
SIGIRR
SIGLEC1
SIGLEC10
SIGLEC12
SIGLEC14
SIGLEC15
SIGLEC16
SIGLEC5
SIGLEC6
SIGLEC8
SIGLEC9
SIGLECL1
SIGLECP3
SIK1

SIK2
SIK3
SIKE1
SIL1
SILV
SIM1
SIM2
SIMC1
SIN3A
SIN3B
SIP1
SIPA1
SIPA1L1
SIPA1L2
SIPA1L3
SIRPA
SIRPB1
SIRPB2
SIRPD
SIRPG
SIRT1
SIRT2
SIRT3
SIRT4
SIRT5
SIRT6
SIRT7
SIT1
SIVA1
SIX1
SIX2
SIX3
SIX3-AS1
SIX4
SIX5
SIX6
SKA1
SKA3
SKAP1
SKAP2
SKI
SKIDA1
SKIL
SKINTL
SKIV2L
SKIV2L2
SKOR1
SKOR2
SKP1
SKP2

SLA
SLA2
SLAIN1
SLAIN2
SLAMF1
SLAMF6
SLAMF7
SLAMF8
SLAMF9
SLC10A2
SLC10A3
SLC10A4
SLC10A5
SLC10A6
SLC10A7
SLC11A1
SLC11A2
SLC12A1
SLC12A2
SLC12A3
SLC12A4
SLC12A5
SLC12A6
SLC12A7
SLC12A8
SLC12A9
SLC13A1
SLC13A2
SLC13A3
SLC13A4
SLC13A5
SLC14A1
SLC14A2
SLC14A2-AS1
SLC15A1
SLC15A2
SLC15A3
SLC15A4
SLC15A5
SLC16A1
SLC16A10
SLC16A11
SLC16A12
SLC16A13
SLC16A14
SLC16A2
SLC16A3
SLC16A4
SLC16A5
SLC16A7

SLC16A8
SLC16A9
SLC17A1
SLC17A2
SLC17A3
SLC17A4
SLC17A5
SLC17A6
SLC17A7
SLC17A8
SLC17A9
SLC18A1
SLC18A2
SLC18A3
SLC19A1
SLC19A2
SLC19A3
SLC1A1
SLC1A2
SLC1A3
SLC1A4
SLC1A5
SLC1A6
SLC1A7
SLC20A1
SLC20A2
SLC22A1
SLC22A10
SLC22A11
SLC22A12
SLC22A13
SLC22A14
SLC22A15
SLC22A16
SLC22A17
SLC22A18
SLC22A18AS
SLC22A2
SLC22A20
SLC22A23
SLC22A24
SLC22A25
SLC22A3
SLC22A31
SLC22A4
SLC22A5
SLC22A6
SLC22A7
SLC22A8
SLC23A1

SLC23A2
SLC23A3
SLC24A1
SLC24A2
SLC24A3
SLC24A4
SLC24A6
SLC25A1
SLC25A10
SLC25A11
SLC25A12
SLC25A13
SLC25A14
SLC25A15
SLC25A16
SLC25A17
SLC25A18
SLC25A19
SLC25A2
SLC25A20
SLC25A21
SLC25A21-AS1
SLC25A22
SLC25A24
SLC25A25
SLC25A25-AS1
SLC25A26
SLC25A27
SLC25A28
SLC25A29
SLC25A3
SLC25A30
SLC25A31
SLC25A32
SLC25A33
SLC25A34
SLC25A35
SLC25A36
SLC25A37
SLC25A38
SLC25A39
SLC25A4
SLC25A40
SLC25A41
SLC25A42
SLC25A43
SLC25A44
SLC25A45
SLC25A46
SLC25A47

SLC25A48
SLC25A51
SLC25A53
SLC26A1
SLC26A10
SLC26A11
SLC26A2
SLC26A3
SLC26A4
SLC26A5
SLC26A6
SLC26A7
SLC26A8
SLC26A9
SLC27A1
SLC27A2
SLC27A3
SLC27A4
SLC27A5
SLC27A6
SLC28A1
SLC28A3
SLC29A1
SLC29A2
SLC29A3
SLC29A4
SLC2A1
SLC2A1-AS1
SLC2A10
SLC2A11
SLC2A12
SLC2A13
SLC2A14
SLC2A2
SLC2A3
SLC2A4
SLC2A4RG
SLC2A5
SLC2A6
SLC2A7
SLC2A8
SLC2A9
SLC30A1
SLC30A10
SLC30A2
SLC30A3
SLC30A4
SLC30A5
SLC30A6
SLC30A7

SLC30A8
SLC30A9
SLC31A2
SLC32A1
SLC33A1
SLC34A1
SLC34A2
SLC34A3
SLC35A1
SLC35A2
SLC35A3
SLC35A4
SLC35B1
SLC35B2
SLC35B3
SLC35B4
SLC35C1
SLC35C2
SLC35D1
SLC35D2
SLC35D3
SLC35E1
SLC35E2
SLC35E2B
SLC35E3
SLC35E4
SLC35F1
SLC35F2
SLC35F3
SLC35F4
SLC35F5
SLC35G1
SLC35G4
SLC36A1
SLC36A2
SLC36A3
SLC36A4
SLC37A1
SLC37A2
SLC37A3
SLC37A4
SLC38A1
SLC38A10
SLC38A11
SLC38A2
SLC38A3
SLC38A4
SLC38A5
SLC38A6
SLC38A7

SLC38A8
SLC38A9
SLC39A1
SLC39A10
SLC39A11
SLC39A12
SLC39A12-AS1
SLC39A13
SLC39A14
SLC39A3
SLC39A4
SLC39A6
SLC39A7
SLC39A8
SLC39A9
SLC3A1
SLC3A2
SLC40A1
SLC41A1
SLC41A2
SLC41A3
SLC43A1
SLC43A2
SLC43A3
SLC44A1
SLC44A2
SLC44A3
SLC44A4
SLC44A5
SLC45A1
SLC45A2
SLC45A3
SLC45A4
SLC46A1
SLC46A2
SLC46A3
SLC47A1
SLC47A2
SLC48A1
SLC4A1
SLC4A10
SLC4A11
SLC4A1AP
SLC4A2
SLC4A3
SLC4A4
SLC4A5
SLC4A7
SLC4A8
SLC4A9

SLC50A1
SLC51A
SLC52A3
SLC5A1
SLC5A10
SLC5A11
SLC5A12
SLC5A2
SLC5A3
SLC5A4
SLC5A5
SLC5A6
SLC5A7
SLC5A8
SLC5A9
SLC6A1
SLC6A1-AS1
SLC6A10P
SLC6A11
SLC6A12
SLC6A13
SLC6A14
SLC6A15
SLC6A17
SLC6A18
SLC6A19
SLC6A2
SLC6A20
SLC6A3
SLC6A4
SLC6A5
SLC6A6
SLC6A7
SLC6A8
SLC6A9
SLC7A1
SLC7A10
SLC7A11
SLC7A13
SLC7A14
SLC7A2
SLC7A3
SLC7A4
SLC7A5
SLC7A5P1
SLC7A5P2
SLC7A6
SLC7A6OS
SLC7A7
SLC7A8

SLC7A9
SLC8A1
SLC8A1-AS1
SLC8A2
SLC8A3
SLC8B1
SLC9A1
SLC9A10
SLC9A11
SLC9A2
SLC9A3
SLC9A3R1
SLC9A3R2
SLC9A4
SLC9A5
SLC9A6
SLC9A7
SLC9A8
SLC9A9
SLC9B1
SLC9B2
SLC9C1
SLC9C2
SLCO1A2
SLCO1B1
SLCO1B3
SLCO1B7
SLCO1C1
SLCO2A1
SLCO2B1
SLCO3A1
SLCO4A1
SLCO4A1-AS1
SLCO4C1
SLCO5A1
SLCO6A1
SLF1
SLFN11
SLFN12
SLFN12L
SLFN13
SLFN14
SLFN5
SLFNL1
SLIRP
SLIT1
SLIT1-AS1
SLIT2
SLIT3
SLITRK1

SLITRK2
SLITRK3
SLITRK4
SLITRK5
SLK
SLMAP
SLMO1
SLMO2
SLN
SLTM
SLU7
SLX1A
SLX1B
SLX4
SLX4IP
SMAD1
SMAD1-AS2
SMAD2
SMAD3
SMAD4
SMAD5
SMAD5-AS1
SMAD6
SMAD7
SMAD9
SMAGP
SMAP1
SMAP2
SMARCA1
SMARCA2
SMARCA4
SMARCA5
SMARCA5-AS1
SMARCAD1
SMARCAL1
SMARCB1
SMARCC1
SMARCC2
SMARCD1
SMARCD2
SMARCD3
SMARCE1
SMC1A
SMC1B
SMC2
SMC2-AS1
SMC3
SMC4
SMC5
SMC5-AS1

SMC6
SMCHD1
SMCO2
SMCO4
SMCP
SMCR5
SMCR7
SMCR7L
SMCR8
SMEK1
SMG1
SMG1P2
SMG1P5
SMG5
SMG6
SMG7
SMG9
SMIM1
SMIM10
SMIM11
SMIM12
SMIM14
SMIM15
SMIM17
SMIM18
SMIM19
SMIM2-AS1
SMIM21
SMIM23
SMIM4
SMIM5
SMIM7
SMIM8
SMIM9
SMN1
SMN2
SMNDC1
SMO
SMOC1
SMOC2
SMOX
SMPD1
SMPD2
SMPD3
SMPD4
SMPDL3A
SMPDL3B
SMPX
SMS
SMTN

SMTNL1
SMTNL2
SMU1
SMUG1
SMURF1
SMURF2
SMYD1
SMYD2
SMYD3
SMYD4
SMYD5
SNAI1
SNAI2
SNAI3
SNAP23
SNAP25
SNAP25-AS1
SNAP29
SNAP47
SNAP91
SNAPC1
SNAPC2
SNAPC3
SNAPC4
SNAPC5
SNAPIN
SNAR-C4
SNAR-E
SNAR-F
SNAR-I
SNCA
SNCAIP
SNCB
SND1
SNED1
SNF8
SNHG11
SNHG12
SNHG15
SNHG17
SNHG18
SNHG22
SNHG3
SNHG3-RCC1
SNHG4
SNHG5
SNHG7
SNHG8
SNHG9
SNIP1

SNN
SNORA10
SNORA11B
SNORA13
SNORA14B
SNORA15
SNORA16A
SNORA19
SNORA24
SNORA25
SNORA26
SNORA27
SNORA30
SNORA32
SNORA36C
SNORA38
SNORA4
SNORA42
SNORA47
SNORA49
SNORA52
SNORA54
SNORA6
SNORA60
SNORA63
SNORA70E
SNORA70F
SNORA71E
SNORA72
SNORA73B
SNORA74B
SNORA76
SNORA80E
SNORA9
SNORD103A
SNORD109A
SNORD11
SNORD113-1
SNORD113-4
SNORD113-5
SNORD113-6
SNORD114-1
SNORD114-10
SNORD114-12
SNORD114-16
SNORD114-18
SNORD114-20
SNORD114-22
SNORD114-23
SNORD114-25

SNORD114-26
SNORD114-29
SNORD114-31
SNORD114-4
SNORD114-5
SNORD115-1
SNORD115-12
SNORD115-13
SNORD115-14
SNORD115-15
SNORD115-16
SNORD115-19
SNORD115-2
SNORD115-21
SNORD115-29
SNORD115-31
SNORD115-33
SNORD115-37
SNORD115-39
SNORD115-40
SNORD115-42
SNORD115-7
SNORD115-8
SNORD115-9
SNORD116-1
SNORD116-10
SNORD116-12
SNORD116-14
SNORD116-18
SNORD116-24
SNORD116-25
SNORD116-4
SNORD126
SNORD12B
SNORD15A
SNORD16
SNORD17
SNORD18C
SNORD1C
SNORD21
SNORD22
SNORD24
SNORD26
SNORD27
SNORD30
SNORD32B
SNORD36C
SNORD42B
SNORD43
SNORD45C

SNORD48
SNORD50B
SNORD54
SNORD55
SNORD56
SNORD59A
SNORD60
SNORD62B
SNORD63
SNORD7
SNORD71
SNORD72
SNORD74
SNORD81
SNORD83A
SNORD83B
SNORD87
SNORD9
SNORD92
SNORD94
SNPH
SNRK
SNRK-AS1
SNRNP200
SNRNP25
SNRNP27
SNRNP35
SNRNP40
SNRNP48
SNRNP70
SNRPA1
SNRPB
SNRPB2
SNRPC
SNRPD3
SNRPE
SNRPF
SNRPG
SNRPN
SNTA1
SNTB1
SNTB2
SNTG1
SNTG2
SNTN
SNU13
SNUPN
SNW1
SNX1
SNX10

SNX11
SNX12
SNX13
SNX14
SNX15
SNX16
SNX17
SNX18
SNX19
SNX2
SNX20
SNX21
SNX22
SNX24
SNX25
SNX26
SNX27
SNX29
SNX29P1
SNX29P2
SNX3
SNX30
SNX31
SNX32
SNX33
SNX4
SNX5
SNX6
SNX7
SNX8
SNX9
SOAT1
SOAT2
SOBP
SOCS1
SOCS2
SOCS2-AS1
SOCS3
SOCS4
SOCS5
SOCS6
SOCS7
SOD1
SOD2
SOD3
SOGA1
SOGA3
SOHLH1
SOHLH2
SOLH

SON
SORBS1
SORBS2
SORBS3
SORCS1
SORCS2
SORCS3
SORD
SORL1
SORT1
SOS1
SOS2
SOST
SOSTDC1
SOWAHA
SOWAHB
SOX1
SOX10
SOX11
SOX12
SOX13
SOX14
SOX15
SOX17
SOX18
SOX2-OT
SOX21
SOX2OT
SOX3
SOX30
SOX4
SOX5
SOX6
SOX7
SOX8
SOX9
SOX9-AS1
SP1
SP100
SP110
SP140
SP140L
SP2
SP2-AS1
SP3
SP4
SP5
SP6
SP7
SP8

SP9
SPACA1
SPACA3
SPACA4
SPACA5
SPACA6P
SPACA6P-AS
SPACA7
SPAG1
SPAG11B
SPAG16
SPAG17
SPAG4
SPAG4L
SPAG5
SPAG6
SPAG8
SPAG9
SPAM1
SPARC
SPARCL1
SPAST
SPATA1
SPATA13
SPATA13-AS1
SPATA16
SPATA17
SPATA18
SPATA2
SPATA20
SPATA21
SPATA22
SPATA24
SPATA2L
SPATA3
SPATA3-AS1
SPATA33
SPATA4
SPATA5
SPATA5L1
SPATA6
SPATA6L
SPATA7
SPATA8
SPATA8-AS1
SPATA9
SPATC1
SPATC1L
SPATS1
SPATS2

SPATS2L
SPC24
SPC25
SPCS2
SPCS3
SPDEF
SPDL1
SPDYA
SPDYC
SPDYE1
SPDYE4
SPDYE6
SPECC1
SPECC1L
SPECC1L-ADORA2A
SPEF1
SPEF2
SPEG
SPEM1
SPEN
SPERT
SPESP1
SPG11
SPG20
SPG21
SPG7
SPHK1
SPHK2
SPHKAP
SPI1
SPIB
SPIC
SPICE1
SPIDR
SPIN1
SPIN2A
SPIN2B
SPIN3
SPIN4
SPINK1
SPINK2
SPINK5
SPINK5L2
SPINK5L3
SPINK7
SPINK8
SPINK9
SPINLW1
SPINT1
SPINT2

SPINT3
SPINT4
SPIRE1
SPIRE2
SPN
SPNS1
SPNS2
SPNS3
SPO11
SPOCD1
SPOCK1
SPOCK2
SPOCK3
SPON1
SPON2
SPOP
SPOPL
SPP1
SPPL2A
SPPL2B
SPPL2C
SPPL3
SPR
SPRED2
SPRED3
SPRN
SPRR1B
SPRR2A
SPRR2B
SPRR2C
SPRR2F
SPRY1
SPRY2
SPRY3
SPRY4
SPRYD3
SPRYD4
SPRYD5
SPRYD7
SPSB1
SPSB2
SPSB3
SPSB4
SPTA1
SPTAN1
SPTB
SPTBN1
SPTBN2
SPTBN4
SPTBN5

SPTLC1
SPTLC2
SPTLC3
SPTSSA
SPTY2D1
SPTY2D1-AS1
SPX
SPZ1
SQLE
SQRDL
SQSTM1
SRA1
SRBD1
SRC
SRCAP
SRCIN1
SRCRB4D
SRD5A1
SRD5A2
SRD5A3
SRD5A3-AS1
SREBF1
SREBF2
SREK1
SREK1IP1
SRF
SRFBP1
SRGAP1
SRGAP2
SRGAP2C
SRGAP3
SRGN
SRI
SRL
SRM
SRMS
SRP14
SRP14-AS1
SRP19
SRP54
SRP68
SRP72
SRP9
SRPK1
SRPK2
SRPK3
SRPR
SRPRB
SRPX
SRPX2

SRR
SRRM1
SRRM2
SRRM2-AS1
SRRM3
SRRM4
SRRT
SRSF10
SRSF11
SRSF12
SRSF2
SRSF5
SRSF7
SRSF8
SRSF9
SRXN1
SS18
SS18L1
SS18L2
SSB
SSBP1
SSBP2
SSBP3
SSBP3-AS1
SSBP4
SSC4D
SSC5D
SSFA2
SSH1
SSH2
SSH3
SSPN
SSPO
SSR1
SSR2
SSR3
SSR4
SSRP1
SSSCA1
SST
SSTR1
SSTR2
SSTR3
SSTR5
SSTR5-AS1
SSU72
SSUH2
SSX1
SSX2IP
SSX3

SSX5
SSX6
SSX7
ST13
ST14
ST18
ST20
ST20-MTHFS
ST3GAL1
ST3GAL2
ST3GAL3
ST3GAL4
ST3GAL5
ST3GAL5-AS1
ST3GAL6
ST5
ST6GAL1
ST6GAL2
ST6GALNAC2
ST6GALNAC3
ST6GALNAC4
ST6GALNAC5
ST6GALNAC6
ST7
ST7-AS2
ST7L
ST7OT2
ST7OT4
ST8SIA1
ST8SIA2
ST8SIA3
ST8SIA4
ST8SIA5
ST8SIA6
STAB1
STAB2
STAC
STAC2
STAC3
STAG1
STAG2
STAG3
STAG3L4
STAG3L5P-PVRIG2P-PILRB
STAM
STAM-AS1
STAM2
STAMBP
STAMBPL1
STAP1

STAP2
STAR
STARD10
STARD13
STARD13-AS
STARD3
STARD3NL
STARD4-AS1
STARD5
STARD6
STARD7
STARD8
STARD9
STAT1
STAT2
STAT3
STAT4
STAT5A
STAT5B
STAT6
STATH
STAU1
STAU2
STAU2-AS1
STBD1
STC1
STC2
STEAP1
STEAP1B
STEAP2
STEAP2-AS1
STEAP3
STEAP3-AS1
STEAP4
STIL
STIM1
STIM2
STIP1
STK10
STK11
STK11IP
STK16
STK17A
STK17B
STK19
STK24
STK25
STK3
STK31
STK32A

STK32B
STK32C
STK33
STK35
STK36
STK38
STK38L
STK39
STK4
STK4-AS1
STK40
STKLD1
STMN1
STMN2
STMN3
STMN4
STMND1
STOM
STOML1
STOML3
STON1-GTF2A1L
STON2
STOX1
STOX2
STPG1
STPG2
STPG2-AS1
STRA13
STRA6
STRA8
STRADA
STRADB
STRAP
STRBP
STRC
STRIP1
STRIP2
STRN
STRN3
STRN4
STS
STT3A
STT3B
STUB1
STX10
STX11
STX12
STX16
STX16-NPEPL1
STX17

STX18
STX18-AS1
STX1A
STX1B
STX2
STX3
STX4
STX5
STX6
STX7
STX8
STXBP1
STXBP2
STXBP4
STXBP5
STXBP5-AS1
STXBP5L
STXBP6
STYK1
STYX
STYXL1
SUB1
SUCLA2
SUCLG1
SUCLG2
SUCLG2-AS1
SUCO
SUDS3
SUFU
SUGCT
SUGP1
SUGP2
SUGT1
SUGT1L1
SUGT1P1
SULF1
SULF2
SULT1A1
SULT1A2
SULT1B1
SULT1C2
SULT1C2P1
SULT1C3
SULT1C4
SULT2A1
SULT2B1
SULT4A1
SUMF1
SUMF2
SUMO1

SUMO2
SUMO3
SUN1
SUN2
SUN3
SUN5
SUNC1
SUOX
SUPT16H
SUPT20H
SUPT3H
SUPT4H1
SUPT5H
SUPT6H
SUPT7L
SUPV3L1
SURF1
SURF2
SURF4
SURF6
SUSD1
SUSD2
SUSD4
SUSD5
SUSD6
SUV39H1
SUV39H2
SUV420H1
SUV420H2
SUZ12
SUZ12P
SUZ12P1
SV2A
SV2B
SV2C
SVBP
SVEP1
SVIL
SVIL-AS1
SVILP1
SVIP
SVOP
SVOPL
SWAP70
SWSAP1
SYAP1
SYBU
SYCE1
SYCE1L
SYCE2

SYCE3
SYCN
SYCP1
SYCP2L
SYCP3
SYDE1
SYDE2
SYK
SYMPK
SYN1
SYN2
SYN3
SYNC
SYNCRIP
SYNDIG1
SYNDIG1L
SYNE1
SYNE1-AS1
SYNE2
SYNE3
SYNGAP1
SYNGR1
SYNGR2
SYNGR3
SYNGR4
SYNJ1
SYNJ2
SYNJ2-IT1
SYNJ2BP-COX16
SYNM
SYNPO
SYNPO2
SYNPO2L
SYNPR
SYNRG
SYP
SYPL1
SYPL2
SYS1
SYS1-DBNDD2
SYT1
SYT10
SYT11
SYT12
SYT13
SYT14
SYT15
SYT16
SYT17
SYT2

SYT3
SYT4
SYT5
SYT6
SYT7
SYT8
SYT9
SYTL1
SYTL2
SYTL3
SYTL4
SYTL5
SYVN1
SZRD1
SZT2
T
TAAR2
TAAR3
TAAR6
TAAR8
TAAR9
TAB1
TAB2
TAB3
TAC1
TAC3
TAC4
TACC1
TACC2
TACC3
TACO1
TACR1
TACR2
TACR3
TACSTD2
TADA1
TADA2A
TADA2B
TAF1
TAF10
TAF12
TAF13
TAF1A
TAF1A-AS1
TAF1B
TAF1C
TAF1D
TAF1L
TAF2
TAF3

TAF4
TAF4B
TAF5
TAF5L
TAF6
TAF6L
TAF7
TAF7L
TAF8
TAF9
TAF9B
TAGAP
TAGLN
TAGLN2
TAGLN3
tAKR
TAL1
TALDO1
TAMM41
TANC1
TANC2
TANGO2
TANGO6
TANK
TAOK1
TAOK2
TAOK3
TAP1
TAP2
TAPBP
TAPBPL
TAPT1
TARBP1
TARBP2
TARDBP
TARID
TARM1
TARP
TARS
TARS2
TARSL2
TAS1R1
TAS1R2
TAS1R3
TAS2R1
TAS2R16
TAS2R3
TAS2R30
TAS2R31
TAS2R38

TAS2R39
TAS2R40
TAS2R42
TAS2R5
TAS2R9
TASP1
TAT-AS1
TATDN1
TATDN2
TATDN3
TAX1BP1
TAX1BP3
TAZ
TBATA
TBC1D1
TBC1D10A
TBC1D10B
TBC1D10C
TBC1D12
TBC1D13
TBC1D14
TBC1D15
TBC1D16
TBC1D17
TBC1D19
TBC1D2
TBC1D20
TBC1D21
TBC1D22A
TBC1D22A-AS1
TBC1D22B
TBC1D23
TBC1D24
TBC1D25
TBC1D26
TBC1D28
TBC1D29
TBC1D2B
TBC1D30
TBC1D31
TBC1D32
TBC1D3C
TBC1D3G
TBC1D3P2
TBC1D4
TBC1D5
TBC1D7
TBC1D8
TBC1D8B
TBC1D9

TBC1D9B
TBCA
TBCB
TBCC
TBCCD1
TBCD
TBCE
TBCEL
TBCK
TBK1
TBKBP1
TBL1X
TBL1XR1
TBL1Y
TBL3
TBP
TBPL1
TBPL2
TBR1
TBRG1
TBRG4
TBX1
TBX10
TBX15
TBX18
TBX19
TBX2
TBX20
TBX21
TBX22
TBX3
TBX4
TBX5
TBX6
TBXA2R
TBXAS1
TC2N
TCAF1
TCAF2
TCAM1
TCAP
TCEA1
TCEA2
TCEA3
TCEAL1
TCEAL2
TCEAL4
TCEAL5
TCEAL7
TCEAL8

TCEANC
TCEANC2
TCEB1
TCEB2
TCEB3
TCEB3-AS1
TCERG1
TCERG1L
TCERG1L-AS1
TCF12
TCF15
TCF19
TCF20
TCF21
TCF23
TCF24
TCF25
TCF3
TCF4
TCF7
TCF7L1
TCF7L2
TCFL5
TCHH
TCHHL1
TCHP
TCIRG1
TCL1A
TCL1B
TCL6
TCN1
TCN2
TCOF1
TCP1
TCP10
TCP10L
TCP10L2
TCP11
TCP11L1
TCP11L2
TCTE1
TCTEX1D1
TCTEX1D2
TCTEX1D4
TCTN1
TCTN2
TCTN3
TDG
TDGF1
TDH

TDO2
TDP1
TDRD1
TDRD10
TDRD12
TDRD3
TDRD5
TDRD6
TDRD7
TDRD9
TDRG1
TDRKH
TDRP
TEAD1
TEAD2
TEAD3
TEAD4
TEC
TECPR1
TECPR2
TECR
TECRL
TECTA
TECTB
TEDDM1
TEF
TEK
TEKT1
TEKT2
TEKT3
TEKT4
TEKT5
TELO2
TEN1
TENC1
TENM1
TENM2
TENM3
TENM4
TEP1
TEPP
TERC
TERF1
TERF2
TERT
TES
TESC
TESK1
TESK2
TESPA1

TESSP1
TET1
TET2
TET2-AS1
TET3
TEX10
TEX101
TEX11
TEX12
TEX13A
TEX13B
TEX14
TEX15
TEX19
TEX2
TEX22
TEX26
TEX261
TEX264
TEX29
TEX30
TEX33
TEX41
TF
TFAP2A
TFAP2B
TFAP2C
TFAP2D
TFAP2E
TFAP4
TFB1M
TFCP2
TFCP2L1
TFDP1
TFDP2
TFDP3
TFE3
TFEB
TFEC
TFF1
TFF2
TFF3
TFG
TFIP11
TFPI
TFPI2
TFPT
TFR2
TFRC
TG

TGDS
TGFA
TGFA-IT1
TGFB1
TGFB1I1
TGFB2
TGFB3
TGFBI
TGFBR2
TGFBR3
TGFBR3L
TGFBRAP1
TGIF1
TGIF2
TGIF2LX
TGIF2LY
TGM1
TGM2
TGM3
TGM4
TGM5
TGM6
TGM7
TGOLN2
TGS1
TH
TH1L
TH2LCRR
THADA
THAP1
THAP10
THAP11
THAP2
THAP3
THAP4
THAP5
THAP7
THAP7-AS1
THAP8
THAP9
THAP9-AS1
THBD
THBS1
THBS2
THBS3
THBS4
THCAT155
THCAT158
THEG
THEG5

THEGL
THEM4
THEM5
THEM6
THEMIS
THEMIS2
THG1L
THNSL1
THNSL2
THOC2
THOC5
THOC6
THOC7
THOC7-AS1
THOP1
THRA
THRA1/BTR
THRAP3
THRB
THRB-AS1
THRSP
THSD1
THSD4
THSD4-AS1
THSD7A
THSD7B
THTPA
THUMPD1
THUMPD2
THUMPD3
THUMPD3-AS1
THY1
THYN1
TIA1
TIAF1
TIAL1
TIAM1
TIAM2
TICAM1
TICAM2
TICRR
TIE1
TIFA
TIFAB
TIGAR
TIGD1
TIGD2
TIGD3
TIGD4
TIGD6

TIGD7
TIGIT
TIMD4
TIMELESS
TIMM10
TIMM10B
TIMM13
TIMM17B
TIMM22
TIMM44
TIMM50
TIMM8B
TIMM9
TIMMDC1
TIMP1
TIMP2
TIMP3
TIMP4
TINAG
TINAGL1
TINCR
TINF2
TIPARP
TIPARP-AS1
TIPRL
TIRAP
TJAP1
TJP1
TJP2
TJP3
TK1
TK2
TKFC
TKT
TKTL1
TLCD1
TLCD2
TLE1
TLE2
TLE3
TLE4
TLE6
TLK1
TLK2
TLL1
TLL2
TLN1
TLN2
TLR1
TLR2

TLR3
TLR4
TLR5
TLR6
TLR7
TLR8
TLR9
TLX1
TLX1NB
TLX2
TLX3
TM2D1
TM2D3
TM4SF1
TM4SF18
TM4SF19
TM4SF19-AS1
TM4SF20
TM4SF4
TM4SF5
TM6SF1
TM6SF2
TM7SF2
TM7SF3
TM7SF4
TM9SF1
TM9SF2
TM9SF3
TM9SF4
TMA16
TMBIM1
TMBIM4
TMBIM6
TMC1
TMC2
TMC3
TMC3-AS1
TMC4
TMC5
TMC6
TMC7
TMC8
TMCC1
TMCC2
TMCC3
TMCO1
TMCO2
TMCO3
TMCO4
TMCO5A

TMCO5B
TMCO6
TMCO7
TMED1
TMED10
TMED11P
TMED2
TMED3
TMED4
TMED5
TMED6
TMED7
TMED7-TICAM2
TMED8
TMED9
TMEFF2
TMEM100
TMEM101
TMEM104
TMEM105
TMEM106B
TMEM106C
TMEM107
TMEM108
TMEM109
TMEM11
TMEM110
TMEM110-MUSTN1
TMEM111
TMEM114
TMEM115
TMEM116
TMEM117
TMEM119
TMEM120A
TMEM120B
TMEM121
TMEM123
TMEM125
TMEM126A
TMEM126B
TMEM127
TMEM128
TMEM129
TMEM130
TMEM131
TMEM132A
TMEM132B
TMEM132C
TMEM132D

TMEM132E
TMEM133
TMEM134
TMEM135
TMEM136
TMEM138
TMEM139
TMEM140
TMEM141
TMEM143
TMEM144
TMEM145
TMEM146
TMEM147
TMEM14A
TMEM14B
TMEM14C
TMEM150A
TMEM150B
TMEM150C
TMEM151A
TMEM151B
TMEM154
TMEM155
TMEM158
TMEM159
TMEM161A
TMEM161B
TMEM161B-AS1
TMEM163
TMEM164
TMEM165
TMEM167A
TMEM167B
TMEM168
TMEM169
TMEM17
TMEM170A
TMEM170B
TMEM171
TMEM173
TMEM174
TMEM175
TMEM176B
TMEM177
TMEM178
TMEM178A
TMEM178B
TMEM179
TMEM179B

TMEM18
TMEM180
TMEM181
TMEM182
TMEM184A
TMEM184B
TMEM184C
TMEM185A
TMEM185B
TMEM186
TMEM187
TMEM188
TMEM189
TMEM189-UBE2V1
TMEM190
TMEM191A
TMEM191B
TMEM192
TMEM194A
TMEM194B
TMEM196
TMEM198B
TMEM2
TMEM20
TMEM200A
TMEM200B
TMEM200C
TMEM201
TMEM202
TMEM203
TMEM204
TMEM206
TMEM208
TMEM209
TMEM210
TMEM211
TMEM212
TMEM212-AS1
TMEM213
TMEM214
TMEM215
TMEM216
TMEM217
TMEM218
TMEM219
TMEM22
TMEM220
TMEM220-AS1
TMEM221
TMEM222

TMEM229A
TMEM229B
TMEM231
TMEM232
TMEM233
TMEM234
TMEM235
TMEM237
TMEM239
TMEM240
TMEM241
TMEM242
TMEM243
TMEM245
TMEM248
TMEM251
TMEM252
TMEM254-AS1
TMEM255A
TMEM256
TMEM258
TMEM26
TMEM260
TMEM261
TMEM262
TMEM263
TMEM30A
TMEM30C
TMEM31
TMEM33
TMEM35
TMEM37
TMEM38A
TMEM38B
TMEM39A
TMEM39B
TMEM40
TMEM41A
TMEM41B
TMEM43
TMEM44
TMEM44-AS1
TMEM45A
TMEM45B
TMEM47
TMEM48
TMEM49
TMEM5
TMEM50A
TMEM50B

TMEM51
TMEM51-AS1
TMEM52
TMEM53
TMEM54
TMEM55A
TMEM55B
TMEM56
TMEM57
TMEM59
TMEM59L
TMEM61
TMEM62
TMEM63A
TMEM63B
TMEM63C
TMEM64
TMEM65
TMEM66
TMEM67
TMEM68
TMEM69
TMEM70
TMEM71
TMEM72
TMEM72-AS1
TMEM74
TMEM75
TMEM79
TMEM80
TMEM82
TMEM85
TMEM86A
TMEM87B
TMEM88
TMEM88B
TMEM8A
TMEM8B
TMEM9
TMEM90A
TMEM90B
TMEM91
TMEM92
TMEM93
TMEM95
TMEM97
TMEM98
TMEM99
TMEM9B
TMF1

TMIE
TMIGD1
TMIGD2
TMIGD3
TMOD1
TMOD2
TMOD3
TMOD4
TMPO-AS1
TMPPE
TMPRSS11A
TMPRSS11D
TMPRSS11E
TMPRSS11F
TMPRSS12
TMPRSS13
TMPRSS15
TMPRSS2
TMPRSS3
TMPRSS4
TMPRSS4-AS1
TMPRSS5
TMPRSS6
TMPRSS7
TMPRSS8
TMPRSS9
TMSB15A
TMSB15B
TMSL3
TMTC1
TMTC2
TMTC3
TMTC4
TMUB2
TMX1
TMX2
TMX2-CTNND1
TMX3
TMX4
TNC
TNF
TNFAIP1
TNFAIP2
TNFAIP3
TNFAIP6
TNFAIP8
TNFAIP8L1
TNFAIP8L2
TNFAIP8L2-SCNM1
TNFAIP8L3

TNFRSF10A
TNFRSF10B
TNFRSF10C
TNFRSF10D
TNFRSF11A
TNFRSF11B
TNFRSF12A
TNFRSF13B
TNFRSF13C
TNFRSF17
TNFRSF18
TNFRSF19
TNFRSF1A
TNFRSF1B
TNFRSF21
TNFRSF25
TNFRSF4
TNFRSF8
TNFRSF9
TNFSF10
TNFSF11
TNFSF12
TNFSF13B
TNFSF14
TNFSF15
TNFSF18
TNFSF8
TNFSF9
TNIK
TNIP1
TNIP2
TNIP3
TNK1
TNK2
TNKS
TNKS1BP1
TNKS2
TNMD
TNN
TNNC1
TNNC2
TNNI2
TNNI3
TNNI3K
TNNT1
TNNT2
TNNT3
TNP1
TNPO1
TNPO2

TNR
TNRC18
TNRC4
TNRC6A
TNRC6B
TNRC6C
TNS1
TNS2
TNS3
TNS4
TNXB
TOB1
TOB2
TOLLIP
TOM1
TOM1L1
TOM1L2
TOMM20
TOMM20L
TOMM22
TOMM34
TOMM40
TOMM40L
TOMM5
TOMM70A
TONSL
TOP1
TOP1MT
TOP1P2
TOP2A
TOP2B
TOP3A
TOP3B
TOPAZ1
TOPBP1
TOPORS
TOR1A
TOR1AIP1
TOR1AIP2
TOR2A
TOR3A
TOR4A
TOX
TOX2
TOX3
TOX4
TP53
TP53AIP1
TP53BP1
TP53BP2

TP53I11
TP53I13
TP53I3
TP53INP1
TP53INP2
TP53RK
TP53TG1
TP53TG3D
TP53TG5
TP63
TP73
TP73-AS1
TPBG
TPBGL
TPCN1
TPCN2
TPD52
TPD52L1
TPD52L2
TPD52L3
TPGS1
TPH1
TPH2
TPK1
TPM1
TPM2
TPM3
TPM4
TPO
TPP1
TPP2
TPPP
TPPP2
TPPP3
TPR
TPRA1
TPRG1
TPRG1L
TPRX1
TPRXL
TPSAB1
TPSB2
TPSD1
TPSG1
TPST1
TPST2
TPT1
TPT1-AS1
TPTE
TPTE2

TPTE2P1
TPTE2P3
TPTEP1
TPX2
TRA2A
TRA2B
TRABD
TRABD2A
TRABD2B
TRADD
TRAF1
TRAF2
TRAF3
TRAF3IP1
TRAF3IP2
TRAF3IP2-AS1
TRAF3IP3
TRAF4
TRAF5
TRAF6
TRAF7
TRAFD1
TRAIP
TRAK1
TRAK2
TRAM1
TRAM1L1
TRAM2
TRANK1
TRAP1
TRAPPC1
TRAPPC10
TRAPPC11
TRAPPC12
TRAPPC13
TRAPPC2
TRAPPC2L
TRAPPC3
TRAPPC3L
TRAPPC4
TRAPPC5
TRAPPC6A
TRAPPC6B
TRAPPC9
TRAT1
TRDMT1
TRDN
TREH
TREM1
TREM2

TREML1
TREML2
TREML2P
TRERF1
TRERNA1
TREX1
TREX2
TRG-AS1
TRH
TRHDE
TRHR
TRIAP1
TRIB1
TRIB2
TRIB3
TRIL
TRIM10
TRIM11
TRIM13
TRIM14
TRIM15
TRIM16
TRIM16L
TRIM17
TRIM2
TRIM21
TRIM22
TRIM23
TRIM24
TRIM25
TRIM26
TRIM27
TRIM28
TRIM29
TRIM3
TRIM31
TRIM32
TRIM33
TRIM34
TRIM36
TRIM37
TRIM38
TRIM39
TRIM4
TRIM40
TRIM41
TRIM42
TRIM44
TRIM45
TRIM46

TRIM47
TRIM49D2
TRIM5
TRIM50
TRIM51HP
TRIM52
TRIM53
TRIM54
TRIM55
TRIM56
TRIM58
TRIM59
TRIM6
TRIM6-TRIM34
TRIM60
TRIM61
TRIM62
TRIM63
TRIM64C
TRIM65
TRIM66
TRIM67
TRIM68
TRIM69
TRIM7
TRIM71
TRIM72
TRIM8
TRIM9
TRIML1
TRIO
TRIOBP
TRIP10
TRIP11
TRIP12
TRIP13
TRIP4
TRIP6
TRIQK
TRIT1
TRMT1
TRMT10A
TRMT10B
TRMT11
TRMT12
TRMT1L
TRMT2A
TRMT2B
TRMT44
TRMT5

TRMT61A
TRMT61B
TRMU
TRNAU1AP
TRNP1
TRNT1
TRO
TROAP
TROVE2
TRPA1
TRPC1
TRPC3
TRPC4
TRPC4AP
TRPC5
TRPC6
TRPC7
TRPM1
TRPM2
TRPM3
TRPM4
TRPM5
TRPM6
TRPM7
TRPM8
TRPS1
TRPT1
TRPV1
TRPV2
TRPV3
TRPV4
TRPV5
TRPV6
TRRAP
TRUB1
TRY2P
TRYX3
TSC1
TSC2
TSC22D1
TSC22D2
TSC22D3
TSC22D4
TSEN15
TSEN2
TSEN34
TSEN54
TSFM
TSG101
TSGA10

TSGA10IP
TSGA13
TSGA14
TSHB
TSHR
TSHZ1
TSHZ2
TSHZ3
TSIX
TSKS
TSKU
TSLP
TSN
TSNARE1
TSNAX
TSNAX-DISC1
TSNAXIP1
TSPAN1
TSPAN10
TSPAN11
TSPAN12
TSPAN14
TSPAN15
TSPAN16
TSPAN17
TSPAN18
TSPAN2
TSPAN3
TSPAN31
TSPAN32
TSPAN33
TSPAN4
TSPAN5
TSPAN6
TSPAN7
TSPAN8
TSPAN9
TSPEAR
TSPEAR-AS1
TSPO
TSPY3
TSPY4
TSPYL1
TSPYL2
TSPYL3
TSPYL4
TSPYL5
TSR1
TSR2
TSR3

TSSC1
TSSC2
TSSC4
TSSK1B
TSSK2
TSSK3
TSSK6
TST
TSTA3
TSTD1
TSTD2
TSTD3
TTBK1
TTBK2
TTC1
TTC12
TTC13
TTC14
TTC15
TTC16
TTC17
TTC18
TTC19
TTC21A
TTC21B
TTC22
TTC23
TTC23L
TTC24
TTC25
TTC26
TTC27
TTC28
TTC28-AS1
TTC29
TTC3
TTC30A
TTC31
TTC32
TTC33
TTC34
TTC35
TTC36
TTC37
TTC38
TTC39A
TTC39A-AS1
TTC39B
TTC39C
TTC39C-AS1

TTC4
TTC41P
TTC5
TTC6
TTC7A
TTC7B
TTC8
TTC9
TTC9B
TTC9C
TTF1
TTF2
TTI1
TTI2
TTK
TTL
TTLL1
TTLL10
TTLL11
TTLL12
TTLL2
TTLL3
TTLL4
TTLL5
TTLL6
TTLL7
TTLL8
TTLL9
TTN
TTN-AS1
TTPA
TTPAL
TTR
TTRAP
TTTY10
TTTY14
TTTY20
TTYH1
TTYH2
TTYH3
TUB
TUBA1A
TUBA1B
TUBA1C
TUBA3C
TUBA3D
TUBA4A
TUBA4B
TUBA8
TUBAL3

TUBB
TUBB1
TUBB2B
TUBB2C
TUBB3
TUBB4
TUBB4A
TUBB4B
TUBB4Q
TUBB6
TUBB8
TUBBP5
TUBD1
TUBE1
TUBG2
TUBGCP2
TUBGCP3
TUBGCP4
TUBGCP6
TUFM
TUFT1
TUG1
TULP1
TULP2
TULP3
TULP4
TUNAR
TUSC2
TUSC3
TUSC4
TUSC5
TUT1
TVP23A
TVP23C
TVP23C-CDRT4
TWF1
TWF2
TWIST1
TWIST2
TWISTNB
TWSG1
TXK
TXLNA
TXLNB
TXLNG
TXLNGY
TXN2
TXNDC11
TXNDC12
TXNDC15

TXNDC16
TXNDC17
TXNDC2
TXNDC3
TXNDC5
TXNDC6
TXNDC9
TXNIP
TXNL1
TXNL4A
TXNL4B
TXNRD1
TXNRD2
TXNRD3
TXNRD3IT1
TXNRD3NB
TYK2
TYMP
TYMS
TYMSOS
TYR
TYRO3
TYROBP
TYRP1
TYSND1
TYW1
TYW1B
TYW3
U2AF1
U2AF1L4
U2AF2
U2SURP
UACA
UAP1
UAP1L1
UBA1
UBA2
UBA3
UBA5
UBA6
UBA7
UBAC1
UBAC2
UBAP1
UBAP1L
UBAP2
UBAP2L
UBASH3A
UBASH3B
UBB

UBC
UBD
UBE2A
UBE2B
UBE2C
UBE2CBP
UBE2D1
UBE2D2
UBE2D3
UBE2D4
UBE2DNL
UBE2E1
UBE2E1-AS1
UBE2E2
UBE2E3
UBE2F
UBE2G1
UBE2G2
UBE2H
UBE2I
UBE2J1
UBE2J2
UBE2K
UBE2L3
UBE2L6
UBE2M
UBE2MP1
UBE2N
UBE2O
UBE2Q1
UBE2Q2
UBE2Q2P1
UBE2QL1
UBE2QP1
UBE2R2
UBE2S
UBE2T
UBE2U
UBE2V1
UBE2V2
UBE2W
UBE2Z
UBE3A
UBE3B
UBE3C
UBE3D
UBE4A
UBE4B
UBFD1
UBIAD1

UBL3
UBL4A
UBL4B
UBL5
UBL7
UBLCP1
UBN1
UBN2
UBOX5
UBOX5-AS1
UBP1
UBQLN1
UBQLN3
UBQLN4
UBQLNL
UBR1
UBR2
UBR3
UBR4
UBR5
UBR7
UBTD1
UBTD2
UBTF
UBXN1
UBXN10
UBXN10-AS1
UBXN11
UBXN2A
UBXN2B
UBXN4
UBXN6
UBXN7
UBXN8
UCHL1
UCHL5
UCK1
UCK2
UCKL1
UCKL1AS
UCMA
UCN
UCN2
UCN3
UCP1
UCP2
UCP3
UCRC
UEVLD
UFC1

UFD1L
UFL1
UFM1
UFSP1
UFSP2
UG0898H09
UGCG
UGDH
UGGT1
UGGT2
UGP2
UGT1A1
UGT1A10
UGT1A5
UGT1A6
UGT1A7
UGT1A8
UGT1A9
UGT2A1
UGT2B11
UGT2B15
UGT2B7
UGT3A1
UGT3A2
UGT8
UHRF1
UHRF1BP1
UHRF1BP1L
UHRF2
UIMC1
ULBP1
ULBP2
ULBP3
ULK1
ULK2
ULK3
ULK4
ULK4P3
UMAD1
UMOD
UMODL1
UMPS
UNC119
UNC119B
UNC13A
UNC13B
UNC13C
UNC13D
UNC45A
UNC45B

UNC50
UNC5A
UNC5B
UNC5C
UNC5CL
UNC5D
UNC79
UNC80
UNC84A
UNC84B
UNC93A
UNC93B1
UNCX
UNG
UNK
UNKL
UNQ6494
UOX
UPB1
UPF1
UPF2
UPF3A
UPF3B
UPK1A
UPK1A-AS1
UPK1B
UPK2
UPK3A
UPK3B
UPP1
UPP2
UPRT
UQCC
UQCC1
UQCC2
UQCR
UQCR11
UQCRB
UQCRC1
UQCRC2
UQCRFS1
UQCRH
UQCRQ
URB1
URB2
URGCP
URGCP-MRPS24
URI1
URM1
UROC1

UROD
UROS
USB1
USE1
USF1
USF2
USH1C
USH2A
USHBP1
USO1
USP1
USP10
USP11
USP12
USP12-AS1
USP12-AS2
USP13
USP14
USP15
USP16
USP17L1
USP17L2
USP17L7
USP18
USP19
USP2
USP2-AS1
USP20
USP21
USP22
USP24
USP25
USP27X
USP28
USP29
USP3
USP3-AS1
USP30
USP31
USP32
USP32P1
USP33
USP34
USP35
USP36
USP37
USP38
USP39
USP4
USP40

USP42
USP43
USP44
USP45
USP46
USP46-AS1
USP47
USP48
USP49
USP5
USP50
USP51
USP53
USP54
USP6
USP6NL
USP7
USP8
USP9X
UST
UTAT33
UTF1
UTP11L
UTP14A
UTP15
UTP18
UTP20
UTP23
UTP3
UTP6
UTRN
UTS2
UTS2B
UTS2D
UTS2R
UTY
UVRAG
UVSSA
UXS1
UXT
VAC14
VAC14-AS1
VAMP1
VAMP2
VAMP3
VAMP4
VAMP5
VAMP8
VANGL1
VANGL2

VAPA
VAPB
VARS
VARS2
VASH1
VASH2
VASN
VASP
VAT1
VAT1L
VAV1
VAV2
VAV3
VAX1
VAX2
VBP1
VCAM1
VCAN
VCL
VCP
VCPIP1
VCX
VCX3A
VCX3B
VDAC1
VDAC2
VDAC3
VDR
VEGFA
VEGFB
VEGFC
VENTX
VENTXP1
VENTXP7
VEPH1
VEZF1
VEZT
VGF
VGLL1
VGLL2
VGLL3
VGLL4
VHL
VHLL
VIL1
VILL
VIM
VIMP
VIP
VIPR1

VIPR2
VIT
VKORC1
VKORC1L1
VLDLR
VLDLR-AS1
VMA21
VMAC
VMO1
VMP1
VN1R2
VNN1
VNN2
VNN3
VOPP1
VPRBP
VPREB1
VPREB3
VPS11
VPS13A-AS1
VPS13B
VPS13C
VPS13D
VPS16
VPS18
VPS24
VPS25
VPS26A
VPS26B
VPS28
VPS29
VPS33A
VPS33B
VPS36
VPS37A
VPS37B
VPS37C
VPS37D
VPS39
VPS41
VPS45
VPS4A
VPS4B
VPS50
VPS51
VPS52
VPS53
VPS54
VPS72
VPS8

VRK1
VRK2
VRK3
VRTN
VSIG1
VSIG10
VSIG10L
VSIG2
VSIG4
VSIG8
VSNL1
VSTM1
VSTM2A
VSTM2B
VSTM2L
VSTM4
VSX1
VSX2
VTA1
VTCN1
VTI1A
VTI1B
VTRNA1-3
VWA1
VWA2
VWA3A
VWA3B
VWA5A
VWA5B1
VWA5B2
VWA7
VWA8
VWA8-AS1
VWA9
VWC2
VWC2L
VWCE
VWDE
VWF
WAC
WAC-AS1
WAPAL
WARS
WARS2
WAS
WASF1
WASF2
WASF3
WASH1
WASH2P

WASH3P
WASH5P
WASL
WBP11
WBP1L
WBP2
WBP2NL
WBP4
WBP5
WBSCR16
WBSCR17
WBSCR22
WBSCR27
WDFY1
WDFY2
WDFY3
WDFY4
WDHD1
WDPCP
WDR1
WDR11
WDR11-AS1
WDR12
WDR13
WDR16
WDR17
WDR18
WDR19
WDR20
WDR24
WDR25
WDR26
WDR27
WDR3
WDR31
WDR33
WDR34
WDR35
WDR36
WDR37
WDR38
WDR4
WDR41
WDR43
WDR44
WDR45
WDR45B
WDR45L
WDR46
WDR47

WDR48
WDR49
WDR5
WDR51A
WDR51B
WDR52
WDR53
WDR54
WDR55
WDR59
WDR5B
WDR6
WDR60
WDR61
WDR62
WDR63
WDR64
WDR65
WDR66
WDR67
WDR69
WDR7
WDR70
WDR72
WDR73
WDR74
WDR75
WDR77
WDR78
WDR8
WDR81
WDR82
WDR83
WDR85
WDR86
WDR87
WDR88
WDR89
WDR90
WDR91
WDR92
WDR93
WDSUB1
WDTC1
WDYHV1
WEE1
WEE2-AS1
WFDC1
WFDC10B
WFDC11

WFDC12
WFDC13
WFDC2
WFDC3
WFDC6
WFDC8
WFDC9
WFIKKN1
WFIKKN2
WFS1
WHAMM
WHAMML2
WHAMMP2
WHSC1
WHSC1L1
WHSC2
WIBG
WIF1
WIPF1
WIPF2
WIPF3
WIPI1
WIPI2
WISP1
WISP2
WISP3
WIT1
WIZ
WLS
WNK1
WNK2
WNK3
WNK4
WNT1
WNT10A
WNT10B
WNT11
WNT16
WNT2
WNT2B
WNT3
WNT3A
WNT4
WNT5A
WNT5B
WNT6
WNT7A
WNT7B
WNT8A
WNT8B

WNT9A
WNT9B
WRAP53
WRAP73
WRB
WRN
WRNIP1
WSB1
WSB2
WSCD1
WSCD2
WSPAR
WT1
WT1-AS
WTAP
WTAPP1
WTH3DI
WTIP
WWC1
WWC2
WWC2-AS1
WWC2-AS2
WWC3
WWOX
WWP1
WWP2
WWTR1
WWTR1-AS1
XAB2
XACT
XAF1
XAGE3
XAGE5
XBP1
XCL1
XDH
XG
XIAP
XIRP1
XIRP2
XIRP2-AS1
XIST
XK
XKR4
XKR5
XKR6
XKR7
XKR8
XKR9
XKRX

XLOC_008559
XPA
XPC
XPNPEP1
XPNPEP2
XPNPEP3
XPO4
XPO5
XPO6
XPO7
XPOT
XPR1
XRCC1
XRCC2
XRCC3
XRCC4
XRCC5
XRCC6
XRCC6BP1
XRN1
XRN2
XRRA1
XXYLT1
XXYLT1-AS1
XYLB
XYLT1
XYLT2
YAE1D1
YAF2
YAP1
YARS
YARS2
YBEY
YBX1
YBX2
YBX3
YDJC
YEATS2
YEATS4
YES1
YIF1A
YIF1B
YIPF1
YIPF2
YIPF3
YIPF4
YIPF5
YIPF6
YIPF7
YJEFN3

YKT6
YLPM1
YOD1
YPEL1
YPEL2
YPEL3
YPEL4
YPEL5
YRDC
YSK4
YTHDC1
YTHDC2
YTHDF1
YTHDF2
YTHDF3
YWHAB
YWHAE
YWHAG
YWHAH
YWHAQ
YWHAZ
YY1
YY1AP1
ZACN
ZADH2
ZAK
ZAN
ZAP70
ZAR1
ZAR1L
ZBBX
ZBED3
ZBED3-AS1
ZBED4
ZBED5
ZBED5-AS1
ZBED6
ZBED6CL
ZBED8
ZBP1
ZBTB1
ZBTB10
ZBTB11
ZBTB11-AS1
ZBTB12
ZBTB14
ZBTB16
ZBTB17
ZBTB2
ZBTB20

ZBTB20-AS1
ZBTB20-AS3
ZBTB22
ZBTB24
ZBTB25
ZBTB26
ZBTB3
ZBTB32
ZBTB33
ZBTB34
ZBTB38
ZBTB39
ZBTB4
ZBTB40
ZBTB41
ZBTB42
ZBTB43
ZBTB44
ZBTB45
ZBTB46
ZBTB47
ZBTB48
ZBTB5
ZBTB6
ZBTB7A
ZBTB7B
ZBTB7C
ZBTB8A
ZBTB8B
ZBTB9
ZC2HC1B
ZC3H10
ZC3H11A
ZC3H12A
ZC3H12B
ZC3H12C
ZC3H12D
ZC3H13
ZC3H14
ZC3H15
ZC3H18
ZC3H3
ZC3H4
ZC3H6
ZC3H7A
ZC3H7B
ZC3H8
ZC3HAV1
ZC3HAV1L
ZC3HC1

ZC4H2
ZCCHC10
ZCCHC11
ZCCHC12
ZCCHC13
ZCCHC14
ZCCHC16
ZCCHC17
ZCCHC18
ZCCHC2
ZCCHC24
ZCCHC3
ZCCHC4
ZCCHC5
ZCCHC6
ZCCHC7
ZCCHC8
ZCCHC9
ZCWPW1
ZDBF2
ZDHHC1
ZDHHC11
ZDHHC12
ZDHHC13
ZDHHC14
ZDHHC15
ZDHHC16
ZDHHC17
ZDHHC18
ZDHHC19
ZDHHC2
ZDHHC20
ZDHHC21
ZDHHC22
ZDHHC23
ZDHHC24
ZDHHC3
ZDHHC4
ZDHHC5
ZDHHC6
ZDHHC7
ZDHHC8
ZDHHC8P1
ZDHHC9
ZEB1
ZEB2
ZEB2-AS1
ZER1
ZFAND1
ZFAND2A

ZFAND2B
ZFAND3
ZFAND4
ZFAND5
ZFAND6
ZFAT
ZFC3H1
ZFHX2
ZFHX3
ZFHX4
ZFHX4-AS1
ZFP1
ZFP106
ZFP112
ZFP14
ZFP161
ZFP2
ZFP28
ZFP3
ZFP30
ZFP36L1
ZFP36L2
ZFP37
ZFP41
ZFP42
ZFP57
ZFP62
ZFP64
ZFP69
ZFP69B
ZFP82
ZFP90
ZFP91
ZFP91-CNTF
ZFP92
ZFPM1
ZFPM2
ZFPM2-AS1
ZFR
ZFR2
ZFX
ZFY
ZFYVE1
ZFYVE16
ZFYVE19
ZFYVE20
ZFYVE21
ZFYVE26
ZFYVE27
ZFYVE28

ZFYVE9
ZG16B
ZGLP1
ZGPAT
ZHX1
ZHX1-C8orf76
ZHX2
ZHX3
ZIC1
ZIC2
ZIC3
ZIC4
ZIC5
ZIK1
ZIM2
ZKSCAN1
ZKSCAN2
ZKSCAN3
ZKSCAN4
ZKSCAN5
ZKSCAN8
ZMAT1
ZMAT2
ZMAT3
ZMAT4
ZMAT5
ZMIZ1
ZMIZ1-AS1
ZMIZ2
ZMPSTE24
ZMYM1
ZMYM2
ZMYM3
ZMYM4
ZMYM5
ZMYND10
ZMYND11
ZMYND12
ZMYND15
ZMYND19
ZMYND8
ZNF10
ZNF100
ZNF101
ZNF106
ZNF107
ZNF112
ZNF114
ZNF117
ZNF12

ZNF121
ZNF124
ZNF131
ZNF132
ZNF133
ZNF134
ZNF135
ZNF136
ZNF14
ZNF140
ZNF141
ZNF142
ZNF143
ZNF146
ZNF148
ZNF154
ZNF155
ZNF157
ZNF16
ZNF160
ZNF165
ZNF167
ZNF169
ZNF17
ZNF174
ZNF175
ZNF177
ZNF180
ZNF181
ZNF182
ZNF184
ZNF185
ZNF187
ZNF189
ZNF19
ZNF192
ZNF193
ZNF195
ZNF197
ZNF2
ZNF20
ZNF200
ZNF202
ZNF204P
ZNF205
ZNF205-AS1
ZNF207
ZNF208
ZNF211
ZNF212

ZNF213
ZNF214
ZNF217
ZNF219
ZNF22
ZNF221
ZNF223
ZNF224
ZNF225
ZNF226
ZNF227
ZNF229
ZNF23
ZNF230
ZNF232
ZNF233
ZNF235
ZNF236
ZNF238
ZNF239
ZNF24
ZNF248
ZNF25
ZNF250
ZNF251
ZNF252
ZNF252P
ZNF254
ZNF257
ZNF26
ZNF260
ZNF263
ZNF264
ZNF266
ZNF267
ZNF268
ZNF271P
ZNF273
ZNF274
ZNF275
ZNF276
ZNF277
ZNF28
ZNF280A
ZNF280B
ZNF280D
ZNF281
ZNF282
ZNF283
ZNF284

ZNF285
ZNF285A
ZNF286A
ZNF286B
ZNF287
ZNF292
ZNF295
ZNF296
ZNF3
ZNF302
ZNF304
ZNF311
ZNF317
ZNF318
ZNF319
ZNF32
ZNF32-AS3
ZNF321
ZNF322A
ZNF322B
ZNF323
ZNF324
ZNF324B
ZNF326
ZNF329
ZNF330
ZNF331
ZNF333
ZNF334
ZNF335
ZNF337
ZNF33A
ZNF33B
ZNF34
ZNF341
ZNF341-AS1
ZNF343
ZNF345
ZNF346
ZNF347
ZNF350
ZNF350-AS1
ZNF354A
ZNF354B
ZNF354C
ZNF358
ZNF362
ZNF365
ZNF366
ZNF367

ZNF37A
ZNF37B
ZNF382
ZNF384
ZNF385A
ZNF385B
ZNF385C
ZNF385D
ZNF391
ZNF394
ZNF395
ZNF396
ZNF397
ZNF397OS
ZNF398
ZNF407
ZNF408
ZNF41
ZNF410
ZNF414
ZNF415
ZNF416
ZNF417
ZNF418
ZNF419
ZNF420
ZNF423
ZNF425
ZNF426
ZNF428
ZNF429
ZNF43
ZNF431
ZNF432
ZNF433
ZNF436
ZNF436-AS1
ZNF438
ZNF439
ZNF44
ZNF440
ZNF441
ZNF442
ZNF443
ZNF444
ZNF445
ZNF446
ZNF449
ZNF45
ZNF451

ZNF454
ZNF460
ZNF461
ZNF462
ZNF467
ZNF469
ZNF470
ZNF471
ZNF473
ZNF474
ZNF479
ZNF48
ZNF480
ZNF483
ZNF484
ZNF485
ZNF486
ZNF487
ZNF488
ZNF490
ZNF491
ZNF492
ZNF496
ZNF497
ZNF498
ZNF500
ZNF501
ZNF502
ZNF503
ZNF503-AS2
ZNF506
ZNF507
ZNF509
ZNF510
ZNF511
ZNF512
ZNF512B
ZNF513
ZNF514
ZNF516
ZNF517
ZNF518A
ZNF518B
ZNF519
ZNF521
ZNF524
ZNF525
ZNF526
ZNF527
ZNF528

ZNF529
ZNF530
ZNF532
ZNF534
ZNF536
ZNF540
ZNF541
ZNF542
ZNF543
ZNF544
ZNF546
ZNF547
ZNF548
ZNF549
ZNF552
ZNF554
ZNF555
ZNF557
ZNF558
ZNF559
ZNF560
ZNF561
ZNF562
ZNF563
ZNF564
ZNF565
ZNF567
ZNF568
ZNF569
ZNF57
ZNF570
ZNF571
ZNF571-AS1
ZNF572
ZNF573
ZNF574
ZNF575
ZNF576
ZNF577
ZNF578
ZNF579
ZNF580
ZNF581
ZNF582
ZNF582-AS1
ZNF583
ZNF584
ZNF586
ZNF587
ZNF587B

ZNF589
ZNF592
ZNF593
ZNF594
ZNF596
ZNF597
ZNF598
ZNF599
ZNF600
ZNF605
ZNF606
ZNF607
ZNF608
ZNF609
ZNF610
ZNF611
ZNF613
ZNF614
ZNF615
ZNF616
ZNF618
ZNF619
ZNF620
ZNF621
ZNF622
ZNF623
ZNF624
ZNF625
ZNF625-ZNF20
ZNF626
ZNF627
ZNF628
ZNF629
ZNF630
ZNF638
ZNF639
ZNF641
ZNF644
ZNF646
ZNF648
ZNF649
ZNF652
ZNF653
ZNF654
ZNF655
ZNF660
ZNF662
ZNF664
ZNF664-FAM101A
ZNF665

ZNF667
ZNF667-AS1
ZNF668
ZNF669
ZNF671
ZNF672
ZNF673
ZNF674
ZNF675
ZNF676
ZNF677
ZNF678
ZNF679
ZNF680
ZNF681
ZNF682
ZNF683
ZNF684
ZNF687
ZNF688
ZNF689
ZNF69
ZNF691
ZNF692
ZNF695
ZNF696
ZNF697
ZNF699
ZNF7
ZNF70
ZNF700
ZNF701
ZNF702P
ZNF703
ZNF704
ZNF705A
ZNF705D
ZNF705G
ZNF706
ZNF707
ZNF708
ZNF709
ZNF71
ZNF710
ZNF711
ZNF713
ZNF716
ZNF717
ZNF718
ZNF720

ZNF726
ZNF727
ZNF728
ZNF729
ZNF730
ZNF738
ZNF74
ZNF740
ZNF746
ZNF747
ZNF749
ZNF750
ZNF75A
ZNF75D
ZNF76
ZNF761
ZNF763
ZNF764
ZNF765
ZNF766
ZNF767
ZNF767P
ZNF768
ZNF77
ZNF770
ZNF771
ZNF773
ZNF774
ZNF775
ZNF776
ZNF777
ZNF778
ZNF780A
ZNF781
ZNF782
ZNF783
ZNF784
ZNF785
ZNF786
ZNF787
ZNF788
ZNF789
ZNF79
ZNF790
ZNF790-AS1
ZNF791
ZNF792
ZNF793
ZNF799
ZNF8

ZNF80
ZNF800
ZNF804A
ZNF804B
ZNF805
ZNF808
ZNF81
ZNF812
ZNF813
ZNF814
ZNF815
ZNF816A
ZNF821
ZNF823
ZNF827
ZNF828
ZNF829
ZNF83
ZNF830
ZNF831
ZNF833
ZNF835
ZNF836
ZNF837
ZNF839
ZNF84
ZNF841
ZNF843
ZNF844
ZNF845
ZNF846
ZNF852
ZNF853
ZNF860
ZNF862
ZNF865
ZNF876P
ZNF878
ZNF879
ZNF880
ZNF888
ZNF890P
ZNF891
ZNF90
ZNF92
ZNF93
ZNF98
ZNFX1
ZNHIT1
ZNHIT2

ZNHIT3
ZNHIT6
ZNRD1
ZNRF1
ZNRF2
ZNRF3
ZNRF3-AS1
ZNRF4
ZP1
ZP2
ZP3
ZP4
ZPBP
ZPBP2
ZPLD1
ZRANB1
ZRANB2
ZRANB2-AS1
ZRANB2-AS2
ZRANB3
ZRSR2
ZSCAN1
ZSCAN10
ZSCAN12
ZSCAN16
ZSCAN18
ZSCAN2
ZSCAN20
ZSCAN21
ZSCAN22
ZSCAN23
ZSCAN25
ZSCAN26
ZSCAN31
ZSCAN32
ZSCAN4
ZSCAN5A
ZSCAN9
ZSWIM1
ZSWIM2
ZSWIM3
ZSWIM4
ZSWIM5
ZSWIM6
ZSWIM8
ZSWIM8-AS1
ZUFSP
ZW10
ZWILCH
ZWINT

ZXDA
ZXDB
ZXDC
ZYG11A
ZYG11B
ZYX
ZZEF1
ZZZ3
